# Genetic Diversity of *Mycobacterium tuberculosis* in Peru and Exploration of Phylogenetic Associations with Drug Resistance

**DOI:** 10.1371/journal.pone.0065873

**Published:** 2013-06-24

**Authors:** Patricia Sheen, David Couvin, Louis Grandjean, Mirko Zimic, Maria Dominguez, Giannina Luna, Robert H. Gilman, Nalin Rastogi, David A. J. Moore

**Affiliations:** 1 Laboratorio de Enfermedades Infecciosas, Laboratorios de Investigación y Desarrollo, Facultad de Ciencias y Filosofía, Universidad Peruana Cayetano Heredia, Lima, Peru; 2 WHO Supranational TB Reference Laboratory, TB and Mycobacteria Unit, Institut Pasteur de la Guadeloupe, Guadeloupe, France; 3 LSHTM TB Centre and Department of Clinical Research, London School of Hygiene and Tropical Medicine, London, United Kingdom; 4 Department of Infectious Diseases and Immunity, Imperial College, London, United Kingdom; 5 Department of International Health, Johns Hopkins Bloomberg School of Public Health, Baltimore, Maryland, United States of America; St. Petersburg Pasteur Institute, Russian Federation

## Abstract

**Background:**

There is limited available data on the strain diversity of *M tuberculosis* in Peru, though there may be interesting lessons to learn from a setting where multidrug resistant TB has emerged as a major problem despite an apparently well-functioning DOTS control programme.

**Methods:**

Spoligotyping was undertaken on 794 strains of M tuberculosis collected between 1999 and 2005 from 553 community-based patients and 241 hospital-based HIV co-infected patients with pulmonary tuberculosis in Lima, Peru. Phylogenetic and epidemiologic analyses permitted identification of clusters and exploration of spoligotype associations with drug resistance.

**Results:**

Mean patient age was 31.9 years, 63% were male and 30.4% were known to be HIV+. Rifampicin mono-resistance, isoniazid mono-resistance and multidrug resistance (MDR) were identified in 4.7%, 8.7% and 17.3% of strains respectively. Of 794 strains from 794 patients there were 149 different spoligotypes. Of these there were 27 strains (3.4%) with novel, unique orphan spoligotypes. 498 strains (62.7%) were clustered in the nine most common spoligotypes: 16.4% SIT 50 (clade H3), 12.3% SIT 53 (clade T1), 8.3% SIT 33 (LAM3), 7.4% SIT 42 (LAM9), 5.5% SIT 1 (Beijing), 3.9% SIT 47 (H1), 3.0% SIT 222 (clade unknown), 3.0% SIT1355 (LAM), and 2.8% SIT 92 (X3). Amongst HIV-negative community-based TB patients no associations were seen between drug resistance and specific spoligotypes; in contrast HIV-associated MDRTB, but not isoniazid or rifampicin mono-resistance, was associated with SIT42 and SIT53 strains.

**Conclusion:**

Two spoligotypes were associated with MDR particularly amongst patients with HIV. The MDR-HIV association was significantly reduced after controlling for SIT42 and SIT53 status; residual confounding may explain the remaining apparent association. These data are suggestive of a prolonged, clonal, hospital-based outbreak of MDR disease amongst HIV patients but do not support a hypothesis of strain-specific propensity for the acquisition of resistance-conferring mutations.

## Introduction

Molecular fingerprinting of *M. tuberculosis* (MTB) permits investigation of the epidemiology of tuberculosis to a previously unattainable level of detail, revealing insights into the differential transmission success of strains whilst observation and analysis of this epidemiology can generate testable hypotheses about strain biology [1]. There is limited data on the epidemiology and strain diversity of *M tuberculosis* in Peru [2], though there may be interesting lessons to learn from a setting where multidrug resistant TB has emerged as a major problem despite an apparently well-functioning DOTS control programme [3]. Here we report the results of an exercise to spoligotype all the strains of a large bank of well-characterized MTB strains derived from research projects conducted in Lima, Peru between 1999 and 2005, conduct spoligotype-based phylogenetic analyses and explore phylogenetic associations with HIV infection and drug resistance.

## Methods

### Strain bank

The sampling framework for this study was opportunistic, making use of a strain bank of anonymised but phenotypically well characterized strains of *M tuberculosis* collected in the course of four clinical research studies conducted amongst adults with pulmonary tuberculosis in hospital and community-based studies in Lima, Peru. All studies had been reviewed and approved by the Institutional Review Board of Universidad Peruana Cayetano Heredia (Lima, Peru). Spoligotyping was undertaken on 794 strains of *M tuberculosis* (1 per patient) which came from (1) an unselected community-based cohort from south Lima (Feb 1999–May 2002) (n = 329), (2) a hospital-based cohort from an HIV unit (May 1999–Feb 2005) (n = 241), (3) an unselected community-based cohort from north Lima (June 2003–July 2004) (n = 155), (4) a selected community-based cohort from east Lima of TB patients reporting conventional risk factors for drug-resistant TB (Nov 2003–Oct 2005) (n = 69). Recruitment for each of these studies has been reported previously [4], [5], [6]. Strains were stored at −70°C in the Laboratorio de Investigación de Enfermedades Infecciosas of Universidad Peruana Cayetano Heredia (UPCH) in Lima. Available patient data was limited to gender, age and HIV status; strain data included first line drug susceptibility profile and name of source study with date of collection of original clinical sample.

Spoligotyping was undertaken at UPCH in batches over several months and films were interpreted by two independent readers; for the rare occasions where there was lack of independent agreement and subsequent failure to resolve discrepancies between both readers spoligotyping was repeated and the new film was used. Phylogenetic analyses and the construction of phylogenetic trees and spoligoforests (drawn using the SpolTools software available through http://www.emi.unsw.edu.au/spolTools; [7], [8]) permitted identification of clusters and orphan strains by comparison with the SITVIT2 database (Institut Pasteur de Guadeloupe). The minimum spanning tree (MST), based on spoligotyping patterns, was drawn using BioNumerics software version 3.5. The MST is an undirected connected graph which links all the strains together with the fewest possible linkages between nearest neighbours. Contrarily to the MST, the spoligoforest trees are directed graphs which only evolve by loss of spacers. In these trees, nodes are not necessarily all connected (indeed, in case of too many changes between two strains, there are no edges linking them. In combination with SpolTools software, GraphViz software (http://www.graphviz.org) [9] was used to colour the orphan strains on the spoligoforest trees. Strains were categorized into spoligotype international types (SIT) and clades for the purpose of reporting strain diversity within the strain bank. In a univariate analysis odds ratios were computed for associations between strain groupings (by clade and by SIT separately) and patient gender, HIV status, strain year of origin, isoniazid mono-resistance, rifampicin mono-resistance and multidrug resistance (with each compared to drug susceptible reference group); in subsequent multivariate logistic regression only those clade or SIT associations with a p value<0.1 on univariate analysis were included in the model.

## Results

### Study population

Mean patient age was 31.9 years (range 15–78, no gender difference), 63% were male and 30.4% were known to be HIV+. HIV infection was significantly more frequent amongst males (OR 3.00, 95% CI 2.1–4.3). Rifampicin mono-resistance, isoniazid mono-resistance and multidrug resistance (MDR) were identified in 4.7%, 8.7% and 17.3% of tested strains respectively (12 rifampicin and 20 isoniazid results were unavailable).

### Strain diversity

Of 794 strains from 794 patients there were 149 different spoligotypes identified. Of these there were 27 strains with novel, unique orphan spoligotypes, 718 which mapped to spoligotypes already described at least twice, and 49 which were newly created shared types either within the present study or after a match with an orphan in the database. Descriptions of the orphan strain spoligotypes and the demographics of the source patients are given in table 1. In table 2 the 122 already known spoligotype international types (SITs) and corresponding lineages detected in this strain set are shown along with the frequency that each occurred in this strain set as compared with the comparison global database. (The complete original dataset of 794 spoligotypes with accompanying clinical data is made available in table S1; the comparative frequency of the predominant SITs in this study with those reported elsewhere in Latin America is shown in table S2).

**Table 1:** Description of spoligotypes with corresponding spoligotyping defined lineages/sublineages and demographic information for 27 orphan strains identified from amongst 794 strains of *M. tuberculosis* isolated from adults with pulmonary tuberculosis in Lima, Peru.

Year	Strain	Spoligotype Description	Octal code	Lineage *	Drug-R **	Sex/Age	HIV Serology
2003	0075	▪□□□□□▪▪▪▪▪▪▪▪▪▪▪▪▪▪□□□□▪▪□□□□□□□□□□□□□□▪▪▪	407777606000031	Unknown	1	M/48	
2003	0095	▪▪▪▪▪▪▪▪□□□▪▪▪▪▪▪▪▪▪□□□□□□□▪□▪▪▪□□□□▪▪▪▪▪▪▪	776177600560771	T1	1	F/27	
2003	0112	▪□□▪▪▪▪▪▪▪▪▪▪▪▪▪▪▪▪▪▪□□▪▪▪▪▪▪▪▪▪□□□□▪▪▪□▪▪▪	477777717760731	T2	1	M/50	
2003	0127	▪▪▪▪▪▪▪▪▪▪▪▪□▪▪□▪▪▪▪▪▪▪▪▪▪▪▪▪▪▪▪□□□□▪▪▪□▪▪▪	777733777760731	T	1	F/53	
2003	0129	▪▪□□▪▪▪▪□□□▪▪▪▪▪▪▪▪□□□□□□□□□□□□□□□□□□□▪▪▪▪▪	636177400000171	Unknown	1	F/78	
2004	0165	▪▪▪□▪▪▪▪▪▪▪▪▪▪▪▪▪▪▪▪□□□□▪▪▪▪□▪▪▪□□□□▪▪▪□▪▪▪	737777607560731	LAM6	1	F/43	
2004	0168	▪▪▪□□□□□□□□□□▪▪▪▪□▪▪▪▪▪▪▪▪▪▪▪▪▪□□□□□▪▪▪▪▪▪▪	700036777740771	X3	2	M/30	
2004	0173	▪▪▪▪▪▪▪▪□▪▪□□▪▪▪▪▪▪▪▪▪▪▪▪▪▪▪▪▪▪▪□□□□▪▪▪▪▪▪▪	776637777760771	T1	1	M/50	
2004	0178	▪▪▪▪▪▪▪□□□▪▪▪▪▪▪▪▪▪▪▪▪▪▪▪▪□□▪▪▪▪□□□□▪▪▪▪□▪▪	774377776360751	T1	1	M/18	
2004	0186	▪▪▪▪▪▪▪▪▪▪▪▪▪▪▪▪▪▪▪□▪▪□▪▪▪▪▪▪▪□□□□□□▪▪▪▪▪▪▪	777777557700771	Unknown	1	F/39	
1999	0228	▪▪□□□□□▪▪▪▪▪▪▪▪▪▪▪▪▪□□□□▪▪▪▪▪▪▪▪□□□□▪▪▪▪▪▪▪	603777607760771	LAM1	1	M/26	HIV+
1999	0239	▪▪▪▪▪▪▪▪□□□▪▪□□□□▪▪▪□□□□▪▪▪▪▪▪▪▪□□□□▪▪▪□▪▪▪	776141607760731	LAM3	1	M/26	HIV+
2000	0279	▪▪▪▪▪▪▪▪▪▪▪▪▪▪▪▪▪▪▪▪□□□□▪▪□▪□▪▪▪□□□□▪▪▪□▪▪▪	777777606560731	LAM6	1	F/22	HIV+
2000	0290	□▪▪▪▪▪▪▪▪▪▪▪▪▪▪▪▪▪▪▪□□□□▪▪□□□□□□□□□□□□□□▪▪▪	377777606000031	Unknown	1	F/29	HIV+
2001	0325	▪▪▪▪▪▪▪▪▪▪▪▪▪▪▪▪▪▪▪▪▪▪▪▪▪□▪□□□□▪□□□□□▪▪▪▪▪▪	777777775020371	H3	1	M/40	HIV+
1999	0350	▪▪□▪▪▪□□□□□□□□□□□□□□□□□□□□□□□▪▪▪□□□□▪▪▪▪▪▪▪	670000000160771	T1	1	M/24	
1999	0367	▪▪▪▪▪▪▪▪▪▪▪▪▪▪▪▪▪□▪▪▪▪▪▪▪□▪▪▪▪▪▪□□□□▪▪▪□□□□	777776775760700	X1	1	M/0	
2000	0478	▪▪▪▪▪▪▪▪▪▪▪▪▪▪▪▪▪▪▪▪▪▪▪▪▪▪▪▪▪▪□□□□□□▪▪▪□▪▪▪	777777777700731	Unknown	1	F/0	
2000	0498	▪▪▪▪▪▪▪□□▪▪▪▪▪▪▪▪▪▪▪▪▪▪▪▪▪▪▪▪▪▪▪□□□□▪▪▪□▪▪▪	774777777760731	T2	1	M/0	
2000	0519	▪▪▪▪▪▪▪▪▪▪▪▪▪▪▪▪▪□▪▪▪▪▪▪▪□▪▪▪▪▪▪□□□□▪▪□□□□□	777776775760600	X2	1	F/0	
2000	0570	▪▪▪□□□▪□□□□□□□□□▪▪□▪▪▪□□▪▪▪▪▪▪▪▪□□□□▪□□□□□□	704003347760400	T4	1	M/0	
2001	0593	▪▪▪▪▪▪▪▪▪▪▪▪▪□□□▪▪▪▪□▪▪▪▪▪▪▪▪▪▪▪□□□□▪▪▪▪▪▪▪	777743677760771	T1	1	M/23	
2002	0668	▪▪□□□▪▪▪▪▪▪▪▪▪▪▪▪▪▪▪▪▪▪▪▪▪▪▪▪▪□□□□□□▪▪▪▪▪▪▪	617777777700771	Unknown	1	M/25	
2002	0671	▪▪▪▪□□□□▪▪▪▪▪▪▪▪□□□□□▪▪▪▪▪▪▪□▪▪▪□□□□▪▪▪▪▪▪▪	741774077560771	T1	1	F/36	
2002	0705	▪▪▪▪▪▪▪▪□□▪▪▪▪▪▪▪▪▪▪▪▪▪□□□▪▪▪▪▪▪□□□□▪▪▪▪▪▪▪	776377761760771	S	1	M/28	HIV+
2003	0719	▪▪□□□▪▪▪▪▪▪▪□▪▪▪▪▪▪▪▪▪▪▪▪▪▪▪▪▪□▪□□□□▪▪▪▪▪▪▪	617737777720771	H3	1	M/32	HIV+
2004	0796	▪▪▪▪▪▪▪▪▪▪▪▪▪▪▪▪□□□□□▪▪▪▪▪▪▪□▪▪▪□□□□▪▪▪□□▪▪	777774077560711	T1	1	M/34	HIV+

* Lineage designations for orphan patterns were done manually as Expert-based interpretations using revised SpolDB4 rules.

** Drug-R code: 1, pansusceptible; 2, MDR (combined resistance to INH-RIF); 3, any other resistance; 4, XDR-TB (combined resistance to INH, RIF, fluoroquinolones, and 1 of 3 injectable drugs, i.e., capreomycin, kanamycin, or amikacin).

**Table 2:** Description of 122 shared-types (SITs; n = 767 isolates) and corresponding spoligotyping defined lineages/sublineages starting from a total of 794 *M. tuberculosis* strains isolated from adults with pulmonary tuberculosis in Lima, Peru.

SIT *	Spoligotype Description	Octal Number	Number (%) in study	% in study vs. database	Lineage**	Clustered vs. unique patterns***
1	□□□□□□□□□□□□□□□□□□□□□□□□□□□□□□□□□□▪▪▪▪▪▪▪▪▪	000000000003771	44 (5.54)	0.46	Beijing	Clustered
4	□□□□□□□□□□□□□□□□□□□□□□□□▪▪▪▪▪▪▪▪□□□□▪▪▪▪▪▪▪	000000007760771	2 (0.25)	0.6	Unknown	Clustered
11	▪□□▪▪▪▪▪▪▪▪▪▪▪▪▪▪▪▪▪▪▪▪▪▪▪▪▪□□□□▪□▪▪□□□▪▪▪▪	477777777413071	1 (0.13)	0.18	EAI3-IND	Unique
19	▪▪□▪▪▪▪▪▪▪▪▪▪▪▪▪▪▪▪□□▪▪▪▪▪▪▪□□□□▪□▪▪▪▪▪▪▪▪▪	677777477413771	1 (0.13)	0.12	EAI2-Manilla	Unique
20	▪▪□▪▪▪▪▪▪▪▪▪▪▪▪▪▪▪▪▪□□□□▪▪▪▪▪▪▪▪□□□□▪▪▪▪▪▪▪	677777607760771	4 (0.5)	0.51	LAM1	Clustered
33	▪▪▪▪▪▪▪▪□□□▪▪▪▪▪▪▪▪▪□□□□▪▪▪▪▪▪▪▪□□□□▪▪▪▪▪▪▪	776177607760771	66 (8.31)	6.05	LAM3	Clustered
36	▪▪▪▪▪▪▪▪▪▪▪▪□▪▪▪▪▪▪▪▪▪▪▪▪▪▪▪▪▪□▪□□□□▪▪▪▪▪▪▪	777737777720771	4 (0.5)	3.51	H3	Clustered
39	▪▪▪▪▪▪▪▪▪▪▪▪▪▪▪▪▪▪□▪▪▪□□▪▪▪▪▪▪▪▪□□□□▪□□▪▪▪▪	777777347760471	2 (0.25)	1.49	T4-CEU1	Clustered
42	▪▪▪▪▪▪▪▪▪▪▪▪▪▪▪▪▪▪▪▪□□□□▪▪▪▪▪▪▪▪□□□□▪▪▪▪▪▪▪	777777607760771	59 (7.43)	1.92	LAM9	Clustered
46	▪▪▪▪▪▪▪▪▪▪▪▪▪▪▪▪▪▪▪▪▪▪▪▪□□□□□□□□□□□□□□□□□□□	777777770000000	1 (0.13)	0.54	Unknown	Unique
47	▪▪▪▪▪▪▪▪▪▪▪▪▪▪▪▪▪▪▪▪▪▪▪▪▪□□□□□□▪□□□□▪▪▪▪▪▪▪	777777774020771	31 (3.9)	2.24	H1	Clustered
49	▪▪▪▪▪▪▪▪▪▪▪▪▪▪▪▪▪▪▪▪▪▪▪▪▪▪▪▪▪▪□▪□□□□▪▪▪□▪▪▪	777777777720731	7 (0.88)	4.43	H3	Clustered
50	▪▪▪▪▪▪▪▪▪▪▪▪▪▪▪▪▪▪▪▪▪▪▪▪▪▪▪▪▪▪□▪□□□□▪▪▪▪▪▪▪	777777777720771	130 (16.37)	4.17	H3	Clustered
51	▪▪▪▪▪▪▪▪▪▪▪▪▪▪▪▪▪▪▪▪▪▪▪▪▪▪▪▪▪▪▪▪□□□□▪▪▪□□□□	777777777760700	1 (0.13)	0.38	T1	Unique
53	▪▪▪▪▪▪▪▪▪▪▪▪▪▪▪▪▪▪▪▪▪▪▪▪▪▪▪▪▪▪▪▪□□□□▪▪▪▪▪▪▪	777777777760771	98 (12.34)	1.74	T1	Clustered
54	▪▪▪▪▪▪▪▪▪▪▪▪▪▪▪▪▪▪▪▪▪▪▪▪▪▪▪▪▪▪▪▪□□▪▪▪▪▪▪▪▪▪	777777777763771	1 (0.13)	0.46	MANU2	Unique
58	▪▪▪▪▪▪▪▪▪▪▪▪▪▪▪▪▪▪▪□▪▪□▪▪▪▪▪▪▪▪▪□□□□▪▪▪▪▪▪▪	777777557760771	8 (1.01)	4.94	T5-Madrid2	Clustered
60	▪▪▪▪▪▪▪▪▪▪▪▪▪▪▪▪▪▪▪▪□□□□▪▪▪▪▪▪▪▪□□□□▪▪▪□▪▪▪	777777607760731	2 (0.25)	0.51	LAM4	Clustered
62	▪▪▪▪▪▪▪▪▪▪▪▪▪▪▪▪▪▪▪▪▪▪▪▪▪□□□□□□▪□□□□▪▪▪□▪▪▪	777777774020731	1 (0.13)	0.2	H1	Unique
64	▪▪▪▪▪▪▪▪▪▪▪▪▪▪▪▪▪▪▪▪□□□□▪▪▪▪□▪▪▪□□□□▪▪▪▪▪▪▪	777777607560771	6 (0.76)	1.75	LAM6	Clustered
73	▪▪▪▪▪▪▪▪▪▪▪▪□▪▪▪▪▪▪▪▪▪▪▪▪▪▪▪▪▪▪▪□□□□▪▪▪□▪▪▪	777737777760731	2 (0.25)	0.84	T	Clustered
78	▪▪▪▪▪▪▪▪▪▪▪▪▪▪▪▪▪▪▪▪▪▪▪▪▪▪▪▪▪▪▪▪□□□□▪▪▪□□▪▪	777777777760711	1 (0.13)	1.61	T	Unique
86	▪▪▪▪▪▪▪▪▪▪▪▪▪▪▪▪▪▪▪▪▪□▪▪▪▪▪▪▪▪▪▪□□□□▪▪▪▪▪▪▪	777777737760771	1 (0.13)	1.19	T1	Unique
91	▪▪▪□□□□□□□□□□▪▪▪▪□▪▪▪▪▪▪▪▪▪▪▪▪▪▪□□□□▪▪▪▪▪▪▪	700036777760771	22 (2.77)	8.76	X3	Clustered
92	▪▪▪□□□□□□□□□▪▪▪▪▪□▪▪▪▪▪▪▪▪▪▪▪▪▪▪□□□□▪▪▪▪▪▪▪	700076777760771	5 (0.63)	1.18	X3	Clustered
93	▪▪▪▪▪▪▪▪▪▪▪▪□▪▪▪▪▪▪▪□□□□▪▪▪▪▪▪▪▪□□□□▪▪▪▪▪▪▪	777737607760771	12 (1.51)	3.55	LAM5	Clustered
95	▪▪▪▪▪▪▪▪▪▪▪▪▪▪▪▪▪▪▪▪□□□□▪▪▪▪□▪▪▪□□□□▪▪▪□▪▪▪	777777607560731	2 (0.25)	4.55	LAM6	Clustered
99	▪▪▪▪□▪▪▪▪▪▪▪▪▪▪▪▪▪▪▪▪▪▪▪▪▪▪▪▪▪□▪□□□□▪▪▪▪▪▪▪	757777777720771	2 (0.25)	2.94	H3	Clustered
106	▪▪▪▪▪▪▪▪□□□▪▪▪▪▪▪▪▪□□□□□□□□□□□□□□□□□□□▪▪▪▪▪	776177400000171	2 (0.25)	1.55	Unknown	Clustered
119	▪▪▪▪▪▪▪▪▪▪▪▪▪▪▪▪▪□▪▪▪▪▪▪▪▪▪▪▪▪▪▪□□□□▪▪▪▪▪▪▪	777776777760771	4 (0.5)	0.37	X1	Clustered
130	▪▪▪▪▪▪▪▪□□□▪▪▪▪▪▪▪▪▪□□□□▪▪▪▪▪▪▪▪□□□□▪▪▪□▪▪▪	776177607760731	7 (0.88)	6.67	LAM3	Clustered
132	▪▪▪▪▪▪▪▪▪▪▪▪▪▪▪▪▪▪▪▪□□□□▪▪□□□□□□□□□□□□□□▪▪▪	777777606000031	1 (0.13)	6.25	Unknown	Unique
177	□▪▪▪▪▪▪▪▪▪▪▪▪▪▪▪▪▪▪▪□□□□▪▪▪▪▪▪▪▪□□□□▪▪▪▪▪▪▪	377777607760771	1 (0.13)	1.08	LAM9	Unique
183	▪▪▪▪▪▪▪▪▪▪▪▪▪▪▪▪▪▪□▪▪▪▪▪▪▪▪▪▪▪□▪□□□□▪▪▪▪▪▪▪	777777377720771	3 (0.38)	5.77	H3	Clustered
190	□□□□□□□□□□□□□□□□□□□□□□□□□□□□□□□□□□▪▪▪▪▪□▪▪▪	000000000003731	1 (0.13)	0.56	Beijing	Unique
211	▪▪▪▪▪▪▪▪□□□▪□▪▪▪▪▪▪▪□□□□▪▪▪▪▪▪▪▪□□□□▪▪▪▪▪▪▪	776137607760771	1 (0.13)	1.23	LAM3	Unique
215	▪▪▪▪▪▪▪▪▪▪▪▪□□□□▪▪▪▪▪▪▪▪▪▪▪□▪▪▪▪□□□□▪▪▪▪▪▪▪	777703777360771	1 (0.13)	14.29	T	Unique
216	▪▪▪▪▪▪▪▪▪▪▪▪□□▪▪▪▪▪▪□□□□▪▪▪▪▪▪▪▪□□□□▪▪▪▪▪▪▪	777717607760771	3 (0.38)	15	LAM5	Clustered
219	▪▪▪▪▪▪▪▪▪▪▪▪▪□□□□□▪▪▪▪▪▪▪▪▪▪▪▪▪▪□□□□▪▪▪▪▪▪▪	777740777760771	5 (0.63)	13.16	T1	Clustered
222	▪▪▪▪▪▪▪▪▪▪▪▪▪▪▪▪□□□□□▪▪▪▪▪▪▪□▪▪▪□□□□▪▪▪▪▪▪▪	777774077560771	24 (3.02)	46.15	Unknown	Clustered
237	▪▪▪▪▪▪▪▪▪▪▪▪▪▪▪▪▪▪▪▪▪▪▪▪▪▪▪▪▪▪□□□□□□□□□□□□□	777777777700000	4 (0.5)	3.88	Unknown	Clustered
239	▪▪▪▪▪▪▪▪▪▪▪▪▪▪▪▪▪▪▪▪▪▪▪▪▪▪▪▪▪▪▪▪□□□□□□□□▪▪▪	777777777760031	2 (0.25)	3.77	T2	Clustered
283	▪▪▪▪▪▪▪▪▪▪▪▪▪▪▪▪▪▪▪▪▪□□□▪□□□□□□▪□□□□▪▪▪▪▪▪▪	777777704020771	1 (0.13)	1.75	H1	Unique
373	▪▪▪▪▪▪▪▪▪▪▪▪▪▪▪▪▪▪▪▪▪▪▪□▪▪▪▪▪▪▪▪□□□□▪▪▪▪▪▪▪	777777767760771	2 (0.25)	3.33	T1	Clustered
384	▪▪▪▪▪▪▪▪▪▪▪▪▪▪▪▪▪▪▪▪□▪▪▪▪□□□□□□▪□□□□▪▪▪▪▪▪▪	777777674020771	1 (0.13)	11.11	H1	Unique
390	▪▪▪▪▪▪▪▪▪▪▪▪▪▪▪▪▪▪▪▪▪▪▪▪▪▪▪▪▪□□▪□□□□▪▪▪▪▪▪▪	777777777620771	3 (0.38)	10.34	H3	Clustered
396	▪▪▪▪▪▪▪▪▪▪▪▪▪▪▪▪▪▪▪▪□□□□▪▪▪▪□▪▪▪□□□□□▪▪▪▪▪▪	777777607560371	2 (0.25)	11.76	LAM6	Clustered
418	▪▪□□□▪▪▪▪▪▪▪▪▪▪▪▪▪▪▪▪▪▪▪▪▪▪▪▪▪□▪□□□□▪▪▪▪▪▪▪	617777777720771	9 (1.13)	64.29	H3	Clustered
430	▪▪▪□□□▪□□□□□□□□□▪▪□▪▪▪□□▪▪▪▪▪▪▪▪□□□□▪□□▪▪▪▪	704003347760471	4 (0.5)	21.05	T4-CEU1	Clustered
450	▪▪▪▪▪▪▪▪▪▪▪▪▪▪▪▪▪□▪▪▪▪▪▪□□□□□□□□□□□□□□□□□□□	777776770000000	14 (1.76)	15.05	Unknown	Clustered
469	□□□▪▪▪▪▪▪▪▪▪▪▪▪▪▪▪▪▪□□□□▪▪▪▪▪▪▪▪□□□□▪▪▪▪▪▪▪	077777607760771	1 (0.13)	3.57	LAM1	Unique
489	▪▪▪▪▪□▪▪▪▪▪▪▪▪▪▪▪□▪▪▪▪▪▪▪▪▪▪▪▪▪▪□□□□▪▪□□□□▪	767776777760601	1 (0.13)	9.09	X2	Unique
512	▪▪▪▪▪▪▪▪▪▪▪▪▪▪▪▪▪▪▪▪▪□□□▪▪▪▪▪▪□▪□□□□▪▪▪▪▪▪▪	777777707720771	4 (0.5)	13.79	H3	Clustered
534	▪▪▪▪▪▪▪▪▪▪▪▪▪▪▪▪▪▪▪▪□□□□▪▪▪▪□□□□□□□□□□□□□□□	777777607400000	1 (0.13)	8.33	LAM	Unique
546	▪▪▪□□□□□□□□□□▪▪▪▪□▪▪▪▪▪▪▪▪▪▪□▪▪▪□□□□▪▪▪▪▪▪▪	700036777560771	1 (0.13)	7.14	X3	Unique
559	▪▪▪▪▪▪▪▪□□▪▪▪▪▪▪▪▪▪▪▪▪▪▪□□□▪▪▪▪▪□□□□▪▪▪▪▪▪▪	776377770760771	1 (0.13)	16.67	S	Unique
620	▪▪▪▪▪▪▪▪▪▪▪▪▪▪▪▪▪▪▪▪▪▪□□▪□□□□□□▪□□□□▪▪▪▪▪▪▪	777777744020771	2 (0.25)	14.29	H1	Clustered
644	□▪□□□□□□□□□□□□□□▪▪□▪▪▪▪▪▪▪▪□▪□□□□▪▪▪▪▪□□□□□	200003377207600	1 (0.13)	3.45	BOV_4-CAPRAE	Unique
740	▪▪▪▪▪▪▪▪▪▪▪▪▪▪▪▪▪▪▪▪▪▪□□▪▪▪▪▪▪□▪□□□□▪▪▪▪▪▪▪	777777747720771	1 (0.13)	7.69	H3	Unique
748	▪▪▪▪▪▪▪▪▪▪▪▪▪▪▪▪▪▪▪▪▪▪▪▪▪▪▪▪▪▪□▪□□□□▪▪▪▪▪□□	777777777720760	1 (0.13)	20	H3	Unique
777	▪▪▪▪▪▪▪▪▪▪▪▪▪▪▪▪▪▪▪▪▪▪▪▪▪▪▪▪□□□▪□□□□▪▪▪▪▪▪▪	777777777420771	1 (0.13)	2.08	H3	Unique
784	▪▪▪▪▪▪▪▪□□▪▪▪▪▪▪▪▪▪▪▪▪▪▪▪▪▪▪▪▪▪▪□□□□▪▪▪□▪▪▪	776377777760731	1 (0.13)	1.82	S	Unique
786	▪▪▪▪▪▪▪▪▪▪▪▪▪▪▪▪▪▪▪▪▪▪▪□□□□□□□□□□□□□□□□□□□□	777777760000000	3 (0.38)	23.08	Unknown	Clustered
826	▪▪□▪▪▪▪▪▪▪▪▪□▪▪▪▪▪□▪□□□□▪▪▪▪▪▪▪▪□□□□▪▪▪▪▪▪▪	677737207760771	4 (0.5)	44.44	LAM2	Clustered
849	▪▪□□▪▪▪▪▪▪▪▪▪▪▪▪▪▪▪▪▪▪▪▪▪▪▪▪▪▪□▪□□□□▪▪▪▪▪▪▪	637777777720771	2 (0.25)	20	H3	Clustered
867	▪▪▪▪▪▪▪▪▪▪▪▪□▪▪▪▪▪▪▪□□□□▪▪▪▪□▪▪▪□□□□▪▪▪□▪▪▪	777737607560731	1 (0.13)	7.69	LAM	Unique
893	▪▪▪▪▪▪▪▪▪▪▪▪▪▪▪▪▪▪□□□□□▪▪▪▪▪▪▪▪▪□□□□▪▪▪▪▪▪▪	777777017760771	3 (0.38)	33.33	T	Clustered
914	▪▪▪▪▪▪▪▪□□▪▪▪▪▪▪▪▪▪▪▪▪▪▪▪▪▪▪▪▪□▪□□□□▪▪▪▪▪▪▪	776377777720771	2 (0.25)	5.26	Unknown	Clustered
1080	▪▪▪▪▪▪▪▪▪▪▪▪▪▪▪▪▪□▪▪▪▪▪▪▪▪▪▪▪▪▪▪□□□□▪▪▪□□□▪	777776777760701	1 (0.13)	14.29	X1	Unique
1105	▪▪▪▪▪▪▪▪▪▪▪▪▪▪▪□▪▪▪▪▪▪▪▪▪▪▪▪▪▪▪▪□□□□▪▪▪▪▪▪▪	777773777760771	8 (1.01)	29.63	T1	Clustered
1122	▪▪▪▪▪□▪▪▪▪▪▪▪▪▪▪▪▪▪▪▪▪▪▪▪▪▪▪▪▪▪▪□□□□▪▪▪▪▪▪▪	767777777760771	1 (0.13)	2.38	T1	Unique
1139	▪▪▪▪▪▪▪▪▪▪▪▪▪▪▪▪▪▪□▪▪▪▪▪▪□□□□□□▪□□□□▪▪▪▪▪▪▪	777777374020771	2 (0.25)	25	H1	Clustered
1150	▪▪▪□□□□□□□□□□▪▪▪▪□▪▪▪▪▪▪▪▪□□▪▪▪▪□□□□▪▪▪▪▪▪▪	700036776360771	2 (0.25)	50	X3	Clustered
1177	▪▪▪▪▪▪▪▪▪▪▪▪▪▪▪▪▪▪▪▪▪▪▪□▪▪▪▪▪▪□□□□□□□□□□□□□	777777767700000	1 (0.13)	25	Unknown	Unique
1214	▪▪▪▪▪▪▪▪▪▪▪□□□▪▪▪▪▪▪▪▪▪▪▪▪▪▪▪▪▪▪□□□□▪▪▪▪▪▪▪	777617777760771	1 (0.13)	5	T3	Unique
1220	▪▪▪▪□▪▪□□□▪▪▪▪▪▪▪▪▪▪□□□□▪▪▪▪▪▪▪▪□□□□▪▪▪□□□□	754377607760700	1 (0.13)	33.33	LAM	Unique
1230	▪▪▪▪▪▪▪▪▪▪▪▪▪□▪▪▪▪▪▪▪▪▪▪▪□□□□□□▪□□□□▪▪▪▪▪▪▪	777757774020771	1 (0.13)	20	H1	Unique
1235	▪▪▪▪▪▪▪▪▪▪▪▪▪▪▪▪▪▪▪▪▪▪□□▪□□▪▪▪□▪□□□□▪▪▪▪▪▪▪	777777744720771	1 (0.13)	33.33	H3	Unique
1354	▪□▪▪▪▪▪▪□□□▪▪▪▪▪▪▪▪▪□□□□▪▪▪▪▪▪▪▪□□□□▪▪▪▪▪▪▪	576177607760771	2 (0.25)	33.33	LAM3	Clustered
1355	▪▪▪▪▪▪▪▪▪▪▪▪▪▪▪▪▪▪▪□□□□□▪▪▪▪□▪▪▪□□□□▪▪▪□▪▪▪	777777407560731	24 (3.02)	50	LAM	Clustered
1356	▪▪▪▪▪▪▪▪□□▪▪▪▪▪▪▪▪▪▪▪▪▪▪▪▪▪▪▪▪▪▪□□□□▪▪▪▪□▪▪	776377777760751	2 (0.25)	11.76	S	Clustered
1476	▪▪▪□▪▪▪▪□□□▪▪▪▪▪▪▪▪▪□□□□▪▪▪▪▪▪□□□□□□□□▪▪▪▪▪	736177607700171	5 (0.63)	41.67	AFRI_2	Clustered
1525	□▪▪▪▪▪▪▪□□□▪□▪▪▪▪▪▪▪□□□□▪▪▪▪▪▪▪▪□□□□▪▪▪▪▪▪▪	376137607760771	1 (0.13)	20	LAM3	Unique
1552	▪▪▪▪▪▪▪▪▪▪▪▪▪▪▪▪▪▪▪▪▪▪▪▪▪□□□□□□▪□□□□▪▪□□▪▪▪	777777774020631	1 (0.13)	16.67	H1	Unique
1563	▪▪▪▪▪▪▪▪▪▪▪▪▪▪▪▪▪▪▪▪□□▪▪▪▪□▪▪▪▪▪□□□□▪▪▪▪▪▪▪	777777636760771	1 (0.13)	33.33	T1	Unique
1617	▪▪▪□□□□□□□□□□▪▪▪▪□▪▪▪▪▪▪▪▪□▪▪▪▪▪□□□□▪▪▪▪▪▪▪	700036776760771	1 (0.13)	33.33	X3	Unique
1681	□▪▪▪▪▪▪▪▪▪▪▪▪▪▪▪▪▪▪▪▪▪▪▪▪□□□□□□▪□□□□▪▪▪▪▪▪▪	377777774020771	1 (0.13)	20	H1	Unique
1708	▪▪▪▪▪▪▪▪□▪▪▪▪▪▪▪▪▪▪▪□□□□▪▪▪▪▪▪▪▪□□□□▪▪▪▪▪▪▪	776777607760771	1 (0.13)	33.33	LAM9	Unique
1999	▪▪▪▪▪▪▪▪▪▪▪▪□□□□□▪▪▪□□□□▪▪▪▪▪▪▪▪□□□□▪▪▪▪▪▪▪	777701607760771	1 (0.13)	16.67	LAM5	Unique
2028	▪▪▪▪▪▪▪▪▪▪▪▪▪▪▪▪▪▪▪▪□□□□▪□□□□□□▪□□□□▪▪▪▪▪▪▪	777777604020771	1 (0.13)	20	Unknown	Unique
2054	▪▪□▪▪▪▪▪▪▪▪▪▪▪▪▪▪▪▪▪□□□□▪▪▪▪▪▪▪▪□□□□□▪▪▪▪▪▪	677777607760371	1 (0.13)	20	LAM	Unique
2179	▪▪▪▪▪▪▪▪□□▪▪▪▪▪▪▪▪▪▪▪▪▪□□□□□▪▪▪▪□□□□▪▪▪▪▪▪▪	776377760360771	1 (0.13)	20	S	Unique
2230	▪▪▪▪▪▪▪▪▪▪▪▪□□□□□□□□□▪▪▪▪▪▪▪▪▪▪▪□□□□▪▪▪▪▪▪▪	777700077760771	1 (0.13)	25	Unknown	Unique
2274	□□□□▪▪▪▪▪▪▪▪▪▪▪▪▪▪▪□□□□□▪▪▪▪□▪▪▪□□□□▪▪▪□▪▪▪	037777407560731	7 (0.88)	63.64	LAM	Clustered
2381	▪▪▪▪▪▪▪▪▪▪▪□▪▪▪▪▪▪▪▪▪▪▪▪▪▪▪▪▪▪□▪□□□□▪▪▪▪▪▪▪	777677777720771	1 (0.13)	12.5	H3	Unique
2383	▪▪▪▪□▪▪▪▪▪▪▪□▪▪▪▪▪▪▪□□□□▪▪▪▪▪▪▪▪□□□□▪▪▪▪▪▪▪	757737607760771	1 (0.13)	20	LAM5	Unique
2626	▪▪▪▪▪▪▪▪□□□▪▪▪▪▪▪▪▪▪□□□□▪▪▪▪▪▪□□□□□□□□▪▪▪▪▪	776177607700171	1 (0.13)	25	LAM3	Unique
2744	▪▪▪▪▪▪▪▪▪▪▪▪▪▪▪▪▪▪▪▪▪▪▪▪▪▪▪▪▪▪□□□□□□□□□▪▪▪▪	777777777700071	1 (0.13)	33.33	Unknown	Unique
2885	▪▪▪▪▪▪▪▪▪▪▪▪▪▪▪▪▪▪▪□□▪▪▪▪▪▪▪▪▪□▪□□□□▪▪▪▪▪▪▪	777777477720771	1 (0.13)	9.09	H3	Unique
2916	▪▪▪▪▪▪▪▪□□▪▪▪▪▪▪▪▪▪▪▪▪▪▪▪▪▪□▪▪▪▪□□□□▪▪▪▪▪▪▪	776377777360771	1 (0.13)	25	S	Unique
2961*	▪□□□□□□□□□□□□□□□□□□□□▪▪▪□□▪▪▪▪▪▪□□□□▪□▪▪▪▪▪	400000071760571	2 (0.25)	100.0	Unknown	Clustered
3000*	▪▪▪▪□▪▪▪▪▪▪▪▪▪▪▪▪▪▪□□□□□▪▪▪▪□▪▪▪□□□□▪▪▪□▪▪▪	757777407560731	3 (0.38)	100.0	LAM	Clustered
3001*	▪▪▪▪▪▪▪▪▪▪▪▪▪▪▪▪□□□□□▪▪▪▪▪▪□□□□▪□□□□▪▪▪▪▪▪▪	777774077020771	8 (1.01)	88.9	H3	Clustered
3004*	▪▪▪▪▪▪▪▪□□▪▪▪▪▪▪▪▪▪▪▪▪▪▪▪▪▪□□□□□□□□□□□□□□□□	776377777000000	2 (0.25)	100.0	Unknown	Clustered
3005*	▪▪□□□□□▪▪▪▪▪▪▪▪▪▪▪▪▪□▪▪▪▪▪▪▪□▪▪▪□□□□▪▪▪▪▪▪▪	603777677560771	2 (0.25)	100.0	T	Clustered
3006*	▪□▪▪▪▪▪▪▪▪▪▪▪▪▪▪▪▪▪▪□□□□▪▪▪▪▪▪▪▪□□□□▪▪▪□▪▪▪	577777607760731	2 (0.25)	66.7	LAM4	Clustered
3007*	▪▪▪▪▪▪▪▪□□▪▪▪▪▪▪▪▪▪▪▪▪▪▪□□□□▪▪▪▪□□□□▪▪▪▪▪▪▪	776377770360771	1 (0.13)	50.0	S	Unique
3008*	▪▪▪▪▪▪▪▪□▪▪▪▪▪▪▪▪▪▪▪▪▪▪▪▪□□□□□□▪□□□□▪▪▪▪▪▪▪	776777774020771	1 (0.13)	50.0	H1	Unique
3009*	▪▪▪▪▪▪▪▪▪▪▪▪▪▪▪□▪▪▪▪▪▪▪▪▪▪▪▪▪▪▪▪□□□□□□▪▪▪▪▪	777773777760171	2 (0.25)	100.0	T1	Clustered
3010*	▪▪▪▪▪▪▪▪□□▪▪▪▪▪▪▪▪▪▪▪▪▪▪▪▪▪▪▪▪▪□□□□□▪▪▪□▪▪▪	776377777740731	1 (0.13)	50.0	S	Unique
3011*	▪▪▪▪□□□▪▪▪▪▪▪▪▪▪□□□□□▪▪▪▪▪▪▪□▪▪▪□□□□▪▪▪□▪▪▪	743774077560731	4 (0.5)	100.0	Unknown	Clustered
3012*	▪▪▪▪▪▪▪▪▪▪▪▪▪▪▪▪□□□□□▪▪▪□□□□□□□□□□□□□□▪▪▪▪▪	777774070000171	2 (0.25)	100.0	Unknown	Clustered
3013*	□▪▪▪▪▪▪▪▪▪▪▪▪▪▪▪▪▪□▪▪▪▪▪▪▪▪▪▪▪▪▪□□□□▪▪▪▪▪▪▪	377777377760771	3 (0.38)	66.7	T4	Clustered
3014*	▪▪□▪▪▪▪▪▪▪▪▪▪▪▪▪▪▪▪▪□□□□▪▪▪▪▪▪□□□□□□▪▪▪▪▪▪▪	677777607700771	1 (0.13)	50.0	Unknown	Unique
3015*	□□□▪▪▪▪▪▪▪▪▪▪▪▪▪▪□▪▪▪▪▪▪▪▪▪▪▪▪▪▪□□□□▪▪▪□□□▪	077776777760701	2 (0.25)	100.0	X1	Clustered
3016*	▪▪□□□□□□□□□□□□□□□□□□□□□□□□□▪□▪▪▪□□□□▪▪▪▪▪▪▪	600000000560771	1 (0.13)	50.0	Unknown	Unique
3017*	▪▪▪▪▪▪▪▪▪▪▪▪▪▪▪▪▪▪▪▪▪▪▪▪▪▪▪□□▪□▪□□□□▪▪▪▪▪▪▪	777777777120771	3 (0.38)	100.0	H3	Clustered
3089*	□▪▪□▪▪▪▪▪▪▪▪▪▪▪▪▪▪▪▪▪▪▪▪▪▪▪▪▪▪▪▪□□□□▪▪▪▪▪▪▪	337777777760771	2 (0.25)	66.67	T1	Clustered
3168*	▪▪▪□▪▪▪▪▪▪▪▪▪▪▪▪▪▪▪▪▪▪▪▪▪▪▪▪▪▪□▪□□□□▪▪▪□▪▪▪	737777777720731	1 (0.13)	50	H3	Unique
3431*	▪▪▪▪▪▪▪▪▪▪▪▪▪▪▪▪▪□▪▪▪▪▪▪▪□▪▪▪▪□▪□□□□▪▪▪▪▪▪▪	777776775720771	2 (0.25)	100	H3	Clustered
3432*	▪▪▪□□□□□□□□□□□□□▪▪▪▪□□□□▪▪▪▪▪▪▪▪□□□□▪▪▪▪▪▪▪	700003607760771	2 (0.25)	100	LAM3	Clustered
3433*	▪▪▪▪▪▪▪▪□□□▪▪▪▪▪▪▪▪▪□□□□▪▪▪▪□□▪▪□□□□▪▪▪▪▪▪▪	776177607460771	2 (0.25)	66.67	LAM3	Clustered

* A total of 100/122 SITs (n = 718) matched a preexisting shared-type in the database, whereas 22/122 SITs (n = 49 isolates) were newly created either within the present study or after a match with an orphan in the database. A total of 66 SITs containing 711 isolates were clustered within this study (2 to 130 isolates per cluster), while 56 SITs contained a unique strain within this study.

Note that SITs followed by an asterisk indicates “newly created shared-type” (n = 22 containing 49 isolates) due to 2 or more strains belonging to an identical new pattern within this study or after a match with an orphan in the database. SIT designations followed by number of strains: 2961* this study (n = 2); 3000* this study (n = 3); 3001* this study (n = 8) and USA (n = 1); 3004* this study (n = 2); 3005* this study (n = 2); 3006* this study (n = 2) and South Africa (n = 1); 3007* this study (n = 1) and USA (n = 1); 3008* this study (n = 1) and USA (n = 1); 3009* this study (n = 2); 3010* this study (n = 1) and USA (n = 1); 3011* this study (n = 4); 3012* this study (n = 2); 3013* this study (n = 3) and USA (n = 1); 3014* this study (n = 1) and Argentina (n = 1); 3015* this study (n = 2); 3016* this study (n = 1) and Panama (n = 1); 3017* this study (n = 3); 3089* this study (n = 2) and Mexico (n = 1); 3168* this study n = 1, Sweden (n = 1); 3431* this study (n = 2); 3432* this study (n = 2); 3433* this study (n = 2), BRA (n = 1).

** Lineage designations according to SITVIT2 using revised SpolDB4 rules; “Unknown” designates patterns with signatures that do not belong to any of the major clades described in the database.

*** Clustered strains correspond to a similar spoligotype pattern shared by 2 or more strains “within this study”; as opposed to unique strains harboring a spoligotype pattern that does not match with another strain from this study. Unique strains matching a preexisting pattern in the SITVIT2 database are classified as SITs, whereas in case of no match, they are designated as “orphan” (see Table 1).

498 strains (62.7% of all 794) were clustered in the nine most common spoligotypes; 16.4% SIT 50 (clade H3), 12.3% SIT 53 (clade T1), 8.3% SIT 33 (LAM3), 7.4% SIT 42 (LAM9), 5.5% SIT 1 (Beijing), 3.9% SIT 47 (H1), 3.0% SIT 222 (clade unknown), 3.0% SIT1355 (LAM), and 2.8% SIT 92 (X3) (table 3).

**Table 3:** Description of clusters composed of predominant shared types (defined as SITs representing >2% strains, n = 16) in our study and their worldwide distribution in the SITVIT2 database.

SIT (Clade) Octal NumberSpoligotype Description	Number (%) in study	% in study vs. SITVIT2	Distribution in Regions with ≥3% of a given SITs *	Distribution in Countries with ≥3% of a given SITs **
50 (H3) 777777777720771▪▪▪▪▪▪▪▪▪▪▪▪▪▪▪▪▪▪▪▪▪▪▪▪▪▪▪▪▪▪□▪□□□□▪▪▪▪▪▪▪	130 (16.37)	4.17	AMER-N 19.94, AMER-S 18.5, EURO-W 13.78, EURO-S 12.56, EURO-E 5.78, EURO-N 4.66, AFRI-N 4.62, AFRI-S 4.4, CARI 3.76, ASIA-W 3.02	USA 19.08, BRA 7.68, AUT 6.62, ITA 5.91, ESP 5.91, PER 4.46, ZAF 4.4, CZE 3.98, SWE 3.08
53 (T1) 777777777760771▪▪▪▪▪▪▪▪▪▪▪▪▪▪▪▪▪▪▪▪▪▪▪▪▪▪▪▪▪▪▪▪□□□□▪▪▪▪▪▪▪	98 (12.34)	1.74	AMER-N 18.23, AMER-S 13.2, EURO-W 11.32, EURO-S 10.5, ASIA-W 7.61, EURO-N 5.95, AFRI-S 5.54, AFRI-E 5.03, ASIA-E 4.73, AFRI-N 3.93	USA 14.73, ITA 5.95, BRA 5.72, ZAF 5.42, TUR 3.87, AUT 3.82, CHN 3.45, MEX 3.18
33 (LAM3) 776177607760771▪▪▪▪▪▪▪▪□□□▪▪▪▪▪▪▪▪▪□□□□▪▪▪▪▪▪▪▪□□□□▪▪▪▪▪▪▪	66 (8.31)	6.05	AFRI-S 29.79, AMER-S 25.76, AMER-N 15.03, EURO-S 12.65, EURO-W 5.04, AMER-C 4.49	ZAF 29.79, USA 14.57, BRA 12.1, ESP 8.16, PER 6.23, ARG 5.23, HND 3.94, ITA 3.85
42 (LAM9) 777777607760771▪▪▪▪▪▪▪▪▪▪▪▪▪▪▪▪▪▪▪▪□□□□▪▪▪▪▪▪▪▪□□□□▪▪▪▪▪▪▪	59 (7.43)	1.92	AMER-S 31.52, AMER-N 14.87, EURO-S 10.9, AFRI-N 9.34, EURO-W 6.25, EURO-N 4.07, AFRI-E 3.87, AFRI-S 3.45	BRA 13.05, USA 12.92, COL 8.3, MAR 7.68, ITA 5.69, ESP 3.64, VEN 3.61, ZAF 3.45
1 (Beijing) 000000000003771□□□□□□□□□□□□□□□□□□□□□□□□□□□□□□□□□□▪▪▪▪▪▪▪▪▪	44 (5.54)	0.46	ASIA-E 34.21, AMER-N 20.99, ASIA-SE 9.48, AFRI-S 8.63, ASIA-N 7.22, ASIA-S 4.67, EURO-N 3.23	USA 20.65, CHN 19.77, JPN 12.0, ZAF 8.63, RUS 7.22, VNM 4.06
47 (H1) 777777774020771▪▪▪▪▪▪▪▪▪▪▪▪▪▪▪▪▪▪▪▪▪▪▪▪▪□□□□□□▪□□□□▪▪▪▪▪▪▪	31 (3.9)	2.24	AMER-N 19.32, EURO-W 17.81, EURO-S 15.21, AMER-S 12.76, EURO-E 7.43, EURO-N 7.14, AFRI-N 4.11, ASIA-W 3.89	USA 17.16, ITA 9.3, AUT 9.08, BRA 7.93, CZE 4.25, ESP 4.04, SWE 3.82, MAR 3.17
222 (Unknown) 777774077560771▪▪▪▪▪▪▪▪▪▪▪▪▪▪▪▪□□□□□▪▪▪▪▪▪▪□▪▪▪□□□□▪▪▪▪▪▪▪	24 (3.02)	46.15	AMER-S 51.92, AMER-N 34.62, EURO-S 9.62, EURO-N 3.85	PER 50.0, USA 32.69, ESP 5.77, SWE 3.85, ITA 3.85
1355 (LAM) 777777407560731▪▪▪▪▪▪▪▪▪▪▪▪▪▪▪▪▪▪▪□□□□□▪▪▪▪□▪▪▪□□□□▪▪▪□▪▪▪	24 (3.02)	50	AMER-S 56.25, EURO-S 27.08, AMER-N 14.58	PER 54.17, ITA 20.83, USA 14.58, ESP 6.25
91 (X3_var) 700036777760771▪▪▪□□□□□□□□□□▪▪▪▪□▪▪▪▪▪▪▪▪▪▪▪▪▪▪□□□□▪▪▪▪▪▪▪	22 (2.77)	8.76	AMER-N 51.0, AMER-S 21.91, CARI 13.94, EURO-S 6.38, EURO-N 4.38	USA 47.01, HTI 11.55, PER 9.56, ESP 6.38, COL 4.78, GUF 3.98, CAN 3.19

* Worldwide distribution is reported for regions with more than 3% of a given SITs as compared to their total number in the SITVIT2 database. The definition of macro-geographical regions and sub-regions (http://unstats.un.org/unsd/methods/m49/m49regin.htm) is according to the United Nations; Regions: AFRI (Africa), AMER (Americas), ASIA (Asia), EURO (Europe), and OCE (Oceania), subdivided in: E (Eastern), M (Middle), C (Central), N (Northern), S (Southern), SE (South-Eastern), and W (Western). Furthermore, CARIB (Caribbean) belongs to Americas, while Oceania is subdivided in 4 sub-regions, AUST (Australasia), MEL (Melanesia), MIC (Micronesia), and POLY (Polynesia). Note that in our classification scheme, Russia has been attributed a new sub-region by itself (Northern Asia) instead of including it among rest of the Eastern Europe. It reflects its geographical localization as well as due to the similarity of specific TB genotypes circulating in Russia (a majority of Beijing genotypes) with those prevalent in Central, Eastern and South-Eastern Asia.

** The 3 letter country codes are according to http://en.wikipedia.org/wiki/ISO_3166-1_alpha-3; countrywide distribution is only shown for SITs with ≥3% of a given SITs as compared to their total number in the SITVIT2 database.

**Figure 1:**
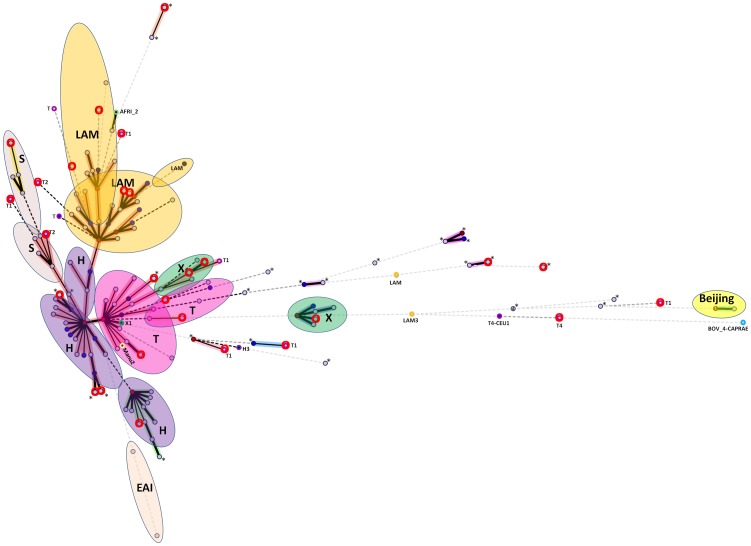
A minimum spanning tree (MST) illustrating evolutionary relationships between the Peruvian spoligotypes (n = 794). The phylogenetic tree connects each genotype based on degree of changes required to go from one allele to another. The structure of the tree is represented by branches (continuous vs. dotted lines) and circles representing each individual pattern. Note that the length of the branches represents the distance between patterns while the complexity of the lines (continuous, black dotted and gray dotted) denotes the number of allele/spacer changes between two patterns: solid lines, 1 or 2 changes (thicker ones indicate a single change, while the thinner ones indicate 2 changes); dotted lines, three or more changes (black dotted for 3, and grey dotted for 4 or more changes). The color of the circles is proportional to the number of clinical isolates in our study, illustrating unique isolates (sky blue) versus clustered isolates (Blue, 2–5 strains; dark blue, 6–9 strains; Bordeaux, 10–19 strains; Red, 20 and more). Note that orphan patterns are circled with the letter “o” in red. Patterns marked by an asterisk (*) indicate a strain with an unknown signature (unclassified).

**Figure 2:**
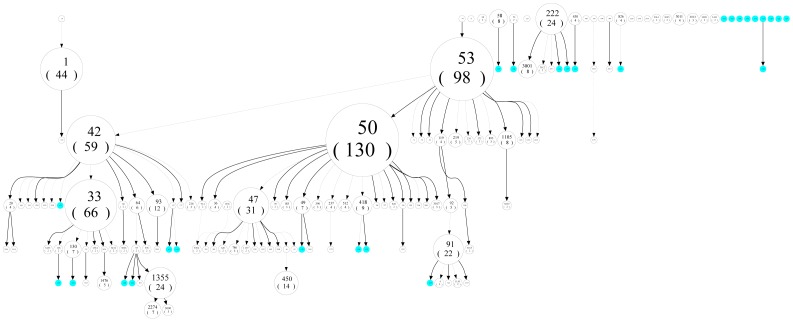
Discrete spoligotypes relationships for all isolates (n = 794) presented through spoligoforest tree drawn as a “hierarchical layout” using the SpolTools software (available through http://www.emi.unsw.edu.au/spolTools **; Reyes et al. 2008** [**11**] **).** Each spoligotype pattern from the study is represented by a node with area size being proportional to the total number of isolates with that specific pattern. Changes (loss of spacers) are represented by directed edges between nodes, with the arrowheads pointing to descendant spoligotypes. In this representation, the heuristic used selects a single inbound edge with a maximum weight using a Zipf model. Solid black lines link patterns that are very similar, i.e., loss of one spacer only (maximum weight being 1.0), while dashed lines represent links of weight comprised between 0.5 and 1, and dotted lines a weight less than 0.5. Orphan isolates, indicated in cyan, are isolated strains without interconnections with the other strains. This presentation illustrates for example the parental links for PGG2/3 strains such as SIT53 and SIT42, showing how SIT53 may be considered as the precursor of all other modern PGG2/3 patterns. SIT53 leads to SIT50/H3 by the loss of spacer 31, and it leads to SIT42 by the loss of four spacers (spacers 21–24), which in turn leads to SIT1355/LAM via SIT64/LAM6 then SIT95/LAM6. Through other spacer deletions, SIT53 leads to SIT91/X3 via SIT119/X1 and SIT92/X3. Lastly, SIT222/Unknown has no parental SITs in our study.

The phylogenetic relationships between strains are illustrated in minimum spanning trees (Figure 1) which demonstrate that PGG2/3 (H, LAM, T, X, S) strains are highly predominant (representing 83.8% of all strains), most notably H (n = 228, 28.7%), LAM (n = 225, 28.3%) and T (n = 161, 20.3%). (figure S1 includes SIT numbers which can be seen by zooming in on pdf file). The spoligoforests shown in Figure 2 (and figure S2) highlight (regardless of layout technique) that SIT50 (H3) is the largest node (n = 130), followed by SIT53 (T1, n = 98), SIT33 (LAM3, n = 66), SIT42 (LAM9, n = 59) and SIT1 (Beijing, n = 44).

### Strain clade associations

There was no predominant spoligotype associated with MDR amongst TB patients without HIV co-infection (table 4) - the odds of MDR were highest in those with disease caused by the LAM9 spoligotype SIT 42 though this was not statistically significant. Amongst patients with HIV co-infection this spoligotype was associated with by far the highest odds of MDR (87.5% of HIV patients with SIT42 disease had MDR); the T1 spoligotype SIT 53 was also associated with a increased odds of MDR, though only amongst patients with HIV (60.0% of HIV patients with SIT53 disease had MDR compared to 14.0% of HIV uninfected patients).

**Table 4:** Strain spoligotype (SIT) frequency by patient HIV status and strain MDR status.

	HIV negative		HIV positive		SIT total number of isolates^1^ (% MDR^2^)
SIT (of clade)	MDR	Non-MDR	MDR	Non-MDR	
**50 (H3)**	7	96	3	22	128 (7.8%)
**53 (T1)**	8	49	24	16	97 (33.0%)
**33 (LAM3)**	1	53	0	11	65 (1.54%)
**42 (LAM9)**	4	15	35	5	59 (66.1%)
**1 (Beijing)**	3	32	1	8	44 (9.1%)
**47 (H1)**	2	24	0	5	31 (6.5%)
**22 (unknown)**	2	16	2	4	24 (16.7%)
**1355 (LAM)**	1	12	4	6	23 (21.7%)
**91 (X3)**	1	14	1	6	22 (9.1%)
**others**	24	183	12	70	289 (12.5%)
**total**	53	494	82	153	782 (17.3%)

1 12 of 794 isolates lacked either MDR or HIV status data, leaving 782 here as the denominator.

2 Percentage of SIT-specific isolates with MDR.

HIV infection was strongly associated with MDRTB in this analysis. This association was significantly reduced (though incompletely mitigated) after adjustment for confounders, an effect largely mediated by inclusion of SIT42 and SIT53 (Table 5). Indeed even after adjustment for HIV and other covariates SIT42 and SIT53 were independently associated with MDRTB, though not with either isoniazid or rifampicin resistance. On univariate analysis male gender was associated with MDRTB but this effect was entirely driven by the increased HIV prevalence in males and disappeared after adjustment in the multivariate model. Neither study site nor year of sample collection were associated with drug resistance in the multivariate model.

**Table 5:** Associations with drug resistance.

	Isoniazid monoresistance		Rifampicin monoresistance		MDR	
	UnadjustedOR (95% CI)	Adjusted^1^OR (95% CI)	UnadjustedOR (95% CI)	Adjusted^1^OR (95% CI)	UnadjustedOR (95% CI)	Adjusted^1^OR (95% CI)
**HIV**	1.05 (0.61–1.81)	1.05 (0.59–1.87)	1.63 (0.83–3.20)	1.70 (0.83–3.49)	**5.00 (3.38–7.38)**	**3.42 (2.21–5.29)**
**Male gender**	1.18 (0.69–2.01)	1.20 (0.69–2.08)	1.21 (0.60–2.44)	1.07 (0.51–2.24)	**1.51 (1.01–2.27)**	1.13 (0.70–1.80)
**Year**	1.04 (0.91–1.18)	1.04 (0.91–1.19)	**1.20 (1.01–1.43)**	1.18 (0.98–1.41)	0.99 (0.90–1.09)	1.03 (0.92–1.16)
**Clustered SIT**	0.92 (0.55–1.54)	0.94 (0.54–1.64)	**0.38 (0.19–0.75)**	**0.43 (0.20–0.91)**	**1.77 (1.17–2.67)**	0.69 (0.41–1.18)
**SIT42 (LAM9)**	0.86 (0.64–1.16)	0.87 (0.63–1.19)	0.76 (0.46–1.25)	0.81 (0.48–1.37)	**1.89 (1.63–2.18)**	**1.95 (1.64–2.32)**
**SIT53 (T1)**	1.12 (0.78–1.59)	1.12 (0.76–1.65)	0.78 (0.43–1.42)	0.96 (0.50–1.85)	**1.67 (1.32–2.11)**	**2.16 (1.60–2.91)**

OR = odds ratio; 95% CI = 95% confidence interval; clustered SIT = in 9 most common SITs (accounting for 63% of all strains); bold type indicates statistically significant associations.

1 all adjusted ORs incorporate SIT42 and SIT53 and clustered SIT variable and into model.

## Discussion

In this report of strain diversity from Peru covering the period 1999–2005 3.4% of spoligotypes observed were from novel, orphan strains. The nine most frequently observed spoligotypes (out of 149 observed) accounted for over 60% of all disease and the eight of these also featured amongst the nine most frequently observed in a previous study in north Lima [2]; 5.5% were SIT1/Beijing family. Strains belonging to Haarlem, T, LAM and Beijing families predominated, and drug-resistance was not shown to be associated with any specific family, including Beijing, findings consistent with the single previous report from Peru [2].

With the exception of the Beijing family strains, recently examined in greater detail [10] very few PGG1 strains (AFRI, BOV, EAI, Manu) were found in this study (n = 9, 1.1%). One may notice that these PGG1 strains are not located at central positions on the trees (spoligoforests and MST). Instead, they mostly occupied terminal leaves of the trees (in the MST), or were isolated with few or no connections with other strains in the spoligoforests.

However, PGG2/3 group (n = 665, 83.8%) strains which are predominant in the study occupied a more visible and central position on the trees. Spoligoforest trees have been used to highlight the predominance of some specific well known shared types (SIT). These trees can also give us an overview on the parental links that probably exist between strains belonging to different lineages. For example, one may notice on the top left of the hierarchical layout figure (2A), that SIT19/EAI2-Manila may lead to SIT1/Beijing, through loss of many spacers.

The MST very well shows the similarity (or the distance) between each strain, and clearly defines the major evolution of the MTB lineages present in the study. For example, one can notice that in Figure 1, the Beijing family group is very far from the strains present in the central nodes of strains belonging to the PGG2/3 group (Euro American). At the very bottom of the MST, we can note the presence of the only two strains belonging to EAI lineage (SIT19/EAI2-Manila and SIT11/EAI3-IND).

The spoligoforest tree demonstrates that most of the orphans belong to the modern PGG2/3 group (H, T, LAM, T, X, S). The orphan strains are mostly located at terminal positions on the trees or are located in the top right layer of the hierarchical layout as isolated strains without interconnections with the other strains. Indeed, none of the orphans explicitly belonged to the PGG1 group (considering that some orphans have an unknown lineage).

Given the celebrated performance of the Peruvian National Tuberculosis Control Programme in demonstrating the effectiveness of DOTS in bringing about a reduction in TB incidence [3], the emergence of MDRTB in Peru as a major threat might be viewed as surprising [11]; conventional wisdom suggests MDR is driven by weak healthcare systems. We were therefore interested in exploring whether other factors, such as strain-specific biological propensity for resistance, might be relevant. In unselected community-based TB patients (largely HIV-negative) there was no association observed between drug resistance and specific spoligotypes. However amongst patients with HIV recruited from a hospital setting MDR was particularly frequently seen amongst the SIT42 and SIT53 strains. After multivariate analysis to control for the effects of HIV infection, gender and year the effect size increased; given the lack of such an association in the community one hypothesis to explain this would be that this is highly suggestive of a prolonged nosocomial clonal outbreak with strains of these two spoligotypes. The alternative hypothesis of a biological predisposition of these specific strains to acquire drug resistance-conferring mutations is much less likely given the absence of an association with isoniazid or rifampicin mono-resistance. It is noteworthy that the association of HIV with MDR, though diminished after adjustment for SIT42 and SIT53, remained significant indicating that an outbreak with strains from these two spoligotypes is insufficient to explain the whole HIV-MDR association. We cannot exclude the possibility of residual confounding as the explanation for this apparent association.

There are acknowledged limitations of the data presented here. Most importantly our sampling strategy was opportunistic, making use of a strain bank derived from several studies with different designs so the study populations differed and though all relevant subgroups (community and hospital based, HIV infected and uninfected) were included the sample could not be considered representative. There are advantages in having a strain bank which is delinked from patient identifiers but the drawback is that only limited clinical data is available and returning to clinical notes for further detail is not possible – it would have been interesting to differentiate between new and retreatment cases and to investigate patient outcomes by strain, for example. Finally, because the samples were all from studies in adults we were unable to describe the strains causing paediatric disease thus missing an opportunity to clearly identify currently/recently circulating strains, and because all strains were from pulmonary TB patients we were unable to investigate whether extrapulmonary disease phenotype was associated with any particular strain in Peru as has been suggested elsewhere [12].

There are important strengths in our strain bank: (1) each sample evaluated here is a single strain from a unique patient – though serial strains are available in the bank, for this analysis we were careful to only examine one strain per patient, (2) availability of drug susceptibility data and HIV status for every strain enabled the analysis we report here with very few missing values, in contrast to an earlier report for which only 70% of strains had drug susceptibility data and HIV status was not reported [2], (3) the spread of strains includes diverse but well characterized patient demographic groups which are also geographically spread across metropolitan Lima (home to one third of the population of Peru and more than 75% of the incident TB), (4) the collection reported here span a time period of 6 years (indeed the bank continues to accumulate strains to the present day, extending the collection to more than 13 years) enabling investigation of temporal trends (none were found here).

In summary, we report the strain distribution of *M tuberculosis* isolates in Lima, Peru, highlight a significant proportion of novel spoligotypes, and hypothesize a prolonged, clonal, hospital-based outbreak of MDR disease amongst HIV patients but no evidence to support a hypothesis of strain-specific propensity for the acquisition of resistance-conferring mutations.

## Supporting Information

Figure S1 **MST from manuscript** **Figure 1** **presented in PDF format which (through zooming in) enables reading of SIT labels.**
(PDF)

Figure S2 **Discrete spoligotypes relationships for all isolates (n = 794) presented through a Fruchterman Reingold spoligoforest tree drawn using the SpolTools software (available through** http://www.emi.unsw.edu.au/spolTools **; Reyes et al. 2008** [**11**] **).** Each spoligotype pattern from the study is represented by a node with area size being proportional to the total number of isolates with that specific pattern. Changes (loss of spacers) are represented by directed edges between nodes, with the arrowheads pointing to descendant spoligotypes. In this representation, the heuristic used selects a single inbound edge with a maximum weight using a Zipf model. Solid black lines link patterns that are very similar, i.e., loss of one spacer only (maximum weight being 1.0), while dashed lines represent links of weight comprised between 0.5 and 1, and dotted lines a weight less than 0.5. Orphan isolates, indicated in cyan, appear at terminal positions on the tree, as isolated strains without interconnections with the other strains.
(PDF)

Table S1 Detailed genotyping and drug-resistance data and demographic information on *M. tuberculosis* strains (n = 794) isolated from adults with pulmonary tuberculosis in Lima, Peru.
(PDF)

Table S2 A comparison of the proportion of the most predominant SITs found in Peru as compared to neighbouring countries (Brazil, Colombia) and regions (Central America and Caribbean), recorded in the SITVIT2 database as consulted on 9 April 2013.
(PDF)
