# Supplementary figures and images for: Genetic Diversity of Mycobacterium tuberculosis in Peru and Exploration of Phylogenetic Associations with Drug Resistance
Source: PLoS One. 2013 Jun 24;8(6):e65873. doi: 10.1371/journal.pone.0065873 (PMC3691179; doi:10.1371/journal.pone.0065873)

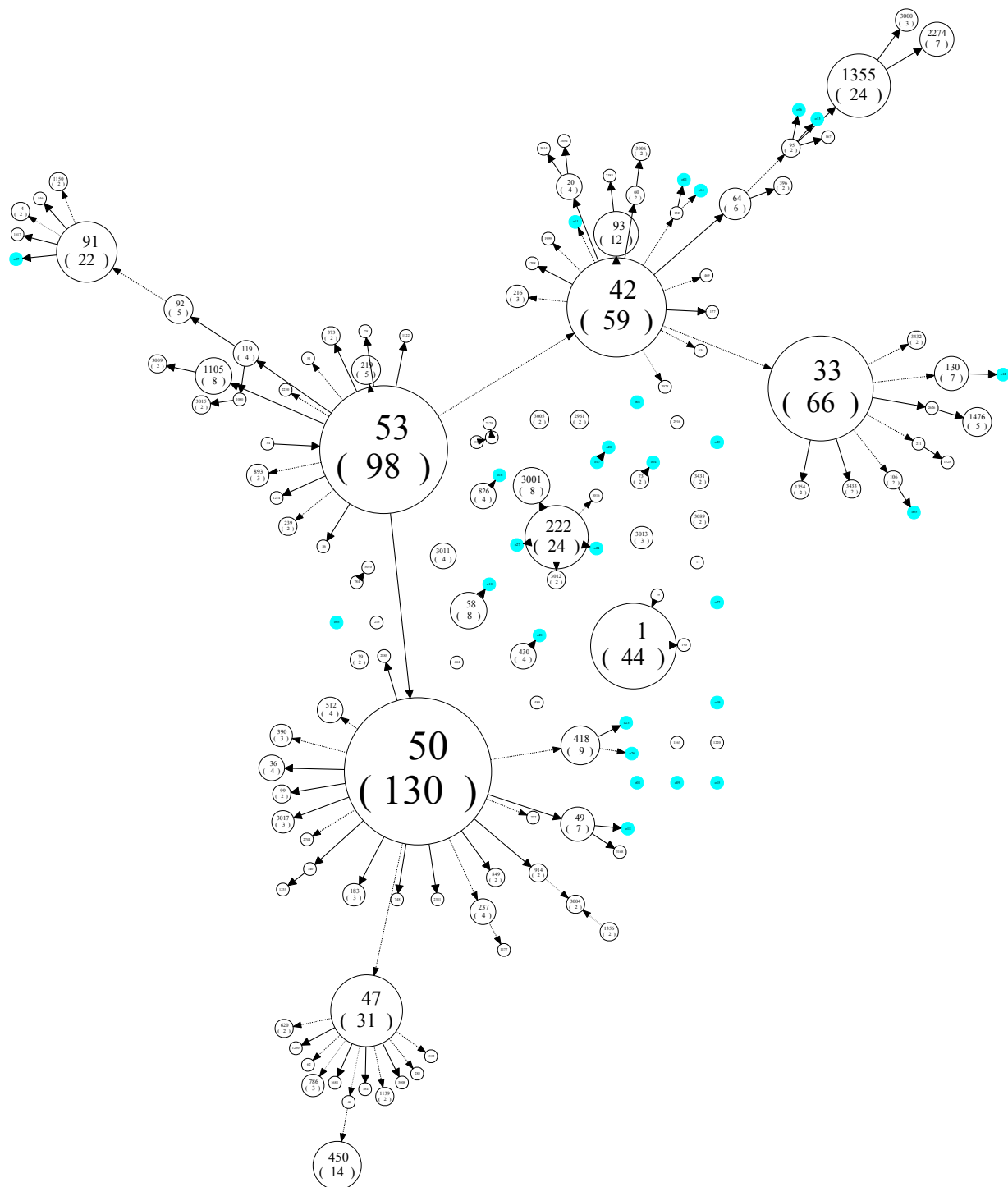

Supplement: Figure S2 — Discrete spoligotypes relationships for all isolates (n = 794) presented through a Fruchterman Reingold spoligoforest tree drawn using the SpolTools software (available through http://www.emi.unsw.edu.au/spolTools ; Reyes et al. 2008 [11] ). Each spoligotype pattern from the study is represented by a node with area size being proportional to the total number of isolates with that specific pattern. Changes (loss of spacers) are represented by directed edges between nodes, with the arrowheads pointing to descendant spoligotypes. In this representation, the heuristic used selects a single inbound edge with a maximum weight using a Zipf model. Solid black lines link patterns that are very similar, i.e., loss of one spacer only (maximum weight being 1.0), while dashed lines represent links of weight comprised between 0.5 and 1, and dotted lines a weight less than 0.5. Orphan isolates, indicated in cyan, appear at terminal positions on the tree, as isolated strains without interconnections with the other strains. (PDF) [file pone.0065873.s002.pdf]
