# Supplementary material for: Genetic Diversity of Mycobacterium tuberculosis in Peru and Exploration of Phylogenetic Associations with Drug Resistance
Source: PLoS One. 2013 Jun 24;8(6):e65873. doi: 10.1371/journal.pone.0065873 (PMC3691179; doi:10.1371/journal.pone.0065873)
Supplement: Table S1 — Detailed genotyping and drug-resistance data and demographic information on M. tuberculosis strains (n = 794) isolated from adults with pulmonary tuberculosis in Lima, Peru. (PDF) [file pone.0065873.s003.pdf]

<u>Supplemental Table S1</u>: Detailed genotyping and drug-resistance data and demographic information on *M. tuberculosis* strains (n=794) isolated from adults with pulmonary tuberculosis in Lima, Peru.

IsoNumber	Year	Strain	Nb	Spoligotype Description	Octal code	Clade*	SIT**	Drug-R Code***	Sex/ Age	Drug-R	HIV Serology
PER01200330004	2003	0004	1	□□□□□□□□□□□□□□□□□□□□□□□□□□□□□□□□□□■■■■■■■■■	000000000003771	Beijing	1	3	F/29	RIF resist	
PER01200410030	2004	0030	1	□□□□□□□□□□□□□□□□□□□□□□□□□□□□□□□□□□■■■■■■■■■	000000000003771	Beijing	1	1	M/38		
PER01200410069	2004	0069	1	□□□□□□□□□□□□□□□□□□□□□□□□□□□□□□□□□□■■■■■■■■■	000000000003771	Beijing	1	1	M/23		
PER01200310070	2003	0070	1	□□□□□□□□□□□□□□□□□□□□□□□□□□□□□□□□□□■■■■■■■■■	000000000003771	Beijing	1	1	M/19		
PER01200320072	2003	0072	1	□□□□□□□□□□□□□□□□□□□□□□□□□□□□□□□□□□■■■■■■■■■	000000000003771	Beijing	1	2	F/48		
PER01200310086	2003	0086	1	□□□□□□□□□□□□□□□□□□□□□□□□□□□□□□□□□□■■■■■■■■■	000000000003771	Beijing	1	1	M/34		
PER01200310090	2003	0090	1	□□□□□□□□□□□□□□□□□□□□□□□□□□□□□□□□□□■■■■■■■■■	000000000003771	Beijing	1	1	M/19		
PER01200310125	2003	0125	1	□□□□□□□□□□□□□□□□□□□□□□□□□□□□□□□□□□■■■■■■■■■	000000000003771	Beijing	1	1	F/37		
PER01200310126	2003	0126	1	□□□□□□□□□□□□□□□□□□□□□□□□□□□□□□□□□□■■■■■■■■■	000000000003771	Beijing	1	1	F/43		
PER01200310143	2003	0143	1	□□□□□□□□□□□□□□□□□□□□□□□□□□□□□□□□□□■■■■■■■■■	000000000003771	Beijing	1	1	M/48		
PER01200420171	2004	0171	1	□□□□□□□□□□□□□□□□□□□□□□□□□□□□□□□□□□■■■■■■■■■	000000000003771	Beijing	1	2	F/23		
PER01199930260	1999	0260	1	□□□□□□□□□□□□□□□□□□□□□□□□□□□□□□□□□□■■■■■■■■■	000000000003771	Beijing	1	3	M/47	RIF resist	HIV+
PER01200030277	2000	0277	1	□□□□□□□□□□□□□□□□□□□□□□□□□□□□□□□□□□■■■■■■■■■	000000000003771	Beijing	1	3	M/32	INH resist	HIV+
PER01200110308	2001	0308	1	□□□□□□□□□□□□□□□□□□□□□□□□□□□□□□□□□□■■■■■■■■■	000000000003771	Beijing	1	1	M/26		HIV+
PER01200110329	2001	0329	1	□□□□□□□□□□□□□□□□□□□□□□□□□□□□□□□□□□■■■■■■■■■	000000000003771	Beijing	1	1	F/30		HIV+
PER01199930342	1999	0342	1	□□□□□□□□□□□□□□□□□□□□□□□□□□□□□□□□□□■■■■■■■■■	000000000003771	Beijing	1	3	M/0	INH resist	
PER01199910343	1999	0343	1	□□□□□□□□□□□□□□□□□□□□□□□□□□□□□□□□□□■■■■■■■■■	000000000003771	Beijing	1	1	M/17		
PER01199910351	1999	0351	1	□□□□□□□□□□□□□□□□□□□□□□□□□□□□□□□□□□■■■■■■■■■	000000000003771	Beijing	1	1	F/0		
PER01199910360	1999	0360	1	□□□□□□□□□□□□□□□□□□□□□□□□□□□□□□□□□□■■■■■■■■■	000000000003771	Beijing	1	1	M/0		
PER01199910368	1999	0368	1	□□□□□□□□□□□□□□□□□□□□□□□□□□□□□□□□□□■■■■■■■■■	000000000003771	Beijing	1	1	F/0		
PER01199910383	1999	0383	1	□□□□□□□□□□□□□□□□□□□□□□□□□□□□□□□□□□■■■■■■■■■	000000000003771	Beijing	1	1	F/0		
PER01199910395	1999	0395	1	□□□□□□□□□□□□□□□□□□□□□□□□□□□□□□□□□□■■■■■■■■■	000000000003771	Beijing	1	1	F/0		
PER01199910424	1999	0424	1	□□□□□□□□□□□□□□□□□□□□□□□□□□□□□□□□□□■■■■■■■■■	000000000003771	Beijing	1	1	M/23		
PER01199930425	1999	0425	1	□□□□□□□□□□□□□□□□□□□□□□□□□□□□□□□□□□■■■■■■■■■	000000000003771	Beijing	1	3	M/0	RIF resist	
PER01199910431	1999	0431	1	□□□□□□□□□□□□□□□□□□□□□□□□□□□□□□□□□□■■■■■■■■■	000000000003771	Beijing	1	1	M/0		
PER01199910451	1999	0451	1	□□□□□□□□□□□□□□□□□□□□□□□□□□□□□□□□□□■■■■■■■■■	000000000003771	Beijing	1	1	F/66		
PER01200010492	2000	0492	1	□□□□□□□□□□□□□□□□□□□□□□□□□□□□□□□□□□■■■■■■■■■	000000000003771	Beijing	1	1	M/0		
PER01200010501	2000	0501	1	□□□□□□□□□□□□□□□□□□□□□□□□□□□□□□□□□□■■■■■■■■■	000000000003771	Beijing	1	1	M/0		
PER01200010522	2000	0522	1	□□□□□□□□□□□□□□□□□□□□□□□□□□□□□□□□□□■■■■■■■■■	000000000003771	Beijing	1	1	M/0		
PER01200010542	2000	0542	1	□□□□□□□□□□□□□□□□□□□□□□□□□□□□□□□□□□■■■■■■■■■	000000000003771	Beijing	1	1	M/23		
PER01200030559	2000	0559	1	□□□□□□□□□□□□□□□□□□□□□□□□□□□□□□□□□□■■■■■■■■■	000000000003771	Beijing	1	3	M/0	INH resist	
PER01200110589	2001	0589	1	□□□□□□□□□□□□□□□□□□□□□□□□□□□□□□□□□□■■■■■■■■■	000000000003771	Beijing	1	1	M/0		
PER01200130599	2001	0599	1	□□□□□□□□□□□□□□□□□□□□□□□□□□□□□□□□□□■■■■■■■■■	000000000003771	Beijing	1	3	F/0	INH resist	
PER01200110603	2001	0603	1	□□□□□□□□□□□□□□□□□□□□□□□□□□□□□□□□□□■■■■■■■■■	000000000003771	Beijing	1	1	M/0		
PER01200110605	2001	0605	1	□□□□□□□□□□□□□□□□□□□□□□□□□□□□□□□□□□■■■■■■■■■	000000000003771	Beijing	1	1	F/0		
PER01200110621	2001	0621	1	□□□□□□□□□□□□□□□□□□□□□□□□□□□□□□□□□□■■■■■■■■■	000000000003771	Beijing	1	1	M/0		
PER01200120641	2001	0641	1	□□□□□□□□□□□□□□□□□□□□□□□□□□□□□□□□□□■■■■■■■■■	000000000003771	Beijing	1	2	M/34		
PER01200110645	2001	0645	1	□□□□□□□□□□□□□□□□□□□□□□□□□□□□□□□□□□■■■■■■■■■	000000000003771	Beijing	1	1	M/19		
PER01200210658	2002	0658	1	□□□□□□□□□□□□□□□□□□□□□□□□□□□□□□□□□□■■■■■■■■■	000000000003771	Beijing	1	1	M/16		
PER01200220690	2002	0690	1	□□□□□□□□□□□□□□□□□□□□□□□□□□□□□□□□□□■■■■■■■■■	000000000003771	Beijing	1	2	M/44		HIV+
PER01200310759	2003	0759	1	□□□□□□□□□□□□□□□□□□□□□□□□□□□□□□□□□□■■■■■■■■■	000000000003771	Beijing	1	1	M/29		HIV+
PER01200410779	2004	0779	1	□□□□□□□□□□□□□□□□□□□□□□□□□□□□□□□□□□■■■■■■■■■	000000000003771	Beijing	1	1	M/26		HIV+
PER01200410783	2004	0783	1	□□□□□□□□□□□□□□□□□□□□□□□□□□□□□□□□□□■■■■■■■■■	000000000003771	Beijing	1	1	M/36		HIV+
PER01200410792	2004	0792	1	□□□□□□□□□□□□□□□□□□□□□□□□□□□□□□□□□□■■■■■■■■■	000000000003771	Beijing	1	1	F/38		HIV+

PER01200010506	2000	0506	1	□□□□□□□□□□□□□□□□□□□□□□□□■■■■■■■■□□□□■■■■■■■	000000007760771	Unknown	4	1	M/26		
PER01200010571	2000	0571	1	□□□□□□□□□□□□□□□□□□□□□□□□■■■■■■■■□□□□■■■■■■■	000000007760771	Unknown	4	1	M/0		
PER01199910420	1999	0420	1	■□□■■■■■■■■■■■■■■■■■■■■■■■■■□□□□■□■■□□□■■■■	477777777413071	EAI3-IND	11	1	F/0		
PER01200110635	2001	0635	1	■■□■■■■■■■■■■■■■■■■□□■■■■■■■□□□□■□■■■■■■■■■	677777477413771	EAI2-Manilla	19	1	M/23		
PER01200320007	2003	0007	1	■■□■■■■■■■■■■■■■■■■■□□□□■■■■■■■■□□□□■■■■■■■	677777607760771	LAM1	20	2	F/58		
PER01200530058	2005	0058	1	■■□■■■■■■■■■■■■■■■■■□□□□■■■■■■■■□□□□■■■■■■■	677777607760771	LAM1	20	3	M/18	INH resist	
PER01200030491	2000	0491	1	■■□■■■■■■■■■■■■■■■■■□□□□■■■■■■■■□□□□■■■■■■■	677777607760771	LAM1	20	3	F/33	INH resist	
PER01200010579	2000	0579	1	■■□■■■■■■■■■■■■■■■■■□□□□■■■■■■■■□□□□■■■■■■■	677777607760771	LAM1	20	1	M/0		
PER01200310014	2003	0014	1	■■■■■■■■□□□■■■■■■■■■□□□□■■■■■■■■□□□□■■■■■■■	776177607760771	LAM3	33	1	F/19		
PER01200310015	2003	0015	1	■■■■■■■■□□□■■■■■■■■■□□□□■■■■■■■■□□□□■■■■■■■	776177607760771	LAM3	33	1	M/59		
PER01200410027	2004	0027	1	■■■■■■■■□□□■■■■■■■■■□□□□■■■■■■■■□□□□■■■■■■■	776177607760771	LAM3	33	1	M/22		
PER01200510042	2005	0042	1	■■■■■■■■□□□■■■■■■■■■□□□□■■■■■■■■□□□□■■■■■■■	776177607760771	LAM3	33	1	F/33		
PER01200510046	2005	0046	1	■■■■■■■■□□□■■■■■■■■■□□□□■■■■■■■■□□□□■■■■■■■	776177607760771	LAM3	33	1	M/36		
PER01200510050	2005	0050	1	■■■■■■■■□□□■■■■■■■■■□□□□■■■■■■■■□□□□■■■■■■■	776177607760771	LAM3	33	1	F/34		
PER01200510053	2005	0053	1	■■■■■■■■□□□■■■■■■■■■□□□□■■■■■■■■□□□□■■■■■■■	776177607760771	LAM3	33	1	F/25		
PER01200310077	2003	0077	1	■■■■■■■■□□□■■■■■■■■■□□□□■■■■■■■■□□□□■■■■■■■	776177607760771	LAM3	33	1	F/37		
PER01200310093	2003	0093	1	■■■■■■■■□□□■■■■■■■■■□□□□■■■■■■■■□□□□■■■■■■■	776177607760771	LAM3	33	1	F/25		
PER01200310098	2003	0098	1	■■■■■■■■□□□■■■■■■■■■□□□□■■■■■■■■□□□□■■■■■■■	776177607760771	LAM3	33	1	M/26		
PER01200310104	2003	0104	1	■■■■■■■■□□□■■■■■■■■■□□□□■■■■■■■■□□□□■■■■■■■	776177607760771	LAM3	33	1	M/20		
PER01200310110	2003	0110	1	■■■■■■■■□□□■■■■■■■■■□□□□■■■■■■■■□□□□■■■■■■■	776177607760771	LAM3	33	1	M/24		
PER01200310111	2003	0111	1	■■■■■■■■□□□■■■■■■■■■□□□□■■■■■■■■□□□□■■■■■■■	776177607760771	LAM3	33	1	M/20		
PER01200310120	2003	0120	1	■■■■■■■■□□□■■■■■■■■■□□□□■■■■■■■■□□□□■■■■■■■	776177607760771	LAM3	33	1	M/18		
PER01200410166	2004	0166	1	■■■■■■■■□□□■■■■■■■■■□□□□■■■■■■■■□□□□■■■■■■■	776177607760771	LAM3	33	1	F/36		
PER01200410176	2004	0176	1	■■■■■■■■□□□■■■■■■■■■□□□□■■■■■■■■□□□□■■■■■■■	776177607760771	LAM3	33	1	M/40		
PER01200410183	2004	0183	1	■■■■■■■■□□□■■■■■■■■■□□□□■■■■■■■■□□□□■■■■■■■	776177607760771	LAM3	33	1	M/21		
PER01200410185	2004	0185	1	■■■■■■■■□□□■■■■■■■■■□□□□■■■■■■■■□□□□■■■■■■■	776177607760771	LAM3	33	1	F/18		
PER01200430212	2004	0212	1	■■■■■■■■□□□■■■■■■■■■□□□□■■■■■■■■□□□□■■■■■■■	776177607760771	LAM3	33	3	M/28	RIF resist	
PER01199910227	1999	0227	1	■■■■■■■■□□□■■■■■■■■■□□□□■■■■■■■■□□□□■■■■■■■	776177607760771	LAM3	33	1	M/28		HIV+
PER01199910238	1999	0238	1	■■■■■■■■□□□■■■■■■■■■□□□□■■■■■■■■□□□□■■■■■■■	776177607760771	LAM3	33	1	M/29		HIV+
PER01199910246	1999	0246	1	■■■■■■■■□□□■■■■■■■■■□□□□■■■■■■■■□□□□■■■■■■■	776177607760771	LAM3	33	1	M/23		HIV+
PER01200010292	2000	0292	1	■■■■■■■■□□□■■■■■■■■■□□□□■■■■■■■■□□□□■■■■■■■	776177607760771	LAM3	33	1	F/29		HIV+
PER01199910346	1999	0346	1	■■■■■■■■□□□■■■■■■■■■□□□□■■■■■■■■□□□□■■■■■■■	776177607760771	LAM3	33	1	M/20		
PER01199910347	1999	0347	1	■■■■■■■■□□□■■■■■■■■■□□□□■■■■■■■■□□□□■■■■■■■	776177607760771	LAM3	33	1	M/0		
PER01199910354	1999	0354	1	■■■■■■■■□□□■■■■■■■■■□□□□■■■■■■■■□□□□■■■■■■■	776177607760771	LAM3	33	1	M/0		
PER01199910358	1999	0358	1	■■■■■■■■□□□■■■■■■■■■□□□□■■■■■■■■□□□□■■■■■■■	776177607760771	LAM3	33	1	M/20		
PER01199910364	1999	0364	1	■■■■■■■■□□□■■■■■■■■■□□□□■■■■■■■■□□□□■■■■■■■	776177607760771	LAM3	33	1	M/0		
PER01199910371	1999	0371	1	■■■■■■■■□□□■■■■■■■■■□□□□■■■■■■■■□□□□■■■■■■■	776177607760771	LAM3	33	1	F/32		
PER01199910372	1999	0372	1	■■■■■■■■□□□■■■■■■■■■□□□□■■■■■■■■□□□□■■■■■■■	776177607760771	LAM3	33	1	F/31		
PER01199910373	1999	0373	1	■■■■■■■■□□□■■■■■■■■■□□□□■■■■■■■■□□□□■■■■■■■	776177607760771	LAM3	33	1	F/0		
PER01199910387	1999	0387	1	■■■■■■■■□□□■■■■■■■■■□□□□■■■■■■■■□□□□■■■■■■■	776177607760771	LAM3	33	1	M/27		
PER01199910408	1999	0408	1	■■■■■■■■□□□■■■■■■■■■□□□□■■■■■■■■□□□□■■■■■■■	776177607760771	LAM3	33	1	M/0		
PER01199910410	1999	0410	1	■■■■■■■■□□□■■■■■■■■■□□□□■■■■■■■■□□□□■■■■■■■	776177607760771	LAM3	33	1	F/0		
PER01199910422	1999	0422	1	■■■■■■■■□□□■■■■■■■■■□□□□■■■■■■■■□□□□■■■■■■■	776177607760771	LAM3	33	1	M/17		
PER01199910423	1999	0423	1	■■■■■■■■□□□■■■■■■■■■□□□□■■■■■■■■□□□□■■■■■■■	776177607760771	LAM3	33	1	M/0		
PER01199910463	1999	0463	1	■■■■■■■■□□□■■■■■■■■■□□□□■■■■■■■■□□□□■■■■■■■	776177607760771	LAM3	33	1	M/0		
PER01200010472	2000	0472	1	■■■■■■■■□□□■■■■■■■■■□□□□■■■■■■■■□□□□■■■■■■■	776177607760771	LAM3	33	1	F/0		
PER01200010497	2000	0497	1	■■■■■■■■□□□■■■■■■■■■□□□□■■■■■■■■□□□□■■■■■■■	776177607760771	LAM3	33	1	F/0		
PER01200010502	2000	0502	1	■■■■■■■■□□□■■■■■■■■■□□□□■■■■■■■■□□□□■■■■■■■	776177607760771	LAM3	33	1	M/0		
PER01200010507	2000	0507	1	■■■■■■■■□□□■■■■■■■■■□□□□■■■■■■■■□□□□■■■■■■■	776177607760771	LAM3	33	1	F/24		
PER01200010520	2000	0520	1	■■■■■■■■□□□■■■■■■■■■□□□□■■■■■■■■□□□□■■■■■■■	776177607760771	LAM3	33	1	M/0		
PER01200010526	2000	0526	1	■■■■■■■■□□□■■■■■■■■■□□□□■■■■■■■■□□□□■■■■■■■	776177607760771	LAM3	33	1	F/20		

PER01200010537	2000	0537	1	■■■■■■■■□□□■■■■■■■■■■□□□□■■■■■■■■□□□□■■■■■■■	776177607760771	LAM3	33	1	M/18		
PER01200010556	2000	0556	1	■■■■■■■■□□□■■■■■■■■■■□□□□■■■■■■■■□□□□■■■■■■■	776177607760771	LAM3	33	1	M/0		
PER01200010563	2000	0563	1	■■■■■■■■□□□■■■■■■■■■■□□□□■■■■■■■■□□□□■■■■■■■	776177607760771	LAM3	33	1	M/0		
PER01200010583	2000	0583	1	■■■■■■■■□□□■■■■■■■■■■□□□□■■■■■■■■□□□□■■■■■■■	776177607760771	LAM3	33	1	M/0		
PER01200110591	2001	0591	1	■■■■■■■■□□□■■■■■■■■■■□□□□■■■■■■■■□□□□■■■■■■■	776177607760771	LAM3	33	1	M/0		
PER01200110592	2001	0592	1	■■■■■■■■□□□■■■■■■■■■■□□□□■■■■■■■■□□□□■■■■■■■	776177607760771	LAM3	33	1	F/0		
PER01200110594	2001	0594	1	■■■■■■■■□□□■■■■■■■■■■□□□□■■■■■■■■□□□□■■■■■■■	776177607760771	LAM3	33	1	M/0		
PER01200110622	2001	0622	1	■■■■■■■■□□□■■■■■■■■■■□□□□■■■■■■■■□□□□■■■■■■■	776177607760771	LAM3	33	1	M/0		
PER01200110627	2001	0627	1	■■■■■■■■□□□■■■■■■■■■■□□□□■■■■■■■■□□□□■■■■■■■	776177607760771	LAM3	33	1	F/0		
PER01200120629	2001	0629	1	■■■■■■■■□□□■■■■■■■■■■□□□□■■■■■■■■□□□□■■■■■■■	776177607760771	LAM3	33	2	M/0		
PER01200110630	2001	0630	1	■■■■■■■■□□□■■■■■■■■■■□□□□■■■■■■■■□□□□■■■■■■■	776177607760771	LAM3	33	1	M/0		
PER01200110637	2001	0637	1	■■■■■■■■□□□■■■■■■■■■■□□□□■■■■■■■■□□□□■■■■■■■	776177607760771	LAM3	33	1	F/49		
PER01200110643	2001	0643	1	■■■■■■■■□□□■■■■■■■■■■□□□□■■■■■■■■□□□□■■■■■■■	776177607760771	LAM3	33	1	M/21		
PER01200210652	2002	0652	1	■■■■■■■■□□□■■■■■■■■■■□□□□■■■■■■■■□□□□■■■■■■■	776177607760771	LAM3	33	1	M/30		
PER01200210655	2002	0655	1	■■■■■■■■□□□■■■■■■■■■■□□□□■■■■■■■■□□□□■■■■■■■	776177607760771	LAM3	33	1	M/26		
PER01200210678	2002	0678	1	■■■■■■■■□□□■■■■■■■■■■□□□□■■■■■■■■□□□□■■■■■■■	776177607760771	LAM3	33	1	F/29		HIV+
PER01200210679	2002	0679	1	■■■■■■■■□□□■■■■■■■■■■□□□□■■■■■■■■□□□□■■■■■■■	776177607760771	LAM3	33	1	M/35		HIV+
PER01200210680	2002	0680	1	■■■■■■■■□□□■■■■■■■■■■□□□□■■■■■■■■□□□□■■■■■■■	776177607760771	LAM3	33	1	M/35		HIV+
PER01200310718	2003	0718	1	■■■■■■■■□□□■■■■■■■■■■□□□□■■■■■■■■□□□□■■■■■■■	776177607760771	LAM3	33	1	M/23		HIV+
PER01200330727	2003	0727	1	■■■■■■■■□□□■■■■■■■■■■□□□□■■■■■■■■□□□□■■■■■■■	776177607760771	LAM3	33	3	M/22	INH resist	HIV+
PER01200310748	2003	0748	1	■■■■■■■■□□□■■■■■■■■■■□□□□■■■■■■■■□□□□■■■■■■■	776177607760771	LAM3	33	1	M/25		HIV+
PER01200310757	2003	0757	1	■■■■■■■■□□□■■■■■■■■■■□□□□■■■■■■■■□□□□■■■■■■■	776177607760771	LAM3	33	1	F/27		HIV+
PER01200410793	2004	0793	1	■■■■■■■■□□□■■■■■■■■■■□□□□■■■■■■■■□□□□■■■■■■■	776177607760771	LAM3	33	1	F/38		HIV+
PER01200310101	2003	0101	1	■■■■■■■■■■■■□■■■■■■■■■■■■■■■■□■□□□□■■■■■■■	777737777720771	H3	36	1	M/27		
PER01200310734	2003	0734	1	■■■■■■■■■■■■□■■■■■■■■■■■■■■■■□■□□□□■■■■■■■	777737777720771	H3	36	1	M/32		HIV+
PER01200310751	2003	0751	1	■■■■■■■■■■■■□■■■■■■■■■■■■■■■■□■□□□□■■■■■■■	777737777720771	H3	36	1	M/30		HIV+
PER01200510800	2005	0800	1	■■■■■■■■■■■■□■■■■■■■■■■■■■■■■□■□□□□■■■■■■■	777737777720771	H3	36	1	M/29		HIV+
PER01199930385	1999	0385	1	■■■■■■■■■■■■■■■■■■□■■■□□■■■■■■■■□□□□■□□■■■■	777777347760471	T4-CEU1	39	3	M/0	INH resist	
PER01199910413	1999	0413	1	■■■■■■■■■■■■■■■■■■□■■■□□■■■■■■■■□□□□■□□■■■■	777777347760471	T4-CEU1	39	1	F/0		
PER01200410019	2004	0019	1	■■■■■■■■■■■■■■■■■■■■□□□□■■■■■■■■□□□□■■■■■■■	777777607760771	LAM9	42	1	M/43		
PER01200410024	2004	0024	1	■■■■■■■■■■■■■■■■■■■■□□□□■■■■■■■■□□□□■■■■■■■	777777607760771	LAM9	42	1	F/68		
PER01200410038	2004	0038	1	■■■■■■■■■■■■■■■■■■■■□□□□■■■■■■■■□□□□■■■■■■■	777777607760771	LAM9	42	1	M/25		
PER01200410039	2004	0039	1	■■■■■■■■■■■■■■■■■■■■□□□□■■■■■■■■□□□□■■■■■■■	777777607760771	LAM9	42	1	F/24		
PER01200510044	2005	0044	1	■■■■■■■■■■■■■■■■■■■■□□□□■■■■■■■■□□□□■■■■■■■	777777607760771	LAM9	42	1	M/46		
PER01200310094	2003	0094	1	■■■■■■■■■■■■■■■■■■■■□□□□■■■■■■■■□□□□■■■■■■■	777777607760771	LAM9	42	1	F/44		
PER01200310144	2003	0144	1	■■■■■■■■■■■■■■■■■■■■□□□□■■■■■■■■□□□□■■■■■■■	777777607760771	LAM9	42	1	M/37		
PER01200420169	2004	0169	1	■■■■■■■■■■■■■■■■■■■■□□□□■■■■■■■■□□□□■■■■■■■	777777607760771	LAM9	42	2	F/39		
PER01200420189	2004	0189	1	■■■■■■■■■■■■■■■■■■■■□□□□■■■■■■■■□□□□■■■■■■■	777777607760771	LAM9	42	2	M/25		
PER01199920235	1999	0235	1	■■■■■■■■■■■■■■■■■■■■□□□□■■■■■■■■□□□□■■■■■■■	777777607760771	LAM9	42	2	F/35		HIV+
PER01199920241	1999	0241	1	■■■■■■■■■■■■■■■■■■■■□□□□■■■■■■■■□□□□■■■■■■■	777777607760771	LAM9	42	2	M/32		HIV+
PER01199910243	1999	0243	1	■■■■■■■■■■■■■■■■■■■■□□□□■■■■■■■■□□□□■■■■■■■	777777607760771	LAM9	42	1	M/20		HIV+
PER01199920244	1999	0244	1	■■■■■■■■■■■■■■■■■■■■□□□□■■■■■■■■□□□□■■■■■■■	777777607760771	LAM9	42	2	M/29		HIV+
PER01199920245	1999	0245	1	■■■■■■■■■■■■■■■■■■■■□□□□■■■■■■■■□□□□■■■■■■■	777777607760771	LAM9	42	2	M/21		HIV+
PER01199920257	1999	0257	1	■■■■■■■■■■■■■■■■■■■■□□□□■■■■■■■■□□□□■■■■■■■	777777607760771	LAM9	42	2	M/33		HIV+
PER01199920259	1999	0259	1	■■■■■■■■■■■■■■■■■■■■□□□□■■■■■■■■□□□□■■■■■■■	777777607760771	LAM9	42	2	M/32		HIV+
PER01200020274	2000	0274	1	■■■■■■■■■■■■■■■■■■■■□□□□■■■■■■■■□□□□■■■■■■■	777777607760771	LAM9	42	2	M/29		HIV+
PER01200020281	2000	0281	1	■■■■■■■■■■■■■■■■■■■■□□□□■■■■■■■■□□□□■■■■■■■	777777607760771	LAM9	42	2	M/28		HIV+
PER01200020283	2000	0283	1	■■■■■■■■■■■■■■■■■■■■□□□□■■■■■■■■□□□□■■■■■■■	777777607760771	LAM9	42	2	M/29		HIV+
PER01200020287	2000	0287	1	■■■■■■■■■■■■■■■■■■■■□□□□■■■■■■■■□□□□■■■■■■■	777777607760771	LAM9	42	2	M/24		HIV+
PER01200020288	2000	0288	1	■■■■■■■■■■■■■■■■■■■■□□□□■■■■■■■■□□□□■■■■■■■	777777607760771	LAM9	42	2	M/33		HIV+
PER01200020293	2000	0293	1	■■■■■■■■■■■■■■■■■■■■□□□□■■■■■■■■□□□□■■■■■■■	777777607760771	LAM9	42	2	M/32		HIV+

PER01200020294	2000	0294	1	■■■■■■■■■■■■■■■■■■■■□□□□■■■■■■■■□□□□■■■■■■■	777777607760771	LAM9	42	2	M/37		HIV+
PER01200020298	2000	0298	1	■■■■■■■■■■■■■■■■■■■■□□□□■■■■■■■■□□□□■■■■■■■	777777607760771	LAM9	42	2	M/37		HIV+
PER01200020300	2000	0300	1	■■■■■■■■■■■■■■■■■■■■□□□□■■■■■■■■□□□□■■■■■■■	777777607760771	LAM9	42	2	M/33		HIV+
PER01200020301	2000	0301	1	■■■■■■■■■■■■■■■■■■■■□□□□■■■■■■■■□□□□■■■■■■■	777777607760771	LAM9	42	2	M/23		HIV+
PER01200120303	2001	0303	1	■■■■■■■■■■■■■■■■■■■■□□□□■■■■■■■■□□□□■■■■■■■	777777607760771	LAM9	42	2	F/19		HIV+
PER01200120306	2001	0306	1	■■■■■■■■■■■■■■■■■■■■□□□□■■■■■■■■□□□□■■■■■■■	777777607760771	LAM9	42	2	M/39		HIV+
PER01200120309	2001	0309	1	■■■■■■■■■■■■■■■■■■■■□□□□■■■■■■■■□□□□■■■■■■■	777777607760771	LAM9	42	2	F/26		HIV+
PER01200120310	2001	0310	1	■■■■■■■■■■■■■■■■■■■■□□□□■■■■■■■■□□□□■■■■■■■	777777607760771	LAM9	42	2	M/0		HIV+
PER01200120313	2001	0313	1	■■■■■■■■■■■■■■■■■■■■□□□□■■■■■■■■□□□□■■■■■■■	777777607760771	LAM9	42	2	M/31		HIV+
PER01200120317	2001	0317	1	■■■■■■■■■■■■■■■■■■■■□□□□■■■■■■■■□□□□■■■■■■■	777777607760771	LAM9	42	2	M/27		HIV+
PER01200120330	2001	0330	1	■■■■■■■■■■■■■■■■■■■■□□□□■■■■■■■■□□□□■■■■■■■	777777607760771	LAM9	42	2	M/36		HIV+
PER01200220336	2002	0336	1	■■■■■■■■■■■■■■■■■■■■□□□□■■■■■■■■□□□□■■■■■■■	777777607760771	LAM9	42	2	M/25		HIV+
PER01200220337	2002	0337	1	■■■■■■■■■■■■■■■■■■■■□□□□■■■■■■■■□□□□■■■■■■■	777777607760771	LAM9	42	2	M/48		HIV+
PER01200220338	2002	0338	1	■■■■■■■■■■■■■■■■■■■■□□□□■■■■■■■■□□□□■■■■■■■	777777607760771	LAM9	42	2	F/25		HIV+
PER01200220339	2002	0339	1	■■■■■■■■■■■■■■■■■■■■□□□□■■■■■■■■□□□□■■■■■■■	777777607760771	LAM9	42	2	M/29		HIV+
PER01199910384	1999	0384	1	■■■■■■■■■■■■■■■■■■■■□□□□■■■■■■■■□□□□■■■■■■■	777777607760771	LAM9	42	1	M/0		
PER01199910409	1999	0409	1	■■■■■■■■■■■■■■■■■■■■□□□□■■■■■■■■□□□□■■■■■■■	777777607760771	LAM9	42	1	M/0		
PER01199920418	1999	0418	1	■■■■■■■■■■■■■■■■■■■■□□□□■■■■■■■■□□□□■■■■■■■	777777607760771	LAM9	42	2	M/0		
PER01199910436	1999	0436	1	■■■■■■■■■■■■■■■■■■■■□□□□■■■■■■■■□□□□■■■■■■■	777777607760771	LAM9	42	1	F/18		
PER01199930443	1999	0443	1	■■■■■■■■■■■■■■■■■■■■□□□□■■■■■■■■□□□□■■■■■■■	777777607760771	LAM9	42	3	M/0	RIF resist	
PER01199910469	1999	0469	1	■■■■■■■■■■■■■■■■■■■■□□□□■■■■■■■■□□□□■■■■■■■	777777607760771	LAM9	42	1	M/19		
PER01200010524	2000	0524	1	■■■■■■■■■■■■■■■■■■■■□□□□■■■■■■■■□□□□■■■■■■■	777777607760771	LAM9	42	1	M/0		
PER01200010558	2000	0558	1	■■■■■■■■■■■■■■■■■■■■□□□□■■■■■■■■□□□□■■■■■■■	777777607760771	LAM9	42	1	F/0		
PER01200030587	2000	0587	1	■■■■■■■■■■■■■■■■■■■■□□□□■■■■■■■■□□□□■■■■■■■	777777607760771	LAM9	42	3	F/15	INH resist	
PER01200120609	2001	0609	1	■■■■■■■■■■■■■■■■■■■■□□□□■■■■■■■■□□□□■■■■■■■	777777607760771	LAM9	42	2	M/0		
PER01200220672	2002	0672	1	■■■■■■■■■■■■■■■■■■■■□□□□■■■■■■■■□□□□■■■■■■■	777777607760771	LAM9	42	2	F/25		HIV+
PER01200220683	2002	0683	1	■■■■■■■■■■■■■■■■■■■■□□□□■■■■■■■■□□□□■■■■■■■	777777607760771	LAM9	42	2	F/26		HIV+
PER01200210684	2002	0684	1	■■■■■■■■■■■■■■■■■■■■□□□□■■■■■■■■□□□□■■■■■■■	777777607760771	LAM9	42	1	F/34		HIV+
PER01200220685	2002	0685	1	■■■■■■■■■■■■■■■■■■■■□□□□■■■■■■■■□□□□■■■■■■■	777777607760771	LAM9	42	2	M/43		HIV+
PER01200220686	2002	0686	1	■■■■■■■■■■■■■■■■■■■■□□□□■■■■■■■■□□□□■■■■■■■	777777607760771	LAM9	42	2	F/28		HIV+
PER01200220696	2002	0696	1	■■■■■■■■■■■■■■■■■■■■□□□□■■■■■■■■□□□□■■■■■■■	777777607760771	LAM9	42	2	F/32		HIV+
PER01200220700	2002	0700	1	■■■■■■■■■■■■■■■■■■■■□□□□■■■■■■■■□□□□■■■■■■■	777777607760771	LAM9	42	2	M/22		HIV+
PER01200210711	2002	0711	1	■■■■■■■■■■■■■■■■■■■■□□□□■■■■■■■■□□□□■■■■■■■	777777607760771	LAM9	42	1	M/37		HIV+
PER01200330743	2003	0743	1	■■■■■■■■■■■■■■■■■■■■□□□□■■■■■■■■□□□□■■■■■■■	777777607760771	LAM9	42	3	M/20	INH resist	HIV+
PER01200330750	2003	0750	1	■■■■■■■■■■■■■■■■■■■■□□□□■■■■■■■■□□□□■■■■■■■	777777607760771	LAM9	42	3	M/31	INH resist	HIV+
PER01200420772	2004	0772	1	■■■■■■■■■■■■■■■■■■■■□□□□■■■■■■■■□□□□■■■■■■■	777777607760771	LAM9	42	2	M/24		HIV+
PER01200420776	2004	0776	1	■■■■■■■■■■■■■■■■■■■■□□□□■■■■■■■■□□□□■■■■■■■	777777607760771	LAM9	42	2	M/53		HIV+
PER01199910453	1999	0453	1	■■■■■■■■■■■■■■■■■■■■■■■■□□□□□□□□□□□□□□□□□□□	777777770000000	Unknown	46	1	M/0		
PER01200310001	2003	0001	1	■■■■■■■■■■■■■■■■■■■■■■■■■□□□□□□■□□□□■■■■■■■	777777774020771	H1	47	1	F/18		
PER01200320011	2003	0011	1	■■■■■■■■■■■■■■■■■■■■■■■■■□□□□□□■□□□□■■■■■■■	777777774020771	H1	47	2	F/17		
PER01200410018	2004	0018	1	■■■■■■■■■■■■■■■■■■■■■■■■■□□□□□□■□□□□■■■■■■■	777777774020771	H1	47	1	F/33		
PER01200430025	2004	0025	1	■■■■■■■■■■■■■■■■■■■■■■■■■□□□□□□■□□□□■■■■■■■	777777774020771	H1	47	3	M/64	INH resist	
PER01200310085	2003	0085	1	■■■■■■■■■■■■■■■■■■■■■■■■■□□□□□□■□□□□■■■■■■■	777777774020771	H1	47	1	F/28		
PER01200310108	2003	0108	1	■■■■■■■■■■■■■■■■■■■■■■■■■□□□□□□■□□□□■■■■■■■	777777774020771	H1	47	1	F/44		
PER01200310109	2003	0109	1	■■■■■■■■■■■■■■■■■■■■■■■■■□□□□□□■□□□□■■■■■■■	777777774020771	H1	47	1	F/30		
PER01200310119	2003	0119	1	■■■■■■■■■■■■■■■■■■■■■■■■■□□□□□□■□□□□■■■■■■■	777777774020771	H1	47	1	F/26		
PER01200310131	2003	0131	1	■■■■■■■■■■■■■■■■■■■■■■■■■□□□□□□■□□□□■■■■■■■	777777774020771	H1	47	1	F/70		
PER01200310151	2003	0151	1	■■■■■■■■■■■■■■■■■■■■■■■■■□□□□□□■□□□□■■■■■■■	777777774020771	H1	47	1	F/19		
PER01200410156	2004	0156	1	■■■■■■■■■■■■■■■■■■■■■■■■■□□□□□□■□□□□■■■■■■■	777777774020771	H1	47	1	F/39		
PER01200410174	2004	0174	1	■■■■■■■■■■■■■■■■■■■■■■■■■□□□□□□■□□□□■■■■■■■	777777774020771	H1	47	1	F/21		
PER01200410181	2004	0181	1	■■■■■■■■■■■■■■■■■■■■■■■■■□□□□□□■□□□□■■■■■■■	777777774020771	H1	47	1	M/20		

PER01200410203	2004	0203	1	■■■■■■■■■■■■■■■■■■■■■■■■■□□□□□□■□□□□■■■■■■■	777777774020771	H1	47	1	F/36		
PER01199910233	1999	0233	1	■■■■■■■■■■■■■■■■■■■■■■■■■□□□□□□■□□□□■■■■■■■	777777774020771	H1	47	1	M/22		HIV+
PER01200010276	2000	0276	1	■■■■■■■■■■■■■■■■■■■■■■■■■□□□□□□■□□□□■■■■■■■	777777774020771	H1	47	1	M/39		HIV+
PER01200130322	2001	0322	1	■■■■■■■■■■■■■■■■■■■■■■■■■□□□□□□■□□□□■■■■■■■	777777774020771	H1	47	3	F/39	INH resist	HIV+
PER01200110327	2001	0327	1	■■■■■■■■■■■■■■■■■■■■■■■■■□□□□□□■□□□□■■■■■■■	777777774020771	H1	47	1	M/30		HIV+
PER01199910388	1999	0388	1	■■■■■■■■■■■■■■■■■■■■■■■■■□□□□□□■□□□□■■■■■■■	777777774020771	H1	47	1	M/0		
PER01199910435	1999	0435	1	■■■■■■■■■■■■■■■■■■■■■■■■■□□□□□□■□□□□■■■■■■■	777777774020771	H1	47	1	M/0		
PER01200020487	2000	0487	1	■■■■■■■■■■■■■■■■■■■■■■■■■□□□□□□■□□□□■■■■■■■	777777774020771	H1	47	2	F/0		
PER01200010534	2000	0534	1	■■■■■■■■■■■■■■■■■■■■■■■■■□□□□□□■□□□□■■■■■■■	777777774020771	H1	47	1	M/0		
PER01200010535	2000	0535	1	■■■■■■■■■■■■■■■■■■■■■■■■■□□□□□□■□□□□■■■■■■■	777777774020771	H1	47	1	M/0		
PER01200010548	2000	0548	1	■■■■■■■■■■■■■■■■■■■■■■■■■□□□□□□■□□□□■■■■■■■	777777774020771	H1	47	1	M/0		
PER01200010552	2000	0552	1	■■■■■■■■■■■■■■■■■■■■■■■■■□□□□□□■□□□□■■■■■■■	777777774020771	H1	47	1	F/0		
PER01200010573	2000	0573	1	■■■■■■■■■■■■■■■■■■■■■■■■■□□□□□□■□□□□■■■■■■■	777777774020771	H1	47	1	F/0		
PER01200010576	2000	0576	1	■■■■■■■■■■■■■■■■■■■■■■■■■□□□□□□■□□□□■■■■■■■	777777774020771	H1	47	1	M/0		
PER01200010580	2000	0580	1	■■■■■■■■■■■■■■■■■■■■■■■■■□□□□□□■□□□□■■■■■■■	777777774020771	H1	47	1	F/0		
PER01200110631	2001	0631	1	■■■■■■■■■■■■■■■■■■■■■■■■■□□□□□□■□□□□■■■■■■■	777777774020771	H1	47	1	F/0		
PER01200210664	2002	0664	1	■■■■■■■■■■■■■■■■■■■■■■■■■□□□□□□■□□□□■■■■■■■	777777774020771	H1	47	1	M/21		
PER01200310737	2003	0737	1	■■■■■■■■■■■■■■■■■■■■■■■■■□□□□□□■□□□□■■■■■■■	777777774020771	H1	47	1	F/34		HIV+
PER01200420034	2004	0034	1	■■■■■■■■■■■■■■■■■■■■■■■■■■■■■■□■□□□□■■■□■■■	777777777720731	H3	49	2	M/22		
PER01200510063	2005	0063	1	■■■■■■■■■■■■■■■■■■■■■■■■■■■■■■□■□□□□■■■□■■■	777777777720731	H3	49	1	F/45		
PER01200530065	2005	0065	1	■■■■■■■■■■■■■■■■■■■■■■■■■■■■■■□■□□□□■■■□■■■	777777777720731	H3	49	3	M/26	INH resist	
PER01200410201	2004	0201	1	■■■■■■■■■■■■■■■■■■■■■■■■■■■■■■□■□□□□■■■□■■■	777777777720731	H3	49	1	M/26		
PER01200010280	2000	0280	1	■■■■■■■■■■■■■■■■■■■■■■■■■■■■■■□■□□□□■■■□■■■	777777777720731	H3	49	1	M/23		HIV+
PER01200030493	2000	0493	1	■■■■■■■■■■■■■■■■■■■■■■■■■■■■■■□■□□□□■■■□■■■	777777777720731	H3	49	3	F/0	INH resist	
PER01200310724	2003	0724	1	■■■■■■■■■■■■■■■■■■■■■■■■■■■■■■□■□□□□■■■□■■■	777777777720731	H3	49	1	M/47		HIV+
PER01200320005	2003	0005	1	■■■■■■■■■■■■■■■■■■■■■■■■■■■■■■□■□□□□■■■■■■■	777777777720771	H3	50	2	F/48		
PER01200320013	2003	0013	1	■■■■■■■■■■■■■■■■■■■■■■■■■■■■■■□■□□□□■■■■■■■	777777777720771	H3	50	2	F/16		
PER01200410017	2004	0017	1	■■■■■■■■■■■■■■■■■■■■■■■■■■■■■■□■□□□□■■■■■■■	777777777720771	H3	50	1	M/27		
PER01200410020	2004	0020	1	■■■■■■■■■■■■■■■■■■■■■■■■■■■■■■□■□□□□■■■■■■■	777777777720771	H3	50	1	F/27		
PER01200410021	2004	0021	1	■■■■■■■■■■■■■■■■■■■■■■■■■■■■■■□■□□□□■■■■■■■	777777777720771	H3	50	1	M/55		
PER01200410023	2004	0023	1	■■■■■■■■■■■■■■■■■■■■■■■■■■■■■■□■□□□□■■■■■■■	777777777720771	H3	50	1	M/69		
PER01200410031	2004	0031	1	■■■■■■■■■■■■■■■■■■■■■■■■■■■■■■□■□□□□■■■■■■■	777777777720771	H3	50	1	F/62		
PER01200510054	2005	0054	1	■■■■■■■■■■■■■■■■■■■■■■■■■■■■■■□■□□□□■■■■■■■	777777777720771	H3	50	1	F/53		
PER01200510055	2005	0055	1	■■■■■■■■■■■■■■■■■■■■■■■■■■■■■■□■□□□□■■■■■■■	777777777720771	H3	50	1	M/46		
PER01200510061	2005	0061	1	■■■■■■■■■■■■■■■■■■■■■■■■■■■■■■□■□□□□■■■■■■■	777777777720771	H3	50	1	M/50		
PER01200510062	2005	0062	1	■■■■■■■■■■■■■■■■■■■■■■■■■■■■■■□■□□□□■■■■■■■	777777777720771	H3	50	1	F/22		
PER01200510064	2005	0064	1	■■■■■■■■■■■■■■■■■■■■■■■■■■■■■■□■□□□□■■■■■■■	777777777720771	H3	50	1	F/68		
PER01200320079	2003	0079	1	■■■■■■■■■■■■■■■■■■■■■■■■■■■■■■□■□□□□■■■■■■■	777777777720771	H3	50	2	F/26		
PER01200320080	2003	0080	1	■■■■■■■■■■■■■■■■■■■■■■■■■■■■■■□■□□□□■■■■■■■	777777777720771	H3	50	2	F/26		
PER01200310082	2003	0082	1	■■■■■■■■■■■■■■■■■■■■■■■■■■■■■■□■□□□□■■■■■■■	777777777720771	H3	50	1	F/31		
PER01200310087	2003	0087	1	■■■■■■■■■■■■■■■■■■■■■■■■■■■■■■□■□□□□■■■■■■■	777777777720771	H3	50	1	F/25		
PER01200310091	2003	0091	1	■■■■■■■■■■■■■■■■■■■■■■■■■■■■■■□■□□□□■■■■■■■	777777777720771	H3	50	1	M/62		
PER01200310099	2003	0099	1	■■■■■■■■■■■■■■■■■■■■■■■■■■■■■■□■□□□□■■■■■■■	777777777720771	H3	50	1	F/30		
PER01200310100	2003	0100	1	■■■■■■■■■■■■■■■■■■■■■■■■■■■■■■□■□□□□■■■■■■■	777777777720771	H3	50	1	M/18		
PER01200310105	2003	0105	1	■■■■■■■■■■■■■■■■■■■■■■■■■■■■■■□■□□□□■■■■■■■	777777777720771	H3	50	1	F/27		
PER01200310113	2003	0113	1	■■■■■■■■■■■■■■■■■■■■■■■■■■■■■■□■□□□□■■■■■■■	777777777720771	H3	50	1	F/20		
PER01200310115	2003	0115	1	■■■■■■■■■■■■■■■■■■■■■■■■■■■■■■□■□□□□■■■■■■■	777777777720771	H3	50	1	F/62		
PER01200310140	2003	0140	1	■■■■■■■■■■■■■■■■■■■■■■■■■■■■■■□■□□□□■■■■■■■	777777777720771	H3	50	1	M/38		
PER01200310145	2003	0145	1	■■■■■■■■■■■■■■■■■■■■■■■■■■■■■■□■□□□□■■■■■■■	777777777720771	H3	50	1	F/20		
PER01200410158	2004	0158	1	■■■■■■■■■■■■■■■■■■■■■■■■■■■■■■□■□□□□■■■■■■■	777777777720771	H3	50	1	M/18		
PER01200410159	2004	0159	1	■■■■■■■■■■■■■■■■■■■■■■■■■■■■■■□■□□□□■■■■■■■	777777777720771	H3	50	1	M/34		

PER01200410163	2004	0163	1	■■■■■■■■■■■■■■■■■■■■■■■■■■■■■■□■□□□□■■■■■■■	777777777720771	H3	50	1	F/39		
PER01200410172	2004	0172	1	■■■■■■■■■■■■■■■■■■■■■■■■■■■■■■□■□□□□■■■■■■■	777777777720771	H3	50	1	F/22		
PER01200410190	2004	0190	1	■■■■■■■■■■■■■■■■■■■■■■■■■■■■■■□■□□□□■■■■■■■	777777777720771	H3	50	1	F/28		
PER01200410192	2004	0192	1	■■■■■■■■■■■■■■■■■■■■■■■■■■■■■■□■□□□□■■■■■■■	777777777720771	H3	50	1	F/30		
PER01200410198	2004	0198	1	■■■■■■■■■■■■■■■■■■■■■■■■■■■■■■□■□□□□■■■■■■■	777777777720771	H3	50	1	M/19		
PER01200410200	2004	0200	1	■■■■■■■■■■■■■■■■■■■■■■■■■■■■■■□■□□□□■■■■■■■	777777777720771	H3	50	1	F/41		
PER01200410207	2004	0207	1	■■■■■■■■■■■■■■■■■■■■■■■■■■■■■■□■□□□□■■■■■■■	777777777720771	H3	50	1	M/52		
PER01200410209	2004	0209	1	■■■■■■■■■■■■■■■■■■■■■■■■■■■■■■□■□□□□■■■■■■■	777777777720771	H3	50	1	M/37		
PER01200410211	2004	0211	1	■■■■■■■■■■■■■■■■■■■■■■■■■■■■■■□■□□□□■■■■■■■	777777777720771	H3	50	1	F/30		
PER01200430214	2004	0214	1	■■■■■■■■■■■■■■■■■■■■■■■■■■■■■■□■□□□□■■■■■■■	777777777720771	H3	50	3	F/51	RIF resist	
PER01200430217	2004	0217	1	■■■■■■■■■■■■■■■■■■■■■■■■■■■■■■□■□□□□■■■■■■■	777777777720771	H3	50	3	F/24	RIF resist	
PER01200420220	2004	0220	1	■■■■■■■■■■■■■■■■■■■■■■■■■■■■■■□■□□□□■■■■■■■	777777777720771	H3	50	2	F/19		
PER01200430224	2004	0224	1	■■■■■■■■■■■■■■■■■■■■■■■■■■■■■■□■□□□□■■■■■■■	777777777720771	H3	50	3	F/63	RIF resist	
PER01199910255	1999	0255	1	■■■■■■■■■■■■■■■■■■■■■■■■■■■■■■□■□□□□■■■■■■■	777777777720771	H3	50	1	M/32		HIV+
PER01199910263	1999	0263	1	■■■■■■■■■■■■■■■■■■■■■■■■■■■■■■□■□□□□■■■■■■■	777777777720771	H3	50	1	M/29		HIV+
PER01199920264	1999	0264	1	■■■■■■■■■■■■■■■■■■■■■■■■■■■■■■□■□□□□■■■■■■■	777777777720771	H3	50	2	M/25		HIV+
PER01200030295	2000	0295	1	■■■■■■■■■■■■■■■■■■■■■■■■■■■■■■□■□□□□■■■■■■■	777777777720771	H3	50	3	F/27	RIF resist	HIV+
PER01200020296	2000	0296	1	■■■■■■■■■■■■■■■■■■■■■■■■■■■■■■□■□□□□■■■■■■■	777777777720771	H3	50	2	M/29		HIV+
PER01200010299	2000	0299	1	■■■■■■■■■■■■■■■■■■■■■■■■■■■■■■□■□□□□■■■■■■■	777777777720771	H3	50	1	M/26		HIV+
PER01200110320	2001	0320	1	■■■■■■■■■■■■■■■■■■■■■■■■■■■■■■□■□□□□■■■■■■■	777777777720771	H3	50	1	M/26		HIV+
PER01200210334	2002	0334	1	■■■■■■■■■■■■■■■■■■■■■■■■■■■■■■□■□□□□■■■■■■■	777777777720771	H3	50	1	M/43		HIV+
PER01199910340	1999	0340	1	■■■■■■■■■■■■■■■■■■■■■■■■■■■■■■□■□□□□■■■■■■■	777777777720771	H3	50	1	F/21		
PER01199910348	1999	0348	1	■■■■■■■■■■■■■■■■■■■■■■■■■■■■■■□■□□□□■■■■■■■	777777777720771	H3	50	1	M/0		
PER01199910349	1999	0349	1	■■■■■■■■■■■■■■■■■■■■■■■■■■■■■■□■□□□□■■■■■■■	777777777720771	H3	50	1	F/44		
PER01199910362	1999	0362	1	■■■■■■■■■■■■■■■■■■■■■■■■■■■■■■□■□□□□■■■■■■■	777777777720771	H3	50	1	M/22		
PER01199910366	1999	0366	1	■■■■■■■■■■■■■■■■■■■■■■■■■■■■■■□■□□□□■■■■■■■	777777777720771	H3	50	1	M/0		
PER01199910375	1999	0375	1	■■■■■■■■■■■■■■■■■■■■■■■■■■■■■■□■□□□□■■■■■■■	777777777720771	H3	50	1	M/0		
PER01199910378	1999	0378	1	■■■■■■■■■■■■■■■■■■■■■■■■■■■■■■□■□□□□■■■■■■■	777777777720771	H3	50	1	M/0		
PER01199910399	1999	0399	1	■■■■■■■■■■■■■■■■■■■■■■■■■■■■■■□■□□□□■■■■■■■	777777777720771	H3	50	1	F/0		
PER01199910400	1999	0400	1	■■■■■■■■■■■■■■■■■■■■■■■■■■■■■■□■□□□□■■■■■■■	777777777720771	H3	50	1	M/0		
PER01199910402	1999	0402	1	■■■■■■■■■■■■■■■■■■■■■■■■■■■■■■□■□□□□■■■■■■■	777777777720771	H3	50	1	F/0		
PER01199930419	1999	0419	1	■■■■■■■■■■■■■■■■■■■■■■■■■■■■■■□■□□□□■■■■■■■	777777777720771	H3	50	3	M/0	INH resist	
PER01199910430	1999	0430	1	■■■■■■■■■■■■■■■■■■■■■■■■■■■■■■□■□□□□■■■■■■■	777777777720771	H3	50	1	F/0		
PER01199910433	1999	0433	1	■■■■■■■■■■■■■■■■■■■■■■■■■■■■■■□■□□□□■■■■■■■	777777777720771	H3	50	1	M/0		
PER01199910441	1999	0441	1	■■■■■■■■■■■■■■■■■■■■■■■■■■■■■■□■□□□□■■■■■■■	777777777720771	H3	50	1	F/22		
PER01199910449	1999	0449	1	■■■■■■■■■■■■■■■■■■■■■■■■■■■■■■□■□□□□■■■■■■■	777777777720771	H3	50	1	F/0		
PER01199910452	1999	0452	1	■■■■■■■■■■■■■■■■■■■■■■■■■■■■■■□■□□□□■■■■■■■	777777777720771	H3	50	1	F/28		
PER01199910455	1999	0455	1	■■■■■■■■■■■■■■■■■■■■■■■■■■■■■■□■□□□□■■■■■■■	777777777720771	H3	50	1	F/35		
PER01199930468	1999	0468	1	■■■■■■■■■■■■■■■■■■■■■■■■■■■■■■□■□□□□■■■■■■■	777777777720771	H3	50	3	F/23	INH resist	
PER01200010476	2000	0476	1	■■■■■■■■■■■■■■■■■■■■■■■■■■■■■■□■□□□□■■■■■■■	777777777720771	H3	50	1	F/0		
PER01200010480	2000	0480	1	■■■■■■■■■■■■■■■■■■■■■■■■■■■■■■□■□□□□■■■■■■■	777777777720771	H3	50	1	M/23		
PER01200010483	2000	0483	1	■■■■■■■■■■■■■■■■■■■■■■■■■■■■■■□■□□□□■■■■■■■	777777777720771	H3	50	1	M/0		
PER01200010508	2000	0508	1	■■■■■■■■■■■■■■■■■■■■■■■■■■■■■■□■□□□□■■■■■■■	777777777720771	H3	50	1	M/0		
PER01200010509	2000	0509	1	■■■■■■■■■■■■■■■■■■■■■■■■■■■■■■□■□□□□■■■■■■■	777777777720771	H3	50	1	F/0		
PER01200010511	2000	0511	1	■■■■■■■■■■■■■■■■■■■■■■■■■■■■■■□■□□□□■■■■■■■	777777777720771	H3	50	1	F/0		
PER01200010518	2000	0518	1	■■■■■■■■■■■■■■■■■■■■■■■■■■■■■■□■□□□□■■■■■■■	777777777720771	H3	50	1	M/0		
PER01200010525	2000	0525	1	■■■■■■■■■■■■■■■■■■■■■■■■■■■■■■□■□□□□■■■■■■■	777777777720771	H3	50	1	M/0		
PER01200010528	2000	0528	1	■■■■■■■■■■■■■■■■■■■■■■■■■■■■■■□■□□□□■■■■■■■	777777777720771	H3	50	1	M/0		
PER01200010529	2000	0529	1	■■■■■■■■■■■■■■■■■■■■■■■■■■■■■■□■□□□□■■■■■■■	777777777720771	H3	50	1	M/0		
PER01200030531	2000	0531	1	■■■■■■■■■■■■■■■■■■■■■■■■■■■■■■□■□□□□■■■■■■■	777777777720771	H3	50	3	F/0	INH resist	
PER01200010532	2000	0532	1	■■■■■■■■■■■■■■■■■■■■■■■■■■■■■■□■□□□□■■■■■■■	777777777720771	H3	50	1	M/19		

PER01200010533	2000	0533	1	■■■■■■■■■■■■■■■■■■■■■■■■■■■■■■□■□□□□■■■■■■■	777777777720771	H3	50	1	M/16		
PER01200010536	2000	0536	1	■■■■■■■■■■■■■■■■■■■■■■■■■■■■■■□■□□□□■■■■■■■	777777777720771	H3	50	1	M/0		
PER01200010547	2000	0547	1	■■■■■■■■■■■■■■■■■■■■■■■■■■■■■■□■□□□□■■■■■■■	777777777720771	H3	50	1	F/0		
PER01200010550	2000	0550	1	■■■■■■■■■■■■■■■■■■■■■■■■■■■■■■□■□□□□■■■■■■■	777777777720771	H3	50	1	M/0		
PER01200010551	2000	0551	1	■■■■■■■■■■■■■■■■■■■■■■■■■■■■■■□■□□□□■■■■■■■	777777777720771	H3	50	1	F/30		
PER01200010553	2000	0553	1	■■■■■■■■■■■■■■■■■■■■■■■■■■■■■■□■□□□□■■■■■■■	777777777720771	H3	50	1	F/0		
PER01200010554	2000	0554	1	■■■■■■■■■■■■■■■■■■■■■■■■■■■■■■□■□□□□■■■■■■■	777777777720771	H3	50	1	M/0		
PER01200010561	2000	0561	1	■■■■■■■■■■■■■■■■■■■■■■■■■■■■■■□■□□□□■■■■■■■	777777777720771	H3	50	1	M/58		
PER01200010562	2000	0562	1	■■■■■■■■■■■■■■■■■■■■■■■■■■■■■■□■□□□□■■■■■■■	777777777720771	H3	50	1	F/72		
PER01200010567	2000	0567	1	■■■■■■■■■■■■■■■■■■■■■■■■■■■■■■□■□□□□■■■■■■■	777777777720771	H3	50	1	M/0		
PER01200010568	2000	0568	1	■■■■■■■■■■■■■■■■■■■■■■■■■■■■■■□■□□□□■■■■■■■	777777777720771	H3	50	1	M/0		
PER01200010574	2000	0574	1	■■■■■■■■■■■■■■■■■■■■■■■■■■■■■■□■□□□□■■■■■■■	777777777720771	H3	50	1	M/0		
PER01200010582	2000	0582	1	■■■■■■■■■■■■■■■■■■■■■■■■■■■■■■□■□□□□■■■■■■■	777777777720771	H3	50	1	M/0		
PER01200110596	2001	0596	1	■■■■■■■■■■■■■■■■■■■■■■■■■■■■■■□■□□□□■■■■■■■	777777777720771	H3	50	1	F/0		
PER01200130598	2001	0598	1	■■■■■■■■■■■■■■■■■■■■■■■■■■■■■■□■□□□□■■■■■■■	777777777720771	H3	50	3	M/0	INH resist	
PER01200110606	2001	0606	1	■■■■■■■■■■■■■■■■■■■■■■■■■■■■■■□■□□□□■■■■■■■	777777777720771	H3	50	1	M/0		
PER01200110607	2001	0607	1	■■■■■■■■■■■■■■■■■■■■■■■■■■■■■■□■□□□□■■■■■■■	777777777720771	H3	50	1	F/0		
PER01200110610	2001	0610	1	■■■■■■■■■■■■■■■■■■■■■■■■■■■■■■□■□□□□■■■■■■■	777777777720771	H3	50	1	M/0		
PER01200110613	2001	0613	1	■■■■■■■■■■■■■■■■■■■■■■■■■■■■■■□■□□□□■■■■■■■	777777777720771	H3	50	1	M/0		
PER01200120614	2001	0614	1	■■■■■■■■■■■■■■■■■■■■■■■■■■■■■■□■□□□□■■■■■■■	777777777720771	H3	50	2	F/18		
PER01200110615	2001	0615	1	■■■■■■■■■■■■■■■■■■■■■■■■■■■■■■□■□□□□■■■■■■■	777777777720771	H3	50	1	F/0		
PER01200110617	2001	0617	1	■■■■■■■■■■■■■■■■■■■■■■■■■■■■■■□■□□□□■■■■■■■	777777777720771	H3	50	1	M/0		
PER01200130626	2001	0626	1	■■■■■■■■■■■■■■■■■■■■■■■■■■■■■■□■□□□□■■■■■■■	777777777720771	H3	50	3	M/0	INH resist	
PER01200110633	2001	0633	1	■■■■■■■■■■■■■■■■■■■■■■■■■■■■■■□■□□□□■■■■■■■	777777777720771	H3	50	1	F/21		
PER01200120634	2001	0634	1	■■■■■■■■■■■■■■■■■■■■■■■■■■■■■■□■□□□□■■■■■■■	777777777720771	H3	50	2	F/22		
PER01200110636	2001	0636	1	■■■■■■■■■■■■■■■■■■■■■■■■■■■■■■□■□□□□■■■■■■■	777777777720771	H3	50	1	M/35		
PER01200110639	2001	0639	1	■■■■■■■■■■■■■■■■■■■■■■■■■■■■■■□■□□□□■■■■■■■	777777777720771	H3	50	1	M/61		
PER01200110649	2001	0649	1	■■■■■■■■■■■■■■■■■■■■■■■■■■■■■■□■□□□□■■■■■■■	777777777720771	H3	50	1	M/58		
PER01200110650	2001	0650	1	■■■■■■■■■■■■■■■■■■■■■■■■■■■■■■□■□□□□■■■■■■■	777777777720771	H3	50	1	M/43		
PER01200210651	2002	0651	1	■■■■■■■■■■■■■■■■■■■■■■■■■■■■■■□■□□□□■■■■■■■	777777777720771	H3	50	1	M/22		
PER01200210654	2002	0654	1	■■■■■■■■■■■■■■■■■■■■■■■■■■■■■■□■□□□□■■■■■■■	777777777720771	H3	50	1	F/19		
PER01200210656	2002	0656	1	■■■■■■■■■■■■■■■■■■■■■■■■■■■■■■□■□□□□■■■■■■■	777777777720771	H3	50	1	F/26		
PER01200210659	2002	0659	1	■■■■■■■■■■■■■■■■■■■■■■■■■■■■■■□■□□□□■■■■■■■	777777777720771	H3	50	1	M/23		
PER01200210662	2002	0662	1	■■■■■■■■■■■■■■■■■■■■■■■■■■■■■■□■□□□□■■■■■■■	777777777720771	H3	50	1	M/23		
PER01200210669	2002	0669	1	■■■■■■■■■■■■■■■■■■■■■■■■■■■■■■□■□□□□■■■■■■■	777777777720771	H3	50	1	M/20		
PER01200210670	2002	0670	1	■■■■■■■■■■■■■■■■■■■■■■■■■■■■■■□■□□□□■■■■■■■	777777777720771	H3	50	1	F/16		
PER01200210681	2002	0681	1	■■■■■■■■■■■■■■■■■■■■■■■■■■■■■■□■□□□□■■■■■■■	777777777720771	H3	50	1	M/31		HIV+
PER01200210688	2002	0688	1	■■■■■■■■■■■■■■■■■■■■■■■■■■■■■■□■□□□□■■■■■■■	777777777720771	H3	50	1	M/29		HIV+
PER01200210689	2002	0689	1	■■■■■■■■■■■■■■■■■■■■■■■■■■■■■■□■□□□□■■■■■■■	777777777720771	H3	50	1	M/50		HIV+
PER01200210704	2002	0704	1	■■■■■■■■■■■■■■■■■■■■■■■■■■■■■■□■□□□□■■■■■■■	777777777720771	H3	50	1	M/42		HIV+
PER01200310729	2003	0729	1	■■■■■■■■■■■■■■■■■■■■■■■■■■■■■■□■□□□□■■■■■■■	777777777720771	H3	50	1	F/29		HIV+
PER01200310732	2003	0732	1	■■■■■■■■■■■■■■■■■■■■■■■■■■■■■■□■□□□□■■■■■■■	777777777720771	H3	50	1	M/27		HIV+
PER01200310738	2003	0738	1	■■■■■■■■■■■■■■■■■■■■■■■■■■■■■■□■□□□□■■■■■■■	777777777720771	H3	50	1	M/26		HIV+
PER01200310744	2003	0744	1	■■■■■■■■■■■■■■■■■■■■■■■■■■■■■■□■□□□□■■■■■■■	777777777720771	H3	50	1	F/35		HIV+
PER01200310749	2003	0749	1	■■■■■■■■■■■■■■■■■■■■■■■■■■■■■■□■□□□□■■■■■■■	777777777720771	H3	50	1	M/38		HIV+
PER01200310754	2003	0754	1	■■■■■■■■■■■■■■■■■■■■■■■■■■■■■■□■□□□□■■■■■■■	777777777720771	H3	50	1	M/27		HIV+
PER01200410773	2004	0773	1	■■■■■■■■■■■■■■■■■■■■■■■■■■■■■■□■□□□□■■■■■■■	777777777720771	H3	50	1	M/37		HIV+
PER01200410780	2004	0780	1	■■■■■■■■■■■■■■■■■■■■■■■■■■■■■■□■□□□□■■■■■■■	777777777720771	H3	50	1	M/30		HIV+
PER01200410784	2004	0784	1	■■■■■■■■■■■■■■■■■■■■■■■■■■■■■■□■□□□□■■■■■■■	777777777720771	H3	50	1	M/40		HIV+
PER01200410786	2004	0786	1	■■■■■■■■■■■■■■■■■■■■■■■■■■■■■■□■□□□□■■■■■■■	777777777720771	H3	50	1	M/25		HIV+
PER01200410787	2004	0787	1	■■■■■■■■■■■■■■■■■■■■■■■■■■■■■■□■□□□□■■■■■■■	777777777720771	H3	50	1	M/36		HIV+

PER01200420789	2004	0789	1	■■■■■■■■■■■■■■■■■■■■■■■■■■■■■■□■□□□□■■■■■■■	777777777720771	H3	50	2	M/43		HIV+
PER01200510798	2005	0798	1	■■■■■■■■■■■■■■■■■■■■■■■■■■■■■■□■□□□□■■■■■■■	777777777720771	H3	50	1	M/28		HIV+
PER01200110611	2001	0611	1	■■■■■■■■■■■■■■■■■■■■■■■■■■■■■■■■□□□□■■■□□□□	777777777760700	T1	51	1	M/0		
PER01200320006	2003	0006	1	■■■■■■■■■■■■■■■■■■■■■■■■■■■■■■■■□□□□■■■■■■■	777777777760771	T1	53	2	M/21		
PER01200330010	2003	0010	1	■■■■■■■■■■■■■■■■■■■■■■■■■■■■■■■■□□□□■■■■■■■	777777777760771	T1	53	3	F/19	INH resist	
PER01200310016	2003	0016	1	■■■■■■■■■■■■■■■■■■■■■■■■■■■■■■■■□□□□■■■■■■■	777777777760771	T1	53	1	M/59		
PER01200430022	2004	0022	1	■■■■■■■■■■■■■■■■■■■■■■■■■■■■■■■■□□□□■■■■■■■	777777777760771	T1	53	3	F/31	INH resist	
PER01200530052	2005	0052	1	■■■■■■■■■■■■■■■■■■■■■■■■■■■■■■■■□□□□■■■■■■■	777777777760771	T1	53	3	M/20	INH resist	
PER01200320081	2003	0081	1	■■■■■■■■■■■■■■■■■■■■■■■■■■■■■■■■□□□□■■■■■■■	777777777760771	T1	53	2	M/29		
PER01200330088	2003	0088	1	■■■■■■■■■■■■■■■■■■■■■■■■■■■■■■■■□□□□■■■■■■■	777777777760771	T1	53	3	M/27	INH resist	
PER01200310096	2003	0096	1	■■■■■■■■■■■■■■■■■■■■■■■■■■■■■■■■□□□□■■■■■■■	777777777760771	T1	53	1	M/31		
PER01200310107	2003	0107	1	■■■■■■■■■■■■■■■■■■■■■■■■■■■■■■■■□□□□■■■■■■■	777777777760771	T1	53	1	F/20		
PER01200310122	2003	0122	1	■■■■■■■■■■■■■■■■■■■■■■■■■■■■■■■■□□□□■■■■■■■	777777777760771	T1	53	1	F/50		
PER01200310123	2003	0123	1	■■■■■■■■■■■■■■■■■■■■■■■■■■■■■■■■□□□□■■■■■■■	777777777760771	T1	53	1	M/24		
PER01200310132	2003	0132	1	■■■■■■■■■■■■■■■■■■■■■■■■■■■■■■■■□□□□■■■■■■■	777777777760771	T1	53	1	F/21		
PER01200310133	2003	0133	1	■■■■■■■■■■■■■■■■■■■■■■■■■■■■■■■■□□□□■■■■■■■	777777777760771	T1	53	1	F/45		
PER01200320137	2003	0137	1	■■■■■■■■■■■■■■■■■■■■■■■■■■■■■■■■□□□□■■■■■■■	777777777760771	T1	53	2	M/23		
PER01200310152	2003	0152	1	■■■■■■■■■■■■■■■■■■■■■■■■■■■■■■■■□□□□■■■■■■■	777777777760771	T1	53	1	F/30		
PER01200410157	2004	0157	1	■■■■■■■■■■■■■■■■■■■■■■■■■■■■■■■■□□□□■■■■■■■	777777777760771	T1	53	1	F/21		
PER01200410208	2004	0208	1	■■■■■■■■■■■■■■■■■■■■■■■■■■■■■■■■□□□□■■■■■■■	777777777760771	T1	53	1	M/20		
PER01200410210	2004	0210	1	■■■■■■■■■■■■■■■■■■■■■■■■■■■■■■■■□□□□■■■■■■■	777777777760771	T1	53	1	M/26		
PER01200430215	2004	0215	1	■■■■■■■■■■■■■■■■■■■■■■■■■■■■■■■■□□□□■■■■■■■	777777777760771	T1	53	3	M/23	RIF resist	
PER01200420218	2004	0218	1	■■■■■■■■■■■■■■■■■■■■■■■■■■■■■■■■□□□□■■■■■■■	777777777760771	T1	53	2	F/34		
PER01200430219	2004	0219	1	■■■■■■■■■■■■■■■■■■■■■■■■■■■■■■■■□□□□■■■■■■■	777777777760771	T1	53	3	M/22	RIF resist	
PER01199920225	1999	0225	1	■■■■■■■■■■■■■■■■■■■■■■■■■■■■■■■■□□□□■■■■■■■	777777777760771	T1	53	2	F/30		HIV+
PER01199920226	1999	0226	1	■■■■■■■■■■■■■■■■■■■■■■■■■■■■■■■■□□□□■■■■■■■	777777777760771	T1	53	2	M/33		HIV+
PER01199920230	1999	0230	1	■■■■■■■■■■■■■■■■■■■■■■■■■■■■■■■■□□□□■■■■■■■	777777777760771	T1	53	2	M/34		HIV+
PER01199920231	1999	0231	1	■■■■■■■■■■■■■■■■■■■■■■■■■■■■■■■■□□□□■■■■■■■	777777777760771	T1	53	2	F/39		HIV+
PER01199920242	1999	0242	1	■■■■■■■■■■■■■■■■■■■■■■■■■■■■■■■■□□□□■■■■■■■	777777777760771	T1	53	2	M/30		HIV+
PER01199920247	1999	0247	1	■■■■■■■■■■■■■■■■■■■■■■■■■■■■■■■■□□□□■■■■■■■	777777777760771	T1	53	2	F/20		HIV+
PER01199920248	1999	0248	1	■■■■■■■■■■■■■■■■■■■■■■■■■■■■■■■■□□□□■■■■■■■	777777777760771	T1	53	2	M/53		HIV+
PER01199920250	1999	0250	1	■■■■■■■■■■■■■■■■■■■■■■■■■■■■■■■■□□□□■■■■■■■	777777777760771	T1	53	2	M/35		HIV+
PER01199910253	1999	0253	1	■■■■■■■■■■■■■■■■■■■■■■■■■■■■■■■■□□□□■■■■■■■	777777777760771	T1	53	1	M/32		HIV+
PER01199920256	1999	0256	1	■■■■■■■■■■■■■■■■■■■■■■■■■■■■■■■■□□□□■■■■■■■	777777777760771	T1	53	2	F/26		HIV+
PER01199910258	1999	0258	1	■■■■■■■■■■■■■■■■■■■■■■■■■■■■■■■■□□□□■■■■■■■	777777777760771	T1	53	1	M/55		HIV+
PER01199920261	1999	0261	1	■■■■■■■■■■■■■■■■■■■■■■■■■■■■■■■■□□□□■■■■■■■	777777777760771	T1	53	2	M/23		HIV+
PER01200020267	2000	0267	1	■■■■■■■■■■■■■■■■■■■■■■■■■■■■■■■■□□□□■■■■■■■	777777777760771	T1	53	2	M/45		HIV+
PER01200020268	2000	0268	1	■■■■■■■■■■■■■■■■■■■■■■■■■■■■■■■■□□□□■■■■■■■	777777777760771	T1	53	2	M/25		HIV+
PER01200020282	2000	0282	1	■■■■■■■■■■■■■■■■■■■■■■■■■■■■■■■■□□□□■■■■■■■	777777777760771	T1	53	2	M/24		HIV+
PER01200020297	2000	0297	1	■■■■■■■■■■■■■■■■■■■■■■■■■■■■■■■■□□□□■■■■■■■	777777777760771	T1	53	2	M/31		HIV+
PER01200120305	2001	0305	1	■■■■■■■■■■■■■■■■■■■■■■■■■■■■■■■■□□□□■■■■■■■	777777777760771	T1	53	2	M/31		HIV+
PER01200120307	2001	0307	1	■■■■■■■■■■■■■■■■■■■■■■■■■■■■■■■■□□□□■■■■■■■	777777777760771	T1	53	2	F/29		HIV+
PER01200120315	2001	0315	1	■■■■■■■■■■■■■■■■■■■■■■■■■■■■■■■■□□□□■■■■■■■	777777777760771	T1	53	2	F/33		HIV+
PER01200120319	2001	0319	1	■■■■■■■■■■■■■■■■■■■■■■■■■■■■■■■■□□□□■■■■■■■	777777777760771	T1	53	2	M/27		HIV+
PER01200120321	2001	0321	1	■■■■■■■■■■■■■■■■■■■■■■■■■■■■■■■■□□□□■■■■■■■	777777777760771	T1	53	2	M/32		HIV+
PER01200120324	2001	0324	1	■■■■■■■■■■■■■■■■■■■■■■■■■■■■■■■■□□□□■■■■■■■	777777777760771	T1	53	2	M/31		HIV+
PER01200130331	2001	0331	1	■■■■■■■■■■■■■■■■■■■■■■■■■■■■■■■■□□□□■■■■■■■	777777777760771	T1	53	3	M/42	INH resist	HIV+
PER01200220333	2002	0333	1	■■■■■■■■■■■■■■■■■■■■■■■■■■■■■■■■□□□□■■■■■■■	777777777760771	T1	53	2	M/37		HIV+
PER01199910356	1999	0356	1	■■■■■■■■■■■■■■■■■■■■■■■■■■■■■■■■□□□□■■■■■■■	777777777760771	T1	53	1	F/52		
PER01199920357	1999	0357	1	■■■■■■■■■■■■■■■■■■■■■■■■■■■■■■■■□□□□■■■■■■■	777777777760771	T1	53	2	M/0		
PER01199930379	1999	0379	1	■■■■■■■■■■■■■■■■■■■■■■■■■■■■■■■■□□□□■■■■■■■	777777777760771	T1	53	3	F/0	INH resist	

PER01199920380	1999	0380	1	■■■■■■■■■■■■■■■■■■■■■■■■■■■■■■■■□□□□■■■■■■■	777777777760771	T1	53	2	F/0		
PER01199910390	1999	0390	1	■■■■■■■■■■■■■■■■■■■■■■■■■■■■■■■■□□□□■■■■■■■	777777777760771	T1	53	1	F/0		
PER01199930398	1999	0398	1	■■■■■■■■■■■■■■■■■■■■■■■■■■■■■■■■□□□□■■■■■■■	777777777760771	T1	53	3	M/0	INH resist	
PER01199910401	1999	0401	1	■■■■■■■■■■■■■■■■■■■■■■■■■■■■■■■■□□□□■■■■■■■	777777777760771	T1	53	1	M/0		
PER01199920404	1999	0404	1	■■■■■■■■■■■■■■■■■■■■■■■■■■■■■■■■□□□□■■■■■■■	777777777760771	T1	53	2	F/28		
PER01199910407	1999	0407	1	■■■■■■■■■■■■■■■■■■■■■■■■■■■■■■■■□□□□■■■■■■■	777777777760771	T1	53	1	M/0		
PER01199910434	1999	0434	1	■■■■■■■■■■■■■■■■■■■■■■■■■■■■■■■■□□□□■■■■■■■	777777777760771	T1	53	1	F/0		
PER01199910437	1999	0437	1	■■■■■■■■■■■■■■■■■■■■■■■■■■■■■■■■□□□□■■■■■■■	777777777760771	T1	53	1	F/20		
PER01199910445	1999	0445	1	■■■■■■■■■■■■■■■■■■■■■■■■■■■■■■■■□□□□■■■■■■■	777777777760771	T1	53	1	M/0		
PER01199910448	1999	0448	1	■■■■■■■■■■■■■■■■■■■■■■■■■■■■■■■■□□□□■■■■■■■	777777777760771	T1	53	1	F/0		
PER01199910456	1999	0456	1	■■■■■■■■■■■■■■■■■■■■■■■■■■■■■■■■□□□□■■■■■■■	777777777760771	T1	53	1	M/0		
PER01199910464	1999	0464	1	■■■■■■■■■■■■■■■■■■■■■■■■■■■■■■■■□□□□■■■■■■■	777777777760771	T1	53	1	M/0		
PER01200010473	2000	0473	1	■■■■■■■■■■■■■■■■■■■■■■■■■■■■■■■■□□□□■■■■■■■	777777777760771	T1	53	1	F/0		
PER01200010474	2000	0474	1	■■■■■■■■■■■■■■■■■■■■■■■■■■■■■■■■□□□□■■■■■■■	777777777760771	T1	53	1	F/26		
PER01200010484	2000	0484	1	■■■■■■■■■■■■■■■■■■■■■■■■■■■■■■■■□□□□■■■■■■■	777777777760771	T1	53	1	M/25		
PER01200010505	2000	0505	1	■■■■■■■■■■■■■■■■■■■■■■■■■■■■■■■■□□□□■■■■■■■	777777777760771	T1	53	1	F/0		
PER01200010513	2000	0513	1	■■■■■■■■■■■■■■■■■■■■■■■■■■■■■■■■□□□□■■■■■■■	777777777760771	T1	53	1	F/21		
PER01200030521	2000	0521	1	■■■■■■■■■■■■■■■■■■■■■■■■■■■■■■■■□□□□■■■■■■■	777777777760771	T1	53	3	M/0	INH resist	
PER01200010538	2000	0538	1	■■■■■■■■■■■■■■■■■■■■■■■■■■■■■■■■□□□□■■■■■■■	777777777760771	T1	53	1	F/0		
PER01200010543	2000	0543	1	■■■■■■■■■■■■■■■■■■■■■■■■■■■■■■■■□□□□■■■■■■■	777777777760771	T1	53	1	M/0		
PER01200010546	2000	0546	1	■■■■■■■■■■■■■■■■■■■■■■■■■■■■■■■■□□□□■■■■■■■	777777777760771	T1	53	1	F/0		
PER01200010555	2000	0555	1	■■■■■■■■■■■■■■■■■■■■■■■■■■■■■■■■□□□□■■■■■■■	777777777760771	T1	53	1	M/0		
PER01200010575	2000	0575	1	■■■■■■■■■■■■■■■■■■■■■■■■■■■■■■■■□□□□■■■■■■■	777777777760771	T1	53	1	M/0		
PER01200010585	2000	0585	1	■■■■■■■■■■■■■■■■■■■■■■■■■■■■■■■■□□□□■■■■■■■	777777777760771	T1	53	1	F/0		
PER01200110588	2001	0588	1	■■■■■■■■■■■■■■■■■■■■■■■■■■■■■■■■□□□□■■■■■■■	777777777760771	T1	53	1	F/0		
PER01200110612	2001	0612	1	■■■■■■■■■■■■■■■■■■■■■■■■■■■■■■■■□□□□■■■■■■■	777777777760771	T1	53	1	M/0		
PER01200110616	2001	0616	1	■■■■■■■■■■■■■■■■■■■■■■■■■■■■■■■■□□□□■■■■■■■	777777777760771	T1	53	1	F/23		
PER01200110624	2001	0624	1	■■■■■■■■■■■■■■■■■■■■■■■■■■■■■■■■□□□□■■■■■■■	777777777760771	T1	53	1	M/0		
PER01200130625	2001	0625	1	■■■■■■■■■■■■■■■■■■■■■■■■■■■■■■■■□□□□■■■■■■■	777777777760771	T1	53	3	M/41	INH resist	
PER01200220653	2002	0653	1	■■■■■■■■■■■■■■■■■■■■■■■■■■■■■■■■□□□□■■■■■■■	777777777760771	T1	53	2	F/21		
PER01200210657	2002	0657	1	■■■■■■■■■■■■■■■■■■■■■■■■■■■■■■■■□□□□■■■■■■■	777777777760771	T1	53	1	F/31		
PER01200210665	2002	0665	1	■■■■■■■■■■■■■■■■■■■■■■■■■■■■■■■■□□□□■■■■■■■	777777777760771	T1	53	1	F/19		
PER01200210666	2002	0666	1	■■■■■■■■■■■■■■■■■■■■■■■■■■■■■■■■□□□□■■■■■■■	777777777760771	T1	53	1	F/25		
PER01200210673	2002	0673	1	■■■■■■■■■■■■■■■■■■■■■■■■■■■■■■■■□□□□■■■■■■■	777777777760771	T1	53	1	M/33		HIV+
PER01200210674	2002	0674	1	■■■■■■■■■■■■■■■■■■■■■■■■■■■■■■■■□□□□■■■■■■■	777777777760771	T1	53	1	M/42		HIV+
PER01200210676	2002	0676	1	■■■■■■■■■■■■■■■■■■■■■■■■■■■■■■■■□□□□■■■■■■■	777777777760771	T1	53	1	M/30		HIV+
PER01200220677	2002	0677	1	■■■■■■■■■■■■■■■■■■■■■■■■■■■■■■■■□□□□■■■■■■■	777777777760771	T1	53	2	F/34		HIV+
PER01200220682	2002	0682	1	■■■■■■■■■■■■■■■■■■■■■■■■■■■■■■■■□□□□■■■■■■■	777777777760771	T1	53	2	F/25		HIV+
PER01200210692	2002	0692	1	■■■■■■■■■■■■■■■■■■■■■■■■■■■■■■■■□□□□■■■■■■■	777777777760771	T1	53	1	0/0		HIV+
PER01200220693	2002	0693	1	■■■■■■■■■■■■■■■■■■■■■■■■■■■■■■■■□□□□■■■■■■■	777777777760771	T1	53	2	M/32		HIV+
PER01200210694	2002	0694	1	■■■■■■■■■■■■■■■■■■■■■■■■■■■■■■■■□□□□■■■■■■■	777777777760771	T1	53	1	M/22		HIV+
PER01200210706	2002	0706	1	■■■■■■■■■■■■■■■■■■■■■■■■■■■■■■■■□□□□■■■■■■■	777777777760771	T1	53	1	M/31		HIV+
PER01200230712	2002	0712	1	■■■■■■■■■■■■■■■■■■■■■■■■■■■■■■■■□□□□■■■■■■■	777777777760771	T1	53	3	F/44	RIF resist	HIV+
PER01200210716	2002	0716	1	■■■■■■■■■■■■■■■■■■■■■■■■■■■■■■■■□□□□■■■■■■■	777777777760771	T1	53	1	M/43		HIV+
PER01200330731	2003	0731	1	■■■■■■■■■■■■■■■■■■■■■■■■■■■■■■■■□□□□■■■■■■■	777777777760771	T1	53	3	M/22	INH resist	HIV+
PER01200310739	2003	0739	1	■■■■■■■■■■■■■■■■■■■■■■■■■■■■■■■■□□□□■■■■■■■	777777777760771	T1	53	1	F/34		HIV+
PER01200310760	2003	0760	1	■■■■■■■■■■■■■■■■■■■■■■■■■■■■■■■■□□□□■■■■■■■	777777777760771	T1	53	1	F/47		HIV+
PER01200310767	2003	0767	1	■■■■■■■■■■■■■■■■■■■■■■■■■■■■■■■■□□□□■■■■■■■	777777777760771	T1	53	1	M/32		HIV+
PER01200410794	2004	0794	1	■■■■■■■■■■■■■■■■■■■■■■■■■■■■■■■■□□□□■■■■■■■	777777777760771	T1	53	1	M/32		HIV+
PER01200410795	2004	0795	1	■■■■■■■■■■■■■■■■■■■■■■■■■■■■■■■■□□□□■■■■■■■	777777777760771	T1	53	1	M/27		HIV+
PER01200330124	2003	0124	1	■■■■■■■■■■■■■■■■■■■■■■■■■■■■■■■■□□■■■■■■■■■	777777777763771	MANU2	54	3	F/58	INH resist	

PER01199930229	1999	0229	1	■■■■■■■■■■■■■■■■■■■□■■□■■■■■■■■■■□□□□■■■■■■■	777777557760771	T5-Madrid2	58	3	M/38	RIF resist	HIV+
PER01199910254	1999	0254	1	■■■■■■■■■■■■■■■■■■■□■■□■■■■■■■■■■□□□□■■■■■■■	777777557760771	T5-Madrid2	58	1	M/24		HIV+
PER01199930376	1999	0376	1	■■■■■■■■■■■■■■■■■■■□■■□■■■■■■■■■■□□□□■■■■■■■	777777557760771	T5-Madrid2	58	3	M/0	RIF resist	
PER01199910411	1999	0411	1	■■■■■■■■■■■■■■■■■■■□■■□■■■■■■■■■■□□□□■■■■■■■	777777557760771	T5-Madrid2	58	1	M/0		
PER01200010545	2000	0545	1	■■■■■■■■■■■■■■■■■■■□■■□■■■■■■■■■■□□□□■■■■■■■	777777557760771	T5-Madrid2	58	1	M/32		
PER01200010565	2000	0565	1	■■■■■■■■■■■■■■■■■■■□■■□■■■■■■■■■■□□□□■■■■■■■	777777557760771	T5-Madrid2	58	1	M/0		
PER01200110638	2001	0638	1	■■■■■■■■■■■■■■■■■■■□■■□■■■■■■■■■■□□□□■■■■■■■	777777557760771	T5-Madrid2	58	1	F/22		
PER01200210715	2002	0715	1	■■■■■■■■■■■■■■■■■■■□■■□■■■■■■■■■■□□□□■■■■■■■	777777557760771	T5-Madrid2	58	1	M/32		HIV+
PER01200420035	2004	0035	1	■■■■■■■■■■■■■■■■■■■■□□□□■■■■■■■■□□□□■■■□■■■	777777607760731	LAM4	60	2	M/23		
PER01200210698	2002	0698	1	■■■■■■■■■■■■■■■■■■■■□□□□■■■■■■■■□□□□■■■□■■■	777777607760731	LAM4	60	1	M/48		HIV+
PER01200410199	2004	0199	1	■■■■■■■■■■■■■■■■■■■■■■■■■□□□□□□■□□□□■■■□■■■	777777774020731	H1	62	1	F/25		
PER01199930234	1999	0234	1	■■■■■■■■■■■■■■■■■■■■□□□□■■■■□■■■□□□□■■■■■■■	777777607560771	LAM6	64	3	M/30	RIF resist	HIV+
PER01199910396	1999	0396	1	■■■■■■■■■■■■■■■■■■■■□□□□■■■■□■■■□□□□■■■■■■■	777777607560771	LAM6	64	1	F/0		
PER01200010503	2000	0503	1	■■■■■■■■■■■■■■■■■■■■□□□□■■■■□■■■□□□□■■■■■■■	777777607560771	LAM6	64	1	F/0		
PER01200010564	2000	0564	1	■■■■■■■■■■■■■■■■■■■■□□□□■■■■□■■■□□□□■■■■■■■	777777607560771	LAM6	64	1	M/0		
PER01200110628	2001	0628	1	■■■■■■■■■■■■■■■■■■■■□□□□■■■■□■■■□□□□■■■■■■■	777777607560771	LAM6	64	1	M/0		
PER01200310766	2003	0766	1	■■■■■■■■■■■■■■■■■■■■□□□□■■■■□■■■□□□□■■■■■■■	777777607560771	LAM6	64	1	M/31		HIV+
PER01199910465	1999	465	1	■■■■■■■■■■■■□■■■■■■■■■■■■■■■■■■■□□□□■■■□■■■	777737777760731	T	73	1	M/33		
PER01200520799	2005	0799	1	■■■■■■■■■■■■□■■■■■■■■■■■■■■■■■■■□□□□■■■□■■■	777737777760731	T	73	2	M/33		HIV+
PER01200310762	2003	0762	1	■■■■■■■■■■■■■■■■■■■■■■■■■■■■■■■■□□□□■■■□□■■	777777777760711	T	78	1	M/33		HIV+
PER01200420028	2004	0028	1	■■■■■■■■■■■■■■■■■■■■■□■■■■■■■■■■■■□□□□■■■■■■■	777777737760771	T1	86	2	M/20		
PER01200510040	2005	0040	1	■■■□□□□□□□□□□■■■■□■■■■■■■■■■■■■■□□□□■■■■■■■	700036777760771	X3	91	1	F/40		
PER01200510047	2005	0047	1	■■■□□□□□□□□□□■■■■□■■■■■■■■■■■■■■□□□□■■■■■■■	700036777760771	X3	91	1	M/26		
PER01200510051	2005	0051	1	■■■□□□□□□□□□□■■■■□■■■■■■■■■■■■■■□□□□■■■■■■■	700036777760771	X3	91	1	M/45		
PER01200310121	2003	0121	1	■■■□□□□□□□□□□■■■■□■■■■■■■■■■■■■■□□□□■■■■■■■	700036777760771	X3	91	1	F/23		
PER01200410161	2004	0161	1	■■■□□□□□□□□□□■■■■□■■■■■■■■■■■■■■□□□□■■■■■■■	700036777760771	X3	91	1	M/25		
PER01200420170	2004	0170	1	■■■□□□□□□□□□□■■■■□■■■■■■■■■■■■■■□□□□■■■■■■■	700036777760771	X3	91	2	F/44		
PER01199910249	1999	0249	1	■■■□□□□□□□□□□■■■■□■■■■■■■■■■■■■■□□□□■■■■■■■	700036777760771	X3	91	1	M/36		HIV+
PER01199910251	1999	0251	1	■■■□□□□□□□□□□■■■■□■■■■■■■■■■■■■■□□□□■■■■■■■	700036777760771	X3	91	1	F/41		HIV+
PER01199910397	1999	0397	1	■■■□□□□□□□□□□■■■■□■■■■■■■■■■■■■■□□□□■■■■■■■	700036777760771	X3	91	1	F/0		
PER01199930414	1999	0414	1	■■■□□□□□□□□□□■■■■□■■■■■■■■■■■■■■□□□□■■■■■■■	700036777760771	X3	91	3	M/0	INH resist	
PER01199910428	1999	0428	1	■■■□□□□□□□□□□■■■■□■■■■■■■■■■■■■■□□□□■■■■■■■	700036777760771	X3	91	1	M/0		
PER01199910459	1999	0459	1	■■■□□□□□□□□□□■■■■□■■■■■■■■■■■■■■□□□□■■■■■■■	700036777760771	X3	91	1	M/0		
PER01200010486	2000	0486	1	■■■□□□□□□□□□□■■■■□■■■■■■■■■■■■■■□□□□■■■■■■■	700036777760771	X3	91	1	M/24		
PER01200010512	2000	0512	1	■■■□□□□□□□□□□■■■■□■■■■■■■■■■■■■■□□□□■■■■■■■	700036777760771	X3	91	1	F/39		
PER01200010527	2000	0527	1	■■■□□□□□□□□□□■■■■□■■■■■■■■■■■■■■□□□□■■■■■■■	700036777760771	X3	91	1	M/0		
PER01200010560	2000	0560	1	■■■□□□□□□□□□□■■■■□■■■■■■■■■■■■■■□□□□■■■■■■■	700036777760771	X3	91	1	M/30		
PER01200110644	2001	0644	1	■■■□□□□□□□□□□■■■■□■■■■■■■■■■■■■■□□□□■■■■■■■	700036777760771	X3	91	1	F/20		
PER01200220702	2002	0702	1	■■■□□□□□□□□□□■■■■□■■■■■■■■■■■■■■□□□□■■■■■■■	700036777760771	X3	91	2	M/31		HIV+
PER01200330735	2003	0735	1	■■■□□□□□□□□□□■■■■□■■■■■■■■■■■■■■□□□□■■■■■■■	700036777760771	X3	91	3	M/39	INH resist	HIV+
PER01200310753	2003	0753	1	■■■□□□□□□□□□□■■■■□■■■■■■■■■■■■■■□□□□■■■■■■■	700036777760771	X3	91	1	F/28		HIV+
PER01200310765	2003	0765	1	■■■□□□□□□□□□□■■■■□■■■■■■■■■■■■■■□□□□■■■■■■■	700036777760771	X3	91	1	M/26		HIV+
PER01200410778	2004	0778	1	■■■□□□□□□□□□□■■■■□■■■■■■■■■■■■■■□□□□■■■■■■■	700036777760771	X3	91	1	M/45		HIV+
PER01200410032	2004	0032	1	■■■□□□□□□□□□■■■■■□■■■■■■■■■■■■■■□□□□■■■■■■■	700076777760771	X3	92	1	F/18		
PER01200410033	2004	0033	1	■■■□□□□□□□□□■■■■■□■■■■■■■■■■■■■■□□□□■■■■■■■	700076777760771	X3	92	1	F/20		
PER01200510059	2005	0059	1	■■■□□□□□□□□□■■■■■□■■■■■■■■■■■■■■□□□□■■■■■■■	700076777760771	X3	92	1	M/18		
PER01200510060	2005	0060	1	■■■□□□□□□□□□■■■■■□■■■■■■■■■■■■■■□□□□■■■■■■■	700076777760771	X3	92	1	M/21		
PER01200010516	2000	0516	1	■■■□□□□□□□□□■■■■■□■■■■■■■■■■■■■■□□□□■■■■■■■	700076777760771	X3	92	1	M/0		
PER01200530045	2005	0045	1	■■■■■■■■■■■■□■■■■■■■□□□□■■■■■■■■□□□□■■■■■■■	777737607760771	LAM5	93	3	F/38	INH resist	
PER01200020269	2000	0269	1	■■■■■■■■■■■■□■■■■■■■□□□□■■■■■■■■□□□□■■■■■■■	777737607760771	LAM5	93	2	M/30		HIV+
PER01199910352	1999	0352	1	■■■■■■■■■■■■□■■■■■■■□□□□■■■■■■■■□□□□■■■■■■■	777737607760771	LAM5	93	1	M/0		

PER01199920370	1999	0370	1	■■■■■■■■■■■■□■■■■■■■■□□□□■■■■■■■■□□□□■■■■■■■	777737607760771	LAM5	93	2	M/0		
PER01199910382	1999	0382	1	■■■■■■■■■■■■□■■■■■■■■□□□□■■■■■■■■□□□□■■■■■■■	777737607760771	LAM5	93	1	M/0		
PER01199930446	1999	0446	1	■■■■■■■■■■■■□■■■■■■■■□□□□■■■■■■■■□□□□■■■■■■■	777737607760771	LAM5	93	3	M/0	INH resist	
PER01199930450	1999	0450	1	■■■■■■■■■■■■□■■■■■■■■□□□□■■■■■■■■□□□□■■■■■■■	777737607760771	LAM5	93	3	F/0	INH resist	
PER01200220660	2002	0660	1	■■■■■■■■■■■■□■■■■■■■■□□□□■■■■■■■■□□□□■■■■■■■	777737607760771	LAM5	93	2	M/17		
PER01200220697	2002	0697	1	■■■■■■■■■■■■□■■■■■■■■□□□□■■■■■■■■□□□□■■■■■■■	777737607760771	LAM5	93	2	M/32		HIV+
PER01200220714	2002	0714	1	■■■■■■■■■■■■□■■■■■■■■□□□□■■■■■■■■□□□□■■■■■■■	777737607760771	LAM5	93	2	M/40		HIV+
PER01200310726	2003	0726	1	■■■■■■■■■■■■□■■■■■■■■□□□□■■■■■■■■□□□□■■■■■■■	777737607760771	LAM5	93	1	M/34		HIV+
PER01200310747	2003	0747	1	■■■■■■■■■■■■□■■■■■■■■□□□□■■■■■■■■□□□□■■■■■■■	777737607760771	LAM5	93	1	M/42		HIV+
PER01200030273	2000	0273	1	■■■■■■■■■■■■■■■■■■■■□□□□■■■■□■■■□□□□■■■□■■■	777777607560731	LAM6	95	3	F/38	RIF resist	HIV+
PER01199910386	1999	0386	1	■■■■■■■■■■■■■■■■■■■■□□□□■■■■□■■■□□□□■■■□■■■	777777607560731	LAM6	95	1	M/55		
PER01199910394	1999	0394	1	■■■■□■■■■■■■■■■■■■■■■■■■■■■■□■□□□□■■■■■■■	757777777720771	H3	99	1	F/0		
PER01200110647	2001	0647	1	■■■■□■■■■■■■■■■■■■■■■■■■■■■■□■□□□□■■■■■■■	757777777720771	H3	99	1	F/50		
PER01199910232	1999	0232	1	■■■■■■■■□□□■■■■■■■■□□□□□□□□□□□□□□□□□□□■■■■■	776177400000171	Unknown	106	1	M/38		HIV+
PER01199910374	1999	0374	1	■■■■■■■■□□□■■■■■■■■□□□□□□□□□□□□□□□□□□□■■■■■	776177400000171	Unknown	106	1	F/0		
PER01200310154	2003	0154	1	■■■■■■■■■■■■■■■■■□■■■■■■■■■■■■■■□□□□■■■■■■■	777776777760771	X1	119	1	M/30		
PER01200310155	2003	0155	1	■■■■■■■■■■■■■■■■■□■■■■■■■■■■■■■■□□□□■■■■■■■	777776777760771	X1	119	1	F/33		
PER01200310763	2003	0763	1	■■■■■■■■■■■■■■■■■□■■■■■■■■■■■■■■□□□□■■■■■■■	777776777760771	X1	119	1	M/26		HIV+
PER01200410781	2004	0781	1	■■■■■■■■■■■■■■■■■□■■■■■■■■■■■■■■□□□□■■■■■■■	777776777760771	X1	119	1	M/39		HIV+
PER01200430026	2004	0026	1	■■■■■■■■□□□■■■■■■■■■□□□□■■■■■■■■□□□□■■■□■■■	776177607760731	LAM3	130	3	F/68	INH resist	
PER01200310103	2003	0103	1	■■■■■■■■□□□■■■■■■■■■□□□□■■■■■■■■□□□□■■■□■■■	776177607760731	LAM3	130	1	F/32		
PER01199910392	1999	0392	1	■■■■■■■■□□□■■■■■■■■■□□□□■■■■■■■■□□□□■■■□■■■	776177607760731	LAM3	130	1	F/0		
PER01200020541	2000	0541	1	■■■■■■■■□□□■■■■■■■■■□□□□■■■■■■■■□□□□■■■□■■■	776177607760731	LAM3	130	2	M/31		
PER01200110640	2001	0640	1	■■■■■■■■□□□■■■■■■■■■□□□□■■■■■■■■□□□□■■■□■■■	776177607760731	LAM3	130	1	F/31		
PER01200210691	2002	0691	1	■■■■■■■■□□□■■■■■■■■■□□□□■■■■■■■■□□□□■■■□■■■	776177607760731	LAM3	130	1	0/0		HIV+
PER01200310742	2003	0742	1	■■■■■■■■□□□■■■■■■■■■□□□□■■■■■■■■□□□□■■■□■■■	776177607760731	LAM3	130	1	F/26		HIV+
PER01200430182	2004	0182	1	■■■■■■■■■■■■■■■■■■■■□□□□■■□□□□□□□□□□□□□□■■■	777777606000031	Unknown	132	3	F/24	INH resist	
PER01200410184	2004	0184	1	□■■■■■■■■■■■■■■■■■■■□□□□■■■■■■■■□□□□■■■■■■■	377777607760771	LAM9	177	1	M/71		
PER01199910426	1999	0426	1	■■■■■■■■■■■■■■■■■■□■■■■■■■■■■■■□■□□□□■■■■■■■	777777377720771	H3	183	1	M/0		
PER01200010482	2000	0482	1	■■■■■■■■■■■■■■■■■■□■■■■■■■■■■■■□■□□□□■■■■■■■	777777377720771	H3	183	1	F/0		
PER01200010489	2000	0489	1	■■■■■■■■■■■■■■■■■■□■■■■■■■■■■■■□■□□□□■■■■■■■	777777377720771	H3	183	1	M/0		
PER01200130601	2001	0601	1	□□□□□□□□□□□□□□□□□□□□□□□□□□□□□□□□□□■■■■■□■■■	000000000003731	Beijing	190	3	M/0	INH resist	
PER01200310084	2003	0084	1	■■■■■■■■□□□■□■■■■■■■□□□□■■■■■■■■□□□□■■■■■■■	776137607760771	LAM3	211	1	M/24		
PER01199910353	1999	0353	1	■■■■■■■■■■■■□□□□■■■■■■■■■■■□■■■■□□□□■■■■■■■	777703777360771	T	215	1	F/0		
PER01200030481	2000	0481	1	■■■■■■■■■■■■□□■■■■■■□□□□■■■■■■■■□□□□■■■■■■■	777717607760771	LAM5	216	3	M/0	RIF resist	
PER01200010490	2000	0490	1	■■■■■■■■■■■■□□■■■■■■□□□□■■■■■■■■□□□□■■■■■■■	777717607760771	LAM5	216	1	M/0		
PER01200010572	2000	0572	1	■■■■■■■■■■■■□□■■■■■■□□□□■■■■■■■■□□□□■■■■■■■	777717607760771	LAM5	216	1	F/0		
PER01200320089	2003	0089	1	■■■■■■■■■■■■■□□□□□■■■■■■■■■■■■■■□□□□■■■■■■■	777740777760771	T1	219	2	M/23		
PER01200030286	2000	0286	1	■■■■■■■■■■■■■□□□□□■■■■■■■■■■■■■■□□□□■■■■■■■	777740777760771	T1	219	3	M/40	INH resist	HIV+
PER01199920389	1999	0389	1	■■■■■■■■■■■■■□□□□□■■■■■■■■■■■■■■□□□□■■■■■■■	777740777760771	T1	219	2	M/0		
PER01199930417	1999	0417	1	■■■■■■■■■■■■■□□□□□■■■■■■■■■■■■■■□□□□■■■■■■■	777740777760771	T1	219	3	M/0	INH resist	
PER01199920429	1999	0429	1	■■■■■■■■■■■■■□□□□□■■■■■■■■■■■■■■□□□□■■■■■■■	777740777760771	T1	219	2	M/0		
PER01200410037	2004	0037	1	■■■■■■■■■■■■■■■■□□□□□■■■■■■■□■■■□□□□■■■■■■■	777774077560771	Unknown	222	1	M/23		
PER01200510057	2005	0057	1	■■■■■■■■■■■■■■■■□□□□□■■■■■■■□■■■□□□□■■■■■■■	777774077560771	Unknown	222	1	M/44		
PER01200530067	2005	0067	1	■■■■■■■■■■■■■■■■□□□□□■■■■■■■□■■■□□□□■■■■■■■	777774077560771	Unknown	222	3	F/30	INH resist	
PER01200530068	2005	0068	1	■■■■■■■■■■■■■■■■□□□□□■■■■■■■□■■■□□□□■■■■■■■	777774077560771	Unknown	222	3	F/25	INH resist	
PER01200320076	2003	0076	1	■■■■■■■■■■■■■■■■□□□□□■■■■■■■□■■■□□□□■■■■■■■	777774077560771	Unknown	222	2	M/63		
PER01200430188	2004	0188	1	■■■■■■■■■■■■■■■■□□□□□■■■■■■■□■■■□□□□■■■■■■■	777774077560771	Unknown	222	3	M/24	INH resist	
PER01200430213	2004	0213	1	■■■■■■■■■■■■■■■■□□□□□■■■■■■■□■■■□□□□■■■■■■■	777774077560771	Unknown	222	3	M/25	RIF resist	
PER01199910262	1999	0262	1	■■■■■■■■■■■■■■■■□□□□□■■■■■■■□■■■□□□□■■■■■■■	777774077560771	Unknown	222	1	F/42		HIV+
PER01200010284	2000	0284	1	■■■■■■■■■■■■■■■■□□□□□■■■■■■■□■■■□□□□■■■■■■■	777774077560771	Unknown	222	1	M/34		HIV+

PER01200020289	2000	0289	1	■■■■■■■■■■■■■■■■□□□□□■■■■■■■□■■■□□□□■■■■■■■	777774077560771	Unknown	222	2	M/29		HIV+
PER01200110312	2001	0312	1	■■■■■■■■■■■■■■■■□□□□□■■■■■■■□■■■□□□□■■■■■■■	777774077560771	Unknown	222	1	M/30		HIV+
PER01199910341	1999	0341	1	■■■■■■■■■■■■■■■■□□□□□■■■■■■■□■■■□□□□■■■■■■■	777774077560771	Unknown	222	1	M/0		
PER01199910355	1999	0355	1	■■■■■■■■■■■■■■■■□□□□□■■■■■■■□■■■□□□□■■■■■■■	777774077560771	Unknown	222	1	F/0		
PER01199910454	1999	0454	1	■■■■■■■■■■■■■■■■□□□□□■■■■■■■□■■■□□□□■■■■■■■	777774077560771	Unknown	222	1	M/21		
PER01200010471	2000	0471	1	■■■■■■■■■■■■■■■■□□□□□■■■■■■■□■■■□□□□■■■■■■■	777774077560771	Unknown	222	1	M/0		
PER01200030495	2000	0495	1	■■■■■■■■■■■■■■■■□□□□□■■■■■■■□■■■□□□□■■■■■■■	777774077560771	Unknown	222	3	M/0	RIF resist	
PER01200010500	2000	0500	1	■■■■■■■■■■■■■■■■□□□□□■■■■■■■□■■■□□□□■■■■■■■	777774077560771	Unknown	222	1	M/0		
PER01200010540	2000	0540	1	■■■■■■■■■■■■■■■■□□□□□■■■■■■■□■■■□□□□■■■■■■■	777774077560771	Unknown	222	1	M/0		
PER01200030569	2000	0569	1	■■■■■■■■■■■■■■■■□□□□□■■■■■■■□■■■□□□□■■■■■■■	777774077560771	Unknown	222	3	F/0	INH resist	
PER01200110632	2001	0632	1	■■■■■■■■■■■■■■■■□□□□□■■■■■■■□■■■□□□□■■■■■■■	777774077560771	Unknown	222	1	F/0		
PER01200110642	2001	0642	1	■■■■■■■■■■■■■■■■□□□□□■■■■■■■□■■■□□□□■■■■■■■	777774077560771	Unknown	222	1	M/35		
PER01200120646	2001	0646	1	■■■■■■■■■■■■■■■■□□□□□■■■■■■■□■■■□□□□■■■■■■■	777774077560771	Unknown	222	2	M/18		
PER01200220709	2002	0709	1	■■■■■■■■■■■■■■■■□□□□□■■■■■■■□■■■□□□□■■■■■■■	777774077560771	Unknown	222	2	M/23		HIV+
PER01200310721	2003	0721	1	■■■■■■■■■■■■■■■■□□□□□■■■■■■■□■■■□□□□■■■■■■■	777774077560771	Unknown	222	1	M/29		HIV+
PER01199910359	1999	0359	1	■■■■■■■■■■■■■■■■■■■■■■■■■■■■■■□□□□□□□□□□□□□	777777777700000	Unknown	237	1	M/0		
PER01200110623	2001	0623	1	■■■■■■■■■■■■■■■■■■■■■■■■■■■■■■□□□□□□□□□□□□□	777777777700000	Unknown	237	1	M/0		
PER01200210675	2002	0675	1	■■■■■■■■■■■■■■■■■■■■■■■■■■■■■■□□□□□□□□□□□□□	777777777700000	Unknown	237	1	M/25		HIV+
PER01200210703	2002	0703	1	■■■■■■■■■■■■■■■■■■■■■■■■■■■■■■□□□□□□□□□□□□□	777777777700000	Unknown	237	1	F/30		HIV+
PER01200320008	2003	0008	1	■■■■■■■■■■■■■■■■■■■■■■■■■■■■■■■■□□□□□□□□■■■	777777777760031	T2	239	2	M/28		
PER01200120326	2001	0326	1	■■■■■■■■■■■■■■■■■■■■■■■■■■■■■■■■□□□□□□□□■■■	777777777760031	T2	239	2	M/27		HIV+
PER01200310736	2003	0736	1	■■■■■■■■■■■■■■■■■■■■■□□□■□□□□□□■□□□□■■■■■■■	777777704020771	H1	283	1	M/21		HIV+
PER01200310117	2003	0117	1	■■■■■■■■■■■■■■■■■■■■■■■□■■■■■■■■□□□□■■■■■■■	777777767760771	T1	373	1	F/49		
PER01199910377	1999	0377	1	■■■■■■■■■■■■■■■■■■■■■■■□■■■■■■■■□□□□■■■■■■■	777777767760771	T1	373	1	F/0		
PER01200310153	2003	0153	1	■■■■■■■■■■■■■■■■■■■■□■■■■□□□□□□■□□□□■■■■■■■	777777674020771	H1	384	1	F/24		
PER01200310092	2003	0092	1	■■■■■■■■■■■■■■■■■■■■■■■■■■■■■□□■□□□□■■■■■■■	777777777620771	H3	390	1	F/20		
PER01199910444	1999	0444	1	■■■■■■■■■■■■■■■■■■■■■■■■■■■■■□□■□□□□■■■■■■■	777777777620771	H3	390	1	F/33		
PER01199910470	1999	0470	1	■■■■■■■■■■■■■■■■■■■■■■■■■■■■■□□■□□□□■■■■■■■	777777777620771	H3	390	1	M/0		
PER01200430223	2004	0223	1	■■■■■■■■■■■■■■■■■■■■□□□□■■■■□■■■□□□□□■■■■■■	777777607560371	LAM6	396	3	M/53	RIF resist	
PER01199910344	1999	0344	1	■■■■■■■■■■■■■■■■■■■■□□□□■■■■□■■■□□□□□■■■■■■	777777607560371	LAM6	396	1	M/0		
PER01200310071	2003	0071	1	■■□□□■■■■■■■■■■■■■■■■■■■■■■■■■□■□□□□■■■■■■■	617777777720771	H3	418	1	F/30		
PER01200310083	2003	0083	1	■■□□□■■■■■■■■■■■■■■■■■■■■■■■■■□■□□□□■■■■■■■	617777777720771	H3	418	1	M/18		
PER01199910240	1999	0240	1	■■□□□■■■■■■■■■■■■■■■■■■■■■■■■■□■□□□□■■■■■■■	617777777720771	H3	418	1	M/34		HIV+
PER01199910412	1999	0412	1	■■□□□■■■■■■■■■■■■■■■■■■■■■■■■■□■□□□□■■■■■■■	617777777720771	H3	418	1	F/0		
PER01199910442	1999	0442	1	■■□□□■■■■■■■■■■■■■■■■■■■■■■■■■□■□□□□■■■■■■■	617777777720771	H3	418	1	F/0		
PER01199910466	1999	0466	1	■■□□□■■■■■■■■■■■■■■■■■■■■■■■■■□■□□□□■■■■■■■	617777777720771	H3	418	1	M/0		
PER01200110620	2001	0620	1	■■□□□■■■■■■■■■■■■■■■■■■■■■■■■■□■□□□□■■■■■■■	617777777720771	H3	418	1	M/0		
PER01200330755	2003	0755	1	■■□□□■■■■■■■■■■■■■■■■■■■■■■■■■□■□□□□■■■■■■■	617777777720771	H3	418	3	M/32	RIF resist	HIV+
PER01200330758	2003	0758	1	■■□□□■■■■■■■■■■■■■■■■■■■■■■■■■□■□□□□■■■■■■■	617777777720771	H3	418	3	F/32	RIF resist	HIV+
PER01200010275	2000	0275	1	■■■□□□■□□□□□□□□□■■□■■■□□■■■■■■■■□□□□■□□■■■■	704003347760471	T4-CEU1	430	1	M/25		HIV+
PER01200010499	2000	0499	1	■■■□□□■□□□□□□□□□■■□■■■□□■■■■■■■■□□□□■□□■■■■	704003347760471	T4-CEU1	430	1	F/0		
PER01200110602	2001	0602	1	■■■□□□■□□□□□□□□□■■□■■■□□■■■■■■■■□□□□■□□■■■■	704003347760471	T4-CEU1	430	1	M/0		
PER01200310722	2003	0722	1	■■■□□□■□□□□□□□□□■■□■■■□□■■■■■■■■□□□□■□□■■■■	704003347760471	T4-CEU1	430	1	F/31		HIV+
PER01200530041	2005	0041	1	■■■■■■■■■■■■■■■■■□■■■■■■□□□□□□□□□□□□□□□□□□□	777776770000000	Unknown	450	3	M/22	INH resist	
PER01200310078	2003	0078	1	■■■■■■■■■■■■■■■■■□■■■■■■□□□□□□□□□□□□□□□□□□□	777776770000000	Unknown	450	1	M/29		
PER01200310146	2003	0146	1	■■■■■■■■■■■■■■■■■□■■■■■■□□□□□□□□□□□□□□□□□□□	777776770000000	Unknown	450	1	F/42		
PER01200310147	2003	0147	1	■■■■■■■■■■■■■■■■■□■■■■■■□□□□□□□□□□□□□□□□□□□	777776770000000	Unknown	450	1	F/45		
PER01200310150	2003	0150	1	■■■■■■■■■■■■■■■■■□■■■■■■□□□□□□□□□□□□□□□□□□□	777776770000000	Unknown	450	1	F/43		
PER01200410187	2004	0187	1	■■■■■■■■■■■■■■■■■□■■■■■■□□□□□□□□□□□□□□□□□□□	777776770000000	Unknown	450	1	M/23		
PER01200010285	2000	0285	1	■■■■■■■■■■■■■■■■■□■■■■■■□□□□□□□□□□□□□□□□□□□	777776770000000	Unknown	450	1	M/28		HIV+
PER01200110328	2001	0328	1	■■■■■■■■■■■■■■■■■□■■■■■■□□□□□□□□□□□□□□□□□□□	777776770000000	Unknown	450	1	F/23		HIV+

PER01199910381	1999	0381	1	■■■■■■■■■■■■■■■■■□■■■■■■□□□□□□□□□□□□□□□□□□□	777776770000000	Unknown	450	1	F/0		
PER01199930405	1999	0405	1	■■■■■■■■■■■■■■■■■□■■■■■■□□□□□□□□□□□□□□□□□□□	777776770000000	Unknown	450	3	M/0	RIF resist	
PER01199930440	1999	0440	1	■■■■■■■■■■■■■■■■■□■■■■■■□□□□□□□□□□□□□□□□□□□	777776770000000	Unknown	450	3	M/0	INH resist	
PER01200010549	2000	0549	1	■■■■■■■■■■■■■■■■■□■■■■■■□□□□□□□□□□□□□□□□□□□	777776770000000	Unknown	450	1	M/0		
PER01200130590	2001	0590	1	■■■■■■■■■■■■■■■■■□■■■■■■□□□□□□□□□□□□□□□□□□□	777776770000000	Unknown	450	3	M/0	RIF resist	
PER01200110648	2001	0648	1	■■■■■■■■■■■■■■■■■□■■■■■■□□□□□□□□□□□□□□□□□□□	777776770000000	Unknown	450	1	F/30		
PER01200320003	2003	0003	1	□□□■■■■■■■■■■■■■■■■■□□□□■■■■■■■■□□□□■■■■■■■	077777607760771	LAM1	469	2	M/28		
PER01200410167	2004	0167	1	■■■■■□■■■■■■■■■■■□■■■■■■■■■■■■■■□□□□■■□□□□■	767776777760601	X2	489	1	M/21		
PER01200310074	2003	0074	1	■■■■■■■■■■■■■■■■■■■■■□□□■■■■■■□■□□□□■■■■■■■	777777707720771	H3	512	1	M/19		
PER01200010485	2000	0485	1	■■■■■■■■■■■■■■■■■■■■■□□□■■■■■■□■□□□□■■■■■■■	777777707720771	H3	512	1	F/49		
PER01200330720	2003	0720	1	■■■■■■■■■■■■■■■■■■■■■□□□■■■■■■□■□□□□■■■■■■■	777777707720771	H3	512	3	M/38	RIF resist	HIV+
PER01200310728	2003	0728	1	■■■■■■■■■■■■■■■■■■■■■□□□■■■■■■□■□□□□■■■■■■■	777777707720771	H3	512	1	M/52		HIV+
PER01200410029	2004	0029	1	■■■■■■■■■■■■■■■■■■■■□□□□■■■■□□□□□□□□□□□□□□□	777777607400000	LAM	534	1	F/33		
PER01200410777	2004	0777	1	■■■□□□□□□□□□□■■■■□■■■■■■■■■■□■■■□□□□■■■■■■■	700036777560771	X3	546	1	M/63		HIV+
PER01199910461	1999	0461	1	■■■■■■■■□□■■■■■■■■■■■■■■□□□■■■■■□□□□■■■■■■■	776377770760771	S	559	1	M/0		
PER01200310114	2003	0114	1	■■■■■■■■■■■■■■■■■■■■■■□□■□□□□□□■□□□□■■■■■■■	777777744020771	H1	620	1	F/19		
PER01200410195	2004	0195	1	■■■■■■■■■■■■■■■■■■■■■■□□■□□□□□□■□□□□■■■■■■■	777777744020771	H1	620	1	F/41		
PER01200210687	2002	0687	1	□■□□□□□□□□□□□□□□■■□■■■■■■■■□■□□□□■■■■■□□□□□	200003377207600	BOV_4-CAPRAE	644	1	M/38		HIV+
PER01199910345	1999	0345	1	■■■■■■■■■■■■■■■■■■■■■■□□■■■■■■□■□□□□■■■■■■■	777777747720771	H3	740	1	M/0		
PER01200210661	2002	0661	1	■■■■■■■■■■■■■■■■■■■■■■■■■■■■■■□■□□□□■■■■■□□	777777777720760	H3	748	1	M/25		
PER01199910427	1999	0427	1	■■■■■■■■■■■■■■■■■■■■■■■■■■■■□□□■□□□□■■■■■■■	777777777420771	H3	777	1	F/0		
PER01200510066	2005	0066	1	■■■■■■■■□□■■■■■■■■■■■■■■■■■■■■■■□□□□■■■□■■■	776377777760731	S	784	1	F/29		
PER01200310106	2003	0106	1	■■■■■■■■■■■■■■■■■■■■■■■□□□□□□□□□□□□□□□□□□□□	777777760000000	Unknown	786	1	M/29		
PER01200310116	2003	0116	1	■■■■■■■■■■■■■■■■■■■■■■■□□□□□□□□□□□□□□□□□□□□	777777760000000	Unknown	786	1	M/29		
PER01200010544	2000	0544	1	■■■■■■■■■■■■■■■■■■■■■■■□□□□□□□□□□□□□□□□□□□□	777777760000000	Unknown	786	1	F/0		
PER01200010578	2000	0578	1	■■□■■■■■■■■■□■■■■■□■□□□□■■■■■■■■□□□□■■■■■■■	677737207760771	LAM2	826	1	M/0		
PER01200320730	2003	0730	1	■■□■■■■■■■■■□■■■■■□■□□□□■■■■■■■■□□□□■■■■■■■	677737207760771	LAM2	826	2	M/30		HIV+
PER01200320740	2003	0740	1	■■□■■■■■■■■■□■■■■■□■□□□□■■■■■■■■□□□□■■■■■■■	677737207760771	LAM2	826	2	F/27		HIV+
PER01200320752	2003	0752	1	■■□■■■■■■■■■□■■■■■□■□□□□■■■■■■■■□□□□■■■■■■■	677737207760771	LAM2	826	2	M/30		HIV+
PER01200410194	2004	0194	1	■■□□■■■■■■■■■■■■■■■■■■■■■■■■■■□■□□□□■■■■■■■	637777777720771	H3	849	1	F/57		
PER01199910393	1999	0393	1	■■□□■■■■■■■■■■■■■■■■■■■■■■■■■■□■□□□□■■■■■■■	637777777720771	H3	849	1	M/0		
PER01200310128	2003	0128	1	■■■■■■■■■■■■□■■■■■■■□□□□■■■■□■■■□□□□■■■□■■■	777737607560731	LAM	867	1	M/39		
PER01200420191	2004	0191	1	■■■■■■■■■■■■■■■■■■□□□□□■■■■■■■■■□□□□■■■■■■■	777777017760771	T	893	2	M/30		
PER01200430193	2004	0193	1	■■■■■■■■■■■■■■■■■■□□□□□■■■■■■■■■□□□□■■■■■■■	777777017760771	T	893	3	M/56	RIF resist	
PER01200420774	2004	0774	1	■■■■■■■■■■■■■■■■■■□□□□□■■■■■■■■■□□□□■■■■■■■	777777017760771	T	893	2	M/49		HIV+
PER01200010475	2000	0475	1	■■■■■■■■□□■■■■■■■■■■■■■■■■■■■■□■□□□□■■■■■■■	776377777720771	Unknown	914	1	F/0		
PER01200110619	2001	0619	1	■■■■■■■■□□■■■■■■■■■■■■■■■■■■■■□■□□□□■■■■■■■	776377777720771	Unknown	914	1	M/0		
PER01200230335	2002	0335	1	■■■■■■■■■■■■■■■■■□■■■■■■■■■■■■■■□□□□■■■□□□■	777776777760701	X1	1080	3	M/20	INH resist	HIV+
PER01199910361	1999	0361	1	■■■■■■■■■■■■■■■□■■■■■■■■■■■■■■■■□□□□■■■■■■■	777773777760771	T1	1105	1	M/59		
PER01200010477	2000	0477	1	■■■■■■■■■■■■■■■□■■■■■■■■■■■■■■■■□□□□■■■■■■■	777773777760771	T1	1105	1	F/25		
PER01200010586	2000	0586	1	■■■■■■■■■■■■■■■□■■■■■■■■■■■■■■■■□□□□■■■■■■■	777773777760771	T1	1105	1	M/22		
PER01200230695	2002	0695	1	■■■■■■■■■■■■■■■□■■■■■■■■■■■■■■■■□□□□■■■■■■■	777773777760771	T1	1105	3	M/31	RIF resist	HIV+
PER01200210708	2002	0708	1	■■■■■■■■■■■■■■■□■■■■■■■■■■■■■■■■□□□□■■■■■■■	777773777760771	T1	1105	1	M/21		HIV+
PER01200210710	2002	0710	1	■■■■■■■■■■■■■■■□■■■■■■■■■■■■■■■■□□□□■■■■■■■	777773777760771	T1	1105	1	F/41		HIV+
PER01200330733	2003	0733	1	■■■■■■■■■■■■■■■□■■■■■■■■■■■■■■■■□□□□■■■■■■■	777773777760771	T1	1105	3	F/28	RIF resist	HIV+
PER01200410785	2004	0785	1	■■■■■■■■■■■■■■■□■■■■■■■■■■■■■■■■□□□□■■■■■■■	777773777760771	T1	1105	1	M/30		HIV+
PER01200310139	2003	0139	1	■■■■■□■■■■■■■■■■■■■■■■■■■■■■■■■■□□□□■■■■■■■	767777777760771	T1	1122	1	F/20		
PER01200110316	2001	0316	1	■■■■■■■■■■■■■■■■■■□■■■■■■■□□□□□□■□□□□■■■■■■■	777777374020771	H1	1139	1	M/40		HIV+
PER01200110323	2001	0323	1	■■■■■■■■■■■■■■■■■■□■■■■■■■□□□□□□■□□□□■■■■■■■	777777374020771	H1	1139	1	M/33		HIV+
PER01199910416	1999	0416	1	■■■□□□□□□□□□□■■■■□■■■■■■■■■□□■■■■□□□□■■■■■■■	700036776360771	X3	1150	1	M/26		
PER01200010530	2000	0530	1	■■■□□□□□□□□□□■■■■□■■■■■■■■■□□■■■■□□□□■■■■■■■	700036776360771	X3	1150	1	F/0		

PER01199910421	1999	0421	1	■■■■■■■■■■■■■■■■■■■■■■■□■■■■■■□□□□□□□□□□□□□	777777767700000	Unknown	1177	1	M/0		
PER01200410164	2004	0164	1	■■■■■■■■■■■□□□■■■■■■■■■■■■■■■■■■□□□□■■■■■■■	777617777760771	T3	1214	1	F/34		
PER01200330768	2003	0768	1	■■■■□■■□□□■■■■■■■■■■□□□□■■■■■■■■□□□□■■■□□□□	754377607760700	LAM	1220	3	M/31	INH resist	HIV+
PER01200110604	2001	0604	1	■■■■■■■■■■■■■□■■■■■■■■■■■□□□□□□■□□□□■■■■■■■	777757774020771	H1	1230	1	M/0		
PER01200210663	2002	0663	1	■■■■■■■■■■■■■■■■■■■■■■□□■□□■■■□■□□□□■■■■■■■	777777744720771	H3	1235	1	F/20		
PER01200430216	2004	0216	1	■□■■■■■■□□□■■■■■■■■■□□□□■■■■■■■■□□□□■■■■■■■	576177607760771	LAM3	1354	3	M/22	RIF resist	
PER01199920432	1999	0432	1	■□■■■■■■□□□■■■■■■■■■□□□□■■■■■■■■□□□□■■■■■■■	576177607760771	LAM3	1354	2	M/0		
PER01200320009	2003	0009	1	■■■■■■■■■■■■■■■■■■■□□□□□■■■■□■■■□□□□■■■□■■■	777777407560731	LAM	1355	2	M/37		
PER01200330102	2003	0102	1	■■■■■■■■■■■■■■■■■■■□□□□□■■■■□■■■□□□□■■■□■■■	777777407560731	LAM	1355	3	M/24	INH resist	
PER01200330130	2003	0130	1	■■■■■■■■■■■■■■■■■■■□□□□□■■■■□■■■□□□□■■■□■■■	777777407560731	LAM	1355	3	M/24	INH resist	
PER01200410160	2004	0160	1	■■■■■■■■■■■■■■■■■■■□□□□□■■■■□■■■□□□□■■■□■■■	777777407560731	LAM	1355	1	F/22		
PER01200410162	2004	0162	1	■■■■■■■■■■■■■■■■■■■□□□□□■■■■□■■■□□□□■■■□■■■	777777407560731	LAM	1355	1	M/31		
PER01200410197	2004	0197	1	■■■■■■■■■■■■■■■■■■■□□□□□■■■■□■■■□□□□■■■□■■■	777777407560731	LAM	1355	1	M/45		
PER01200430205	2004	0205	1	■■■■■■■■■■■■■■■■■■■□□□□□■■■■□■■■□□□□■■■□■■■	777777407560731	LAM	1355	3	M/48	INH resist	
PER01200430221	2004	0221	1	■■■■■■■■■■■■■■■■■■■□□□□□■■■■□■■■□□□□■■■□■■■	777777407560731	LAM	1355	3	F/40	RIF resist	
PER01199920237	1999	0237	1	■■■■■■■■■■■■■■■■■■■□□□□□■■■■□■■■□□□□■■■□■■■	777777407560731	LAM	1355	2	F/23		HIV+
PER01199910252	1999	0252	1	■■■■■■■■■■■■■■■■■■■□□□□□■■■■□■■■□□□□■■■□■■■	777777407560731	LAM	1355	1	F/19		HIV+
PER01200030270	2000	0270	1	■■■■■■■■■■■■■■■■■■■□□□□□■■■■□■■■□□□□■■■□■■■	777777407560731	LAM	1355	3	M/33	INH resist	HIV+
PER01200030271	2000	0271	1	■■■■■■■■■■■■■■■■■■■□□□□□■■■■□■■■□□□□■■■□■■■	777777407560731	LAM	1355	3	M/18	INH resist	HIV+
PER01200030272	2000	0272	1	■■■■■■■■■■■■■■■■■■■□□□□□■■■■□■■■□□□□■■■□■■■	777777407560731	LAM	1355	3	M/25	INH resist	HIV+
PER01200020278	2000	0278	1	■■■■■■■■■■■■■■■■■■■□□□□□■■■■□■■■□□□□■■■□■■■	777777407560731	LAM	1355	2	M/27		HIV+
PER01200130311	2001	0311	1	■■■■■■■■■■■■■■■■■■■□□□□□■■■■□■■■□□□□■■■□■■■	777777407560731	LAM	1355	3	M/28	INH resist	HIV+
PER01200120318	2001	0318	1	■■■■■■■■■■■■■■■■■■■□□□□□■■■■□■■■□□□□■■■□■■■	777777407560731	LAM	1355	2	M/42		HIV+
PER01199910391	1999	0391	1	■■■■■■■■■■■■■■■■■■■□□□□□■■■■□■■■□□□□■■■□■■■	777777407560731	LAM	1355	1	M/25		
PER01199910438	1999	0438	1	■■■■■■■■■■■■■■■■■■■□□□□□■■■■□■■■□□□□■■■□■■■	777777407560731	LAM	1355	1	M/0		
PER01199930439	1999	0439	1	■■■■■■■■■■■■■■■■■■■□□□□□■■■■□■■■□□□□■■■□■■■	777777407560731	LAM	1355	3	M/0	INH resist	
PER01200030514	2000	0514	1	■■■■■■■■■■■■■■■■■■■□□□□□■■■■□■■■□□□□■■■□■■■	777777407560731	LAM	1355	3	F/0	INH resist	
PER01200010517	2000	0517	1	■■■■■■■■■■■■■■■■■■■□□□□□■■■■□■■■□□□□■■■□■■■	777777407560731	LAM	1355	1	M/0		
PER01200410769	2004	0769	1	■■■■■■■■■■■■■■■■■■■□□□□□■■■■□■■■□□□□■■■□■■■	777777407560731	LAM	1355	1	M/29		HIV+
PER01200430770	2004	0770	1	■■■■■■■■■■■■■■■■■■■□□□□□■■■■□■■■□□□□■■■□■■■	777777407560731	LAM	1355	3	M/31	INH resist	HIV+
PER01200420771	2004	0771	1	■■■■■■■■■■■■■■■■■■■□□□□□■■■■□■■■□□□□■■■□■■■	777777407560731	LAM	1355	2	M/39		HIV+
PER01200320002	2003	0002	1	■■■■■■■■□□■■■■■■■■■■■■■■■■■■■■■■□□□□■■■■□■■	776377777760751	S	1356	2	M/26		
PER01200310141	2003	0141	1	■■■■■■■■□□■■■■■■■■■■■■■■■■■■■■■■□□□□■■■■□■■	776377777760751	S	1356	1	F/28		
PER01200510043	2005	0043	1	■■■□■■■■□□□■■■■■■■■■□□□□■■■■■■□□□□□□□□■■■■■	736177607700171	AFRI_2	1476	1	F/20		
PER01199910236	1999	0236	1	■■■□■■■■□□□■■■■■■■■■□□□□■■■■■■□□□□□□□□■■■■■	736177607700171	AFRI_2	1476	1	M/33		HIV+
PER01199910458	1999	0458	1	■■■□■■■■□□□■■■■■■■■■□□□□■■■■■■□□□□□□□□■■■■■	736177607700171	AFRI_2	1476	1	F/16		
PER01200410775	2004	0775	1	■■■□■■■■□□□■■■■■■■■■□□□□■■■■■■□□□□□□□□■■■■■	736177607700171	AFRI_2	1476	1	M/28		HIV+
PER01200410782	2004	0782	1	■■■□■■■■□□□■■■■■■■■■□□□□■■■■■■□□□□□□□□■■■■■	736177607700171	AFRI_2	1476	1	M/29		HIV+
PER01200530048	2005	0048	1	□■■■■■■■□□□■□■■■■■■■□□□□■■■■■■■■□□□□■■■■■■■	376137607760771	LAM3	1525	3	M/26	RIF resist	
PER01200010488	2000	0488	1	■■■■■■■■■■■■■■■■■■■■■■■■■□□□□□□■□□□□■■□□■■■	777777774020631	H1	1552	1	M/0		
PER01200310138	2003	0138	1	■■■■■■■■■■■■■■■■■■■■□□■■■■□■■■■■□□□□■■■■■■■	777777636760771	T1	1563	1	M/19		
PER01200510056	2005	0056	1	■■■□□□□□□□□□□■■■■□■■■■■■■■□■■■■■□□□□■■■■■■■	700036776760771	X3	1617	1	F/40		
PER01199920462	1999	0462	1	□■■■■■■■■■■■■■■■■■■■■■■■■□□□□□□■□□□□■■■■■■■	377777774020771	H1	1681	2	M/0		
PER01200030494	2000	0494	1	■■■■■■■■□■■■■■■■■■■■■□□□□■■■■■■■■□□□□■■■■■■■	776777607760771	LAM9	1708	3	M/0	RIF resist	
PER01200010584	2000	0584	1	■■■■■■■■■■■■□□□□□■■■□□□□■■■■■■■■□□□□■■■■■■■	777701607760771	LAM5	1999	1	M/0		
PER01199910365	1999	0365	1	■■■■■■■■■■■■■■■■■■■■□□□□■□□□□□□■□□□□■■■■■■■	777777604020771	Unknown	2028	1	M/0		
PER01200130608	2001	0608	1	■■□■■■■■■■■■■■■■■■■■□□□□■■■■■■■■□□□□□■■■■■■	677777607760371	LAM	2054	3	M/0	INH resist	
PER01200110302	2001	0302	1	■■■■■■■■□□■■■■■■■■■■■■■□□□□□■■■■□□□□■■■■■■■	776377760360771	S	2179	1	M/23		HIV+
PER01200320097	2003	0097	1	■■■■■■■■■■■■□□□□□□□□□■■■■■■■■■■■□□□□■■■■■■■	777700077760771	Unknown	2230	2	M/25		
PER01200310148	2003	0148	1	□□□□■■■■■■■■■■■■■■■□□□□□■■■■□■■■□□□□■■■□■■■	037777407560731	LAM	2274	1	M/24		
PER01200310149	2003	0149	1	□□□□■■■■■■■■■■■■■■■□□□□□■■■■□■■■□□□□■■■□■■■	037777407560731	LAM	2274	1	F/29		

PER01200410196	2004	0196	1	□□□□■■■■■■■■■■■■■■■□□□□□■■■■□■■■□□□□■■■□■■■	037777407560731	LAM	2274	1	M/22		
PER01200410202	2004	0202	1	□□□□■■■■■■■■■■■■■■■□□□□□■■■■□■■■□□□□■■■□■■■	037777407560731	LAM	2274	1	F/19		
PER01199910467	1999	0467	1	□□□□■■■■■■■■■■■■■■■□□□□□■■■■□■■■□□□□■■■□■■■	037777407560731	LAM	2274	1	M/0		
PER01200010557	2000	0557	1	□□□□■■■■■■■■■■■■■■■□□□□□■■■■□■■■□□□□■■■□■■■	037777407560731	LAM	2274	1	M/0		
PER01200310761	2003	0761	1	□□□□■■■■■■■■■■■■■■■□□□□□■■■■□■■■□□□□■■■□■■■	037777407560731	LAM	2274	1	M/22		HIV+
PER01200510049	2005	0049	1	■■■■■■■■■■■□■■■■■■■■■■■■■■■■■■□■□□□□■■■■■■■	777677777720771	H3	2381	1	M/49		
PER01200230707	2002	0707	1	■■■■□■■■■■■■■□■■■■■■■□□□□■■■■■■■■□□□□■■■■■■■	757737607760771	LAM5	2383	3	M/45	INH resist	HIV+
PER01200410177	2004	0177	1	■■■■■■■■□□□■■■■■■■■■□□□□■■■■■■□□□□□□□□■■■■■	776177607700171	LAM3	2626	1	M/18		
PER01200210332	2002	0332	1	■■■■■■■■■■■■■■■■■■■■■■■■■■■■■■□□□□□□□□□■■■■	777777777700071	Unknown	2744	1	M/43		HIV+
PER01199910415	1999	0415	1	■■■■■■■■■■■■■■■■■■■□□■■■■■■■■■□■□□□□■■■■■■■	777777477720771	H3	2885	1	F/0		
PER01200010504	2000	0504	1	■■■■■■■■□□■■■■■■■■■■■■■■■■■□■■■■□□□□■■■■■■■	776377777360771	S	2916	1	M/19		
PER01200420036	2004	0036	1	■□□□□□□□□□□□□□□□□□□□□■■■□□■■■■■■□□□□■□■■■■■	400000071760571	Unknown	2961*	2	M/34		
PER01200020523	2000	0523	1	■□□□□□□□□□□□□□□□□□□□□■■■□□■■■■■■□□□□■□■■■■■	400000071760571	Unknown	2961*	2	M/22		
PER01200330073	2003	0073	1	■■■■□■■■■■■■■■■■■■■■□□□□□■■■■□■■■□□□□■■■□■■■	757777407560731	LAM	3000*	3	F/24	INH resist	
PER01200230713	2002	0713	1	■■■■□■■■■■■■■■■■■■■■□□□□□■■■■□■■■□□□□■■■□■■■	757777407560731	LAM	3000*	3	M/39	INH resist	HIV+
PER01200330746	2003	0746	1	■■■■□■■■■■■■■■■■■■■■□□□□□■■■■□■■■□□□□■■■□■■■	757777407560731	LAM	3000*	3	M/44	INH resist	HIV+
PER01200320118	2003	0118	1	■■■■■■■■■■■■■■■■□□□□□■■■■■■□□□□■□□□□■■■■■■■	777774077020771	H3	3001*	2	M/19		
PER01200320142	2003	0142	1	■■■■■■■■■■■■■■■■□□□□□■■■■■■□□□□■□□□□■■■■■■■	777774077020771	H3	3001*	2	F/18		
PER01200420204	2004	0204	1	■■■■■■■■■■■■■■■■□□□□□■■■■■■□□□□■□□□□■■■■■■■	777774077020771	H3	3001*	2	F/26		
PER01200420206	2004	0206	1	■■■■■■■■■■■■■■■■□□□□□■■■■■■□□□□■□□□□■■■■■■■	777774077020771	H3	3001*	2	F/43		
PER01200410222	2004	0222	1	■■■■■■■■■■■■■■■■□□□□□■■■■■■□□□□■□□□□■■■■■■■	777774077020771	H3	3001*	1	F/23		
PER01200220699	2002	0699	1	■■■■■■■■■■■■■■■■□□□□□■■■■■■□□□□■□□□□■■■■■■■	777774077020771	H3	3001*	2	M/38		HIV+
PER01200320741	2003	0741	1	■■■■■■■■■■■■■■■■□□□□□■■■■■■□□□□■□□□□■■■■■■■	777774077020771	H3	3001*	2	M/40		HIV+
PER01200520801	2005	0801	1	■■■■■■■■■■■■■■■■□□□□□■■■■■■□□□□■□□□□■■■■■■■	777774077020771	H3	3001*	2	M/31		HIV+
PER01200310134	2003	0134	1	■■■■■■■■□□■■■■■■■■■■■■■■■■■□□□□□□□□□□□□□□□□	776377777000000	Unknown	3004*	1	F/32		
PER01200410791	2004	0791	1	■■■■■■■■□□■■■■■■■■■■■■■■■■■□□□□□□□□□□□□□□□□	776377777000000	Unknown	3004*	1	M/34		HIV+
PER01200310135	2003	0135	1	■■□□□□□■■■■■■■■■■■■■□■■■■■■■□■■■□□□□■■■■■■■	603777677560771	T	3005*	1	M/29		
PER01200310136	2003	0136	1	■■□□□□□■■■■■■■■■■■■■□■■■■■■■□■■■□□□□■■■■■■■	603777677560771	T	3005*	1	F/36		
PER01200410175	2004	0175	1	■□■■■■■■■■■■■■■■■■■■□□□□■■■■■■■■□□□□■■■□■■■	577777607760731	LAM4	3006*	1	M/49		
PER01200010539	2000	0539	1	■□■■■■■■■■■■■■■■■■■■□□□□■■■■■■■■□□□□■■■□■■■	577777607760731	LAM4	3006*	1	F/0		
PER01200410179	2004	0179	1	■■■■■■■■□□■■■■■■■■■■■■■■□□□□■■■■□□□□■■■■■■■	776377770360771	S	3007*	1	M/23		
PER01200410180	2004	0180	1	■■■■■■■■□■■■■■■■■■■■■■■■■□□□□□□■□□□□■■■■■■■	776777774020771	H1	3008*	1	F/20		
PER01200130314	2001	0314	1	■■■■■■■■■■■■■■■□■■■■■■■■■■■■■■■■□□□□□□■■■■■	777773777760171	T1	3009*	3	F/39	INH resist	HIV+
PER01200330745	2003	0745	1	■■■■■■■■■■■■■■■□■■■■■■■■■■■■■■■■□□□□□□■■■■■	777773777760171	T1	3009*	3	M/47	RIF resist	HIV+
PER01199910363	1999	0363	1	■■■■■■■■□□■■■■■■■■■■■■■■■■■■■■■□□□□□■■■□■■■	776377777740731	S	3010*	1	F/17		
PER01199910403	1999	0403	1	■■■■□□□■■■■■■■■■□□□□□■■■■■■■□■■■□□□□■■■□■■■	743774077560731	Unknown	3011*	1	M/23		
PER01200010515	2000	0515	1	■■■■□□□■■■■■■■■■□□□□□■■■■■■■□■■■□□□□■■■□■■■	743774077560731	Unknown	3011*	1	M/0		
PER01200210701	2002	0701	1	■■■■□□□■■■■■■■■■□□□□□■■■■■■■□■■■□□□□■■■□■■■	743774077560731	Unknown	3011*	1	M/22		HIV+
PER01200410797	2004	0797	1	■■■■□□□■■■■■■■■■□□□□□■■■■■■■□■■■□□□□■■■□■■■	743774077560731	Unknown	3011*	1	M/27		HIV+
PER01199910406	1999	0406	1	■■■■■■■■■■■■■■■■□□□□□■■■□□□□□□□□□□□□□□■■■■■	777774070000171	Unknown	3012*	1	F/0		
PER01199910457	1999	0457	1	■■■■■■■■■■■■■■■■□□□□□■■■□□□□□□□□□□□□□□■■■■■	777774070000171	Unknown	3012*	1	F/0		
PER01199930460	1999	0460	1	□■■■■■■■■■■■■■■□■■■■■■■■■■■■■■■■□□□□■■■■■■■	377773777760771	T4	3013*	3	F/0	INH resist	
PER01200010496	2000	0496	1	□■■■■■■■■■■■■■■□■■■■■■■■■■■■■■■■□□□□■■■■■■■	377773777760771	T4	3013*	1	M/0		
PER01200110618	2001	0618	1	□■■■■■■■■■■■■■■□■■■■■■■■■■■■■■■■□□□□■■■■■■■	377773777760771	T4	3013*	1	M/0		
PER01200230667	2002	0667	1	■■□■■■■■■■■■■■■■■■■■□□□□■■■■■■□□□□□□■■■■■■■	677777607700771	Unknown	3014*	3	F/28	RIF resist	
PER01200210717	2002	0717	1	□□□■■■■■■■■■■■■■■□■■■■■■■■■■■■■■□□□□■■■□□□■	077776777760701	X1	3015*	1	M/46		HIV+
PER01200310725	2003	0725	1	□□□■■■■■■■■■■■■■■□■■■■■■■■■■■■■■□□□□■■■□□□■	077776777760701	X1	3015*	1	M/31		HIV+
PER01200330723	2003	0723	1	■■□□□□□□□□□□□□□□□□□□□□□□□□□■□■■■□□□□■■■■■■■	600000000560771	Unknown	3016*	3	M/29	INH resist	HIV+
PER01200310756	2003	0756	1	■■■■■■■■■■■■■■■■■■■■■■■■■■■□□■□■□□□□■■■■■■■	777777777120771	H3	3017*	1	M/31		HIV+
PER01200430788	2004	0788	1	■■■■■■■■■■■■■■■■■■■■■■■■■■■□□■□■□□□□■■■■■■■	777777777120771	H3	3017*	3	M/38	RIF resist	HIV+
PER01200430790	2004	0790	1	■■■■■■■■■■■■■■■■■■■■■■■■■■■□□■□■□□□□■■■■■■■	777777777120771	H3	3017*	3	F/60	RIF resist	HIV+

PER01200110597	2001	0597	1	□■■□■■■■■■■■■■■■■■■■■■■■■■■■■■■■□□□□■■■■■■■	337777777760771	T1	3089*	1	F/0		
PER01200330764	2003	0764	1	□■■□■■■■■■■■■■■■■■■■■■■■■■■■■■■■□□□□■■■■■■■	337777777760771	T1	3089*	3	M/35	RIF resist	HIV+
PER01200310012	2003	0012	1	■■■□■■■■■■■■■■■■■■■■■■■■■■■■■■□■□□□□■■■□■■■	737777777720731	H3	3168*	1	F/72		
PER01199910369	1999	0369	1	■■■■■■■■■■■■■■■■■□■■■■■■■■□■■■■□■□□□□■■■■■■■	777776775720771	H3	3431*	1	M/0		
PER01200110600	2001	0600	1	■■■■■■■■■■■■■■■■■□■■■■■■■■□■■■■□■□□□□■■■■■■■	777776775720771	H3	3431*	1	M/0		
PER01199910447	1999	0447	1	■■■□□□□□□□□□□□□□■■■■□□□□■■■■■■■■□□□□■■■■■■■	700003607760771	LAM3	3432*	1	F/0		
PER01200010581	2000	0581	1	■■■□□□□□□□□□□□□□■■■■□□□□■■■■■■■■□□□□■■■■■■■	700003607760771	LAM3	3432*	1	M/0		
PER01200010566	2000	0566	1	■■■■■■■■□□□■■■■■■■■■□□□□■■■■□□■■□□□□■■■■■■■	776177607460771	LAM3	3433*	1	F/0		
PER01200010577	2000	0577	1	■■■■■■■■□□□■■■■■■■■■□□□□■■■■□□■■□□□□■■■■■■■	776177607460771	LAM3	3433*	1	F/0		
PER01200310075	2003	0075	1	■□□□□□■■■■■■■■■■■■■■□□□□■■□□□□□□□□□□□□□□■■■	407777606000031	Unknown	Orphan	1	M/48		
PER01200310095	2003	0095	1	■■■■■■■■□□□■■■■■■■■■□□□□□□□■□■■■□□□□■■■■■■■	776177600560771	T1	Orphan	1	F/27		
PER01200310112	2003	0112	1	■□□■■■■■■■■■■■■■■■■■□□■■■■■■■■■■□□□□■■■□■■■	477777717760731	T2	Orphan	1	M/50		
PER01200310127	2003	0127	1	■■■■■■■■■■■■□■■□■■■■■■■■■■■■■■■■□□□□■■■□■■■	777733777760731	T	Orphan	1	F/53		
PER01200310129	2003	0129	1	■■□□■■■■□□□■■■■■■■■■□□□□□□□□□□□□□□□□□□■■■■■	636177400000171	Unknown	Orphan	1	F/78		
PER01200410165	2004	0165	1	■■■□■■■■■■■■■■■■■■■■□□□□■■■■□■■■□□□□■■■□■■■	737777607560731	LAM6	Orphan	1	F/43		
PER01200420168	2004	0168	1	■■■□□□□□□□□□□■■■■■□■■■■■■■■■■■■■■□□□□■■■■■■■	700036777740771	X3	Orphan	2	M/30		
PER01200410173	2004	0173	1	■■■■■■■■□■■□□■■■■■■■■■■■■■■■■■■■□□□□■■■■■■■	776637777760771	T1	Orphan	1	M/50		
PER01200410178	2004	0178	1	■■■■■■■□□□■■■■■■■■■■■■■■■■□□■■■■■■□□□□■■■■□■■	774377776360751	T1	Orphan	1	M/18		
PER01200410186	2004	0186	1	■■■■■■■■■■■■■■■■■■■□■■□■■■■■■■■□□□□□□■■■■■■■	777777557700771	Unknown	Orphan	1	F/39		
PER01199910228	1999	0228	1	■■□□□□□■■■■■■■■■■■■■□□□□■■■■■■■■□□□□■■■■■■■	603777607760771	LAM1	Orphan	1	M/26		HIV+
PER01199910239	1999	0239	1	■■■■■■■■□□□■■□□□□■■■□□□□■■■■■■■■□□□□■■■□■■■	776141607760731	LAM3	Orphan	1	M/26		HIV+
PER01200010279	2000	0279	1	■■■■■■■■■■■■■■■■■■■■□□□□■■□■□■■■□□□□■■■□■■■	777777606560731	LAM6	Orphan	1	F/22		HIV+
PER01200010290	2000	0290	1	□■■■■■■■■■■■■■■■■■■■□□□□■■□□□□□□□□□□□□□□■■■	377777606000031	Unknown	Orphan	1	F/29		HIV+
PER01200110325	2001	0325	1	■■■■■■■■■■■■■■■■■■■■■■■■■□■□□□□■□□□□□□■■■■■■■	777777775020371	H3	Orphan	1	M/40		HIV+
PER01199910350	1999	0350	1	■■□■■■□□□□□□□□□□□□□□□□□□□□□□■■■□□□□■■■■■■■	670000000160771	T1	Orphan	1	M/24		
PER01199910367	1999	0367	1	■■■■■■■■■■■■■■■■■□■■■■■■■■□■■■■■■■□□□□■■■□□□□	777776775760700	X1	Orphan	1	M/0		
PER01200010478	2000	0478	1	■■■■■■■■■■■■■■■■■■■■■■■■■■■■■■□□□□□□■■■□■■■	777777777700731	Unknown	Orphan	1	F/0		
PER01200010498	2000	0498	1	■■■■■■■□□■■■■■■■■■■■■■■■■■■■■■■■□□□□■■■□■■■	774777777760731	T2	Orphan	1	M/0		
PER01200010519	2000	0519	1	■■■■■■■■■■■■■■■■■□■■■■■■■■□■■■■■■■□□□□■■□□□□□	777776775760600	X2	Orphan	1	F/0		
PER01200010570	2000	0570	1	■■■□□□■□□□□□□□□□□■■□■■■□□■■■■■■■■□□□□■□□□□□□	704003347760400	T4	Orphan	1	M/0		
PER01200110593	2001	0593	1	■■■■■■■■■■■■■□□□■■■■□■■■■■■■■■■■■□□□□■■■■■■■	777743677760771	T1	Orphan	1	M/23		
PER01200210668	2002	0668	1	■■□□□■■■■■■■■■■■■■■■■■■■■■■■■■□□□□□□■■■■■■■	617777777700771	Unknown	Orphan	1	M/25		
PER01200210671	2002	0671	1	■■■■□□□□■■■■■■■■□□□□□■■■■■■■□■■■□□□□■■■■■■■	741774077560771	T1	Orphan	1	F/36		
PER01200210705	2002	0705	1	■■■■■■■■□□■■■■■■■■■■■■■■■□□□■■■■■■■■□□□□■■■■■■■	776377761760771	S	Orphan	1	M/28		HIV+
PER01200310719	2003	0719	1	■■□□□■■■■■■■□■■■■■■■■■■■■■■■■■□■□□□□■■■■■■■	617737777720771	H3	Orphan	1	M/32		HIV+
PER01200410796	2004	0796	1	■■■■■■■■■■■■■■■■□□□□□■■■■■■■□■■■□□□□■■■□□■■	777774077560711	T1	Orphan	1	M/34		HIV+

* Clade designations according to SITVIT2 using revised SpolDB4 rules; “Unknown” designates patterns with signatures that do not belong to any of the major clades described in SITVIT2.

** Note that SITs followed by an asterisk indicates "newly created shared-type" (highlighted in yellow; n=22 containing 49 isolates) due to 2 or more strains belonging to an identical new pattern within this study or after a match with an orphan in the database. SIT designations followed by number of strains: 2961* this study (n=2); 3000* this study (n=3); 3001* this study (n=8) and USA (n=1); 3004* this study (n=2); 3005* this study (n=2); 3006* this study (n=2) and South Africa (n=1); 3007* this study (n=1) and USA (n=1); 3008* this study (n=1) and USA (n=1); 3009* this study (n=2); 3010* this study (n=1) and USA (n=1); 3011* this study (n=4); 3012* this study (n=2); 3013* this study (n=3) and USA (n=1); 3014* this study (n=1) and Argentina (n=1); 3015* this study (n=2); 3016* this study (n=1) and Panama (n=1); 3017* this study (n=3); 3089* this study (n=2) and Mexico (n=1); 3168* this study n=1, Sweden (n=1); 3431* this study (n=2); 3432* this study (n=2); 3433* this study (n=2), BRA (n=1). Finally, spoligotype orphan patterns (not yet reported in the SITVIT2 database) are highlighted in blue.

*** Drug-R code: 1, pansusceptible; 2, MDR (combined resistance to INH-RIF); 3, any other resistance; 4, XDR-TB (combined resistance to INH, RIF, fluoroquinolones, and 1 of 3 injectable drugs, i.e., capreomycin, kanamycin, or amikacin).
