# Supplementary material for: Genetic Diversity of Mycobacterium tuberculosis in Peru and Exploration of Phylogenetic Associations with Drug Resistance
Source: PLoS One. 2013 Jun 24;8(6):e65873. doi: 10.1371/journal.pone.0065873 (PMC3691179; doi:10.1371/journal.pone.0065873)
Supplement: Table S2 — A comparison of the proportion of the most predominant SITs found in Peru as compared to neighbouring countries (Brazil, Colombia) and regions (Central America and Caribbean), recorded in the SITVIT2 database as consulted on 9 April 2013. (PDF) [file pone.0065873.s004.pdf]

<u>Supplemental Table S2</u> : A comparison of the proportion of the most predominant SITs found in Peru as compared to neighbouring countries (Brazil, Colombia) and regions (Central America and Caribbean), recorded in the SITVIT2 database as consulted on 9th April 2013.

SIT	Spoligotype Description	Octal code	This study n/t (%)	Distribution in neighboring countries					
				BRAZIL n/t (%)	p values	COLOMBIA n/t (%)	p values	AMER-C/CARIB n/t (%)	p values
1	□□□□□□□□□□□□□□□□□□□□□□□□□□□□□□□□□□■■■■■■■■■	000000000003771	44/794 (5.54)	17/4556 (0.37)	<0.0001*	1/432 (0.23)	<0.0001*	65/3197 (2.03)	<0.0001*
33	■■■■■■■■□□□■■■■■■■■■■□□□□■■■■■■■■□□□□■■■■■■■	776177607760771	66/794 (8.31)	132/4556 (2.90)	<0.0001*	3/432 (0.69)	<0.0001*	74/3197 (2.31)	<0.0001*
42	■■■■■■■■■■■■■■■■■■■■□□□□■■■■■■■■□□□□■■■■■■■	777777607760771	59/794 (7.43)	401/4556 (8.80)	0.2035	93/432 (21.53)	<0.0001*	206/3197 (6.44)	0.3173
47	■■■■■■■■■■■■■■■■■■■■■■■■■□□□□□□■□□□□■■■■■■■	777777774020771	31/794 (3.9)	110/4556 (2.41)	0.0156*	2/432 (0.46)	<0.0004*	58/3197 (1.81)	<0.0004*
50	■■■■■■■■■■■■■■■■■■■■■■■■■■■■■■□■□□□□■■■■■■■	777777777720771	130/794 (16.37)	239/4556 (5.25)	<0.0001*	14/432 (3.24)	<0.0001*	160/3197 (5.00)	<0.0001*
53	■■■■■■■■■■■■■■■■■■■■■■■■■■■■■■■■□□□□■■■■■■■	777777777760771	98/794 (12.34)	322/4556 (7.07)	<0.0001*	28/432 (6.48)	<0.0013*	359/3197 (11.23)	0.3779
91	■■■□□□□□□□□□□■■■■□■■■■■■■■■■■■■■■□□□□■■■■■■■	700036777760771	22/794 (2.77)	4/4556 (0.09)	<0.0001*	7/432 (1.62)	0.2054	37/3197 (1.16)	<0.0008*
222	■■■■■■■■■■■■■■■■□□□□□■■■■■■■□■■■□□□□■■■■■■■	777774077560771	24/794 (3.02)	0/4556 (0.00)	<0.0001*	1/432 (0.23)	<0.0010*	1/3197 (0.03)	<0.0001*
1355	■■■■■■■■■■■■■■■■■■■□□□□□■■■■□■■■□□□□■■■□■■■	777777407560731	24/794 (3.02)	0/4556 (0.00)	<0.0001*	0/432 (0.00)	<0.0003*	0/3197 (0.00)	<0.0001*

* Asterisk denotes statistically significant differences (p<0.05); Pearson's Chi-square test was used when more than 80% of data had a value greater than 5, and Fisher's Exact Test for remaining data with smaller values (at least 20% of data having values less than 5).
