# Supplementary material for: Genetic Diversity of Mycobacterium tuberculosis in Peru and Exploration of Phylogenetic Associations with Drug Resistance
Source: PLoS One. 2013 Jun 24;8(6):e65873. doi: 10.1371/journal.pone.0065873 (PMC3691179; doi:10.1371/journal.pone.0065873)

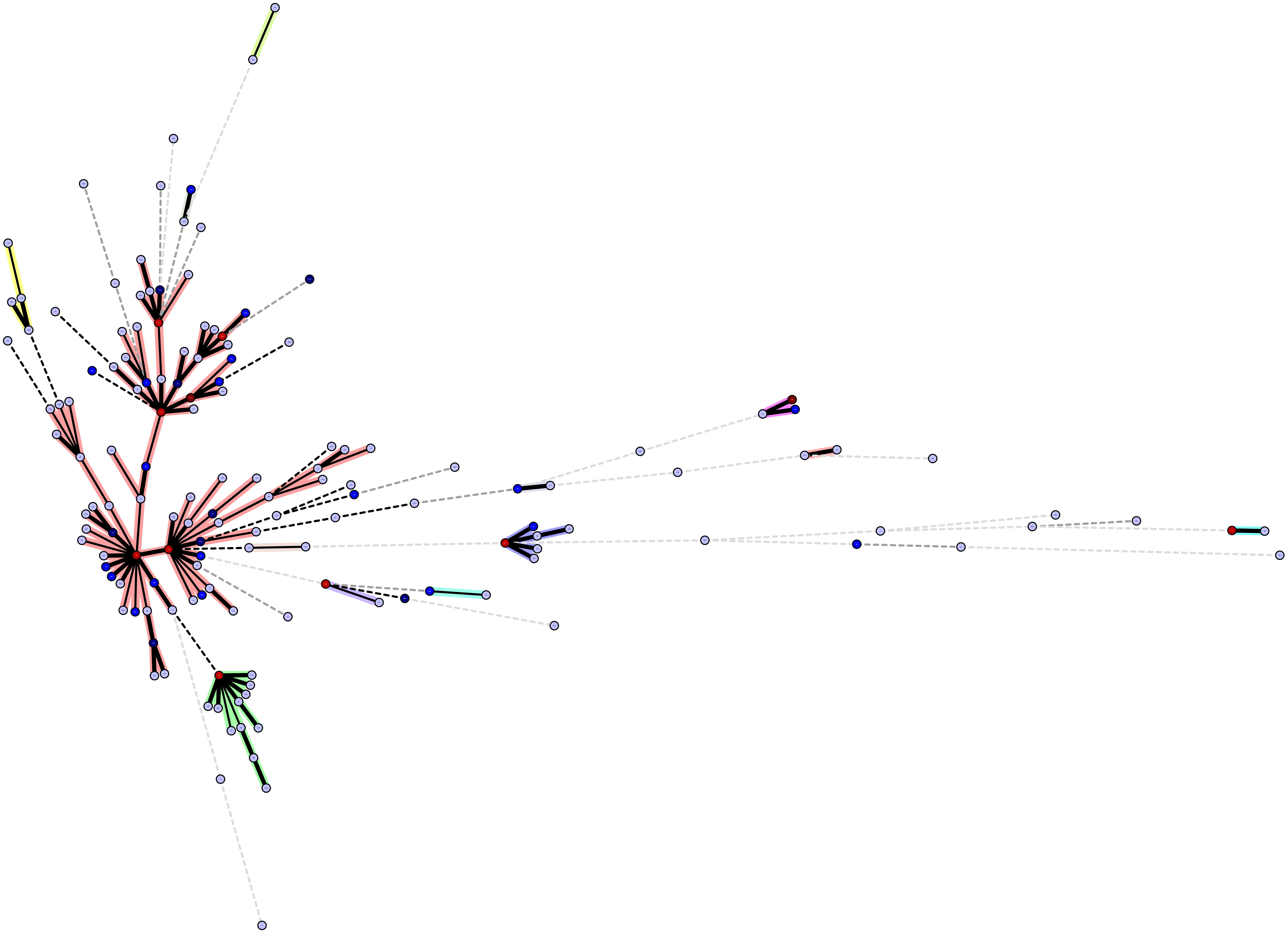

Supplement: Figure S1 — MST from manuscript Figure 1 presented in PDF format which (through zooming in) enables reading of SIT labels. (PDF) [file pone.0065873.s001.pdf]
